# Neuro‐Immune Crosstalk: Molecular Mechanisms, Biological Functions, Diseases, and Therapeutic Targets

**DOI:** 10.1002/mco2.70497

**Published:** 2026-01-22

**Authors:** Xin Guo, Hui Liu, Yu‐Jing Song, Jian‐Hua Wang, Danfeng Liu, Zhi‐Wei Zheng, Jia‐Jun Li, Boya Li, An Song, Wei He, Lei‐Lei Yang, Shuo Wang

**Affiliations:** ^1^ Department of Stomatology The First Affiliated Hospital of Zhengzhou University Zhengzhou China; ^2^ The Affiliated Stomatological Hospital State Key Laboratory Cultivation Base of Research Prevention and Treatment of Oral Diseases Jiangsu Province Engineering Research Center of Stomatological Translational Medicine Nanjing Medical University Nanjing China

**Keywords:** neuroimmunology, neuromodulation, neuro‐immune crosstalk, immunomodulation

## Abstract

The advent of neuroimmunology has dismantled the traditional doctrine of the brain's immune privilege, uncovering a sophisticated and dynamic bidirectional regulatory interplay between the nervous and immune systems. This review synthesizes pivotal advances in neuroimmunology, integrating recent anatomical and molecular discoveries to refine the understanding of neuro‐immune communication. It highlights the pathological roles of neurotransmitters, cytokines, and their signaling networks in neurodegenerative, psychiatric, and neoplastic diseases, while critically examining contested regulatory mechanisms. The review further evaluates the clinical translational potential and challenges of innovative strategies such as vagus nerve stimulation, optogenetics, multiomics sequencing, and cytokine‐targeted therapies. By integrating multidisciplinary perspectives, this review consolidates a theoretical framework for neuro‐immune research and provides insights into precision medicine for related diseases. On the basis of synthesizing existing knowledge, it proposes promising research directions, identifies priorities and potential challenges for future investigations, and emphasizes the value of neuro‐immune mechanisms in guiding therapeutic development—including target identification, design of individualized treatment strategies, and cross‐disciplinary collaborative innovation to advance clinical interventions for neuro‐immune diseases. Finally, the review delves into the recent advances and challenges in combined neuromodulation‐immunotherapy strategies.

## Graphical abstract

Neurons, immune cells, and other cellular components within the disease microenvironment (such as stromal cells and tumor cells) constitute a dynamically evolving ecosystem. Neurons directly modulate immune cell activity and inflammatory responses through the release of neurotransmitters (e.g., norepinephrine and CGRP), while also promoting tumor progression and stromal remodeling. Immune cells, in turn, feedback regulate neuronal excitability and function via the secretion of cytokines (e.g., TNF‐α and IL‐1β), and further recruit or suppress other cell types. These two systems are intricately intertwined, working in concert to regulate the immune status, nutrient supply, and tissue architecture of the microenvironment, thereby collectively promoting or inhibiting disease progression.

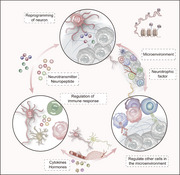

## 1 Introduction

Traditionally, the brain had long been considered a relatively isolated “immune‐privileged” region owing to the presence of the blood–brain barrier (BBB), with neuroscience and immunology being largely regarded as separate domains. However, accumulating evidence has progressively revealed complex and dynamic interactions between the nervous and immune systems, giving rise to the field of neuroimmunology. The etiology of neuroimmunology can be traced to the mid‐19th century, when research primarily focused on the isolation hypothesis between the nervous and immune systems. In the 1850s, Rudolf Virchow first described neuroglial cells, although their immunological functions remained unrecognized at that time [1, 2]. Subsequently, microglial cells were recognized as further evidence supporting the brain's considerable immunological autonomy and its segregation from the peripheral immune system. In 1885, Paul Ehrlich et al. revealed that peripherally injected dyes were restricted from entering the brain by a specialized barrier [3], which further supported the concept of “immune privilege,” and the term BBB was formally introduced in 1913 [4]. In 1921, Shirai made the crucial observation that xenografted tumor tissue implanted in mouse brains evaded rejection, whereas tumors transplanted to other sites were promptly rejected [5]. This phenomenon was subjected to systematic investigation by Peter Medawar. In 1948, he conducted skin graft experiments that demonstrated a delayed rejection response to brain tissue. Subsequently, Medawar formally proposed the “immune privilege” hypothesis, postulating that the BBB and absence of lymphatic drainage were key factors isolating the central nervous system (CNS) from the immune system [6].

However, subsequent research challenged the concept of complete “immune privilege.” Medawar demonstrated that CNS grafts could be rejected if the host animal was previously peripherally immunized [6], gradually highlighting bidirectional regulation between the nervous and immune systems. In the 1940s–50s, Geoffrey Harris established that the hypothalamus regulates pituitary function, establishing the foundation for neuroendocrine‐immune research [7, 8]. In 1974, Robert Ader discovered that conditioned reflexes could suppress immune responses. The saccharin–cyclophosphamide experiment in rats demonstrated neural regulation of immunity [9], which was later confirmed using a murine model of systemic lupus erythematosus (SLE) [10]. In 1977, Hugo Besedovsky discovered that immune activation (e.g., antigen injection) altered the firing frequency of hypothalamic neurons, further supporting immune‐to‐neural feedback regulation [11]. By 1983, studies on multiple sclerosis (MS) had investigated the distribution of T cells and their subsets within the human CNS, implicating them in disease progression [12]. In 1991, research revealed that the entry of T cells into the CNS depended on their activation state, specifically that activated T cells infiltrated the brain stochastically, further challenging the notion of immune privilege [13]. In the late 1990s, Schwartz demonstrated that following acute CNS injury, macrophages and T cells protected neurons and supported recovery [14, 15]. In 2002, the vagus nerve–α7nAChR–macrophage pathway (also termed the cholinergic anti‐inflammatory pathway [CAP]) was identified, demonstrating direct neural control of immunity [16]. Subsequent research showed that lifelong neurogenesis is supported by adaptive immune cells [17], and peripheral diseases can induce neurobehavioral disorders because inflammation‐induced cytokines impair neurogenesis [18] and disrupt higher‐order brain functions [19, 20]. Social behavior and stress responses depend on adaptive immune integrity [21]. Moreover, various immune cell types have been found to cross the BBB and directly influence brain function.

**FIGURE 1:**
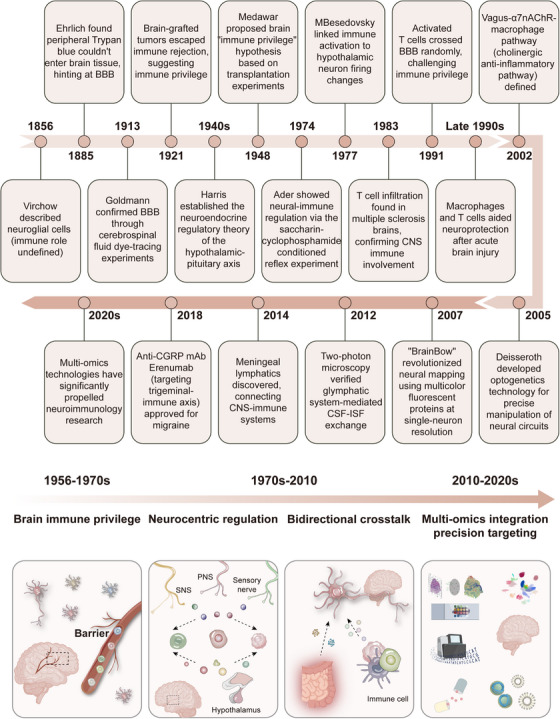
Timeline of key milestones in neuroimmunology research. This timeline outlines major discoveries that have shaped the field of neuroimmunology. During the 1950s to 1970s, a series of foundational studies established the concept of “immune privilege” in the brain. Since the late 20th century, growing evidence has revealed bidirectional communication between the central nervous system (CNS) and the immune system, including active immune surveillance by the CNS and crucial regulatory roles of immune cells in neurogenesis and neural protection. In recent years, the integration of cutting‐edge technologies—such as single‐cell multiomics, tissue clearing, and organoid models—has enabled precise dissection of neuro‐immune interactions and facilitated the development of individualized interventions, ushering the field into a new era of mechanistic and translational research. *Abbreviations*: PNS, parasympathetic nervous system; SNS, sympathetic nervous system.

Over the past 2 decades, groundbreaking discoveries of cerebral lymphatic drainage and anatomical connections between the CNS and skull have fundamentally reshaped our knowledge of neuro‐immune interactions, revealing sophisticated mechanisms that underlie tightly regulated communication between these systems. Increasing research has identified specialized immune compartments within the brain that collectively form immunological niches where immune cells can influence CNS function without disrupting neural circuitry, including the meninges, choroid plexus, and perivascular spaces [22]. Cutting‐edge technologies such as spatial transcriptomics, RABID‐seq, and Zman‐seq have enabled high‐dimensional visualization and tracking of cellular neuro‐immune crosstalk [23, 24, 25], while next‐generation red‐light‐activated optogenetics and implantable bioelectronic devices facilitate both the observation and precise manipulation of neuro‐immune circuits [26, 27]. A series of paradigm‐shifting discoveries have emerged, beginning in 2014 with Jonathan Kipnis'team, who identified meningeal lymphatic vessels, overturning the long‐held belief that the brain lacks lymphatics and establishing direct CNS–immune connections [28], Following this, in 2017, single‐cell sequencing studies revealed spatiotemporal heterogeneity of microglia in Alzheimer's disease (AD), providing novel insights into mechanisms underlying neurodegenerative diseases [29], Additionally, optogenetic studies in 2019 revealed that sympathetic nerve endings directly regulate hematopoietic stem cell (HSC) proliferation, highlighting precise neural control of immunity [26]. This has been accompanied by remarkable clinical translation, including the approval of the first anticalcitonin gene‐related peptide (CGRP) monoclonal antibody (erenumab) in 2018, which targets the trigeminal–vascular–immune axis for migraine treatment [30]. Additionally, in 2021, α7nAChR agonists (e.g., ABT‐126) demonstrated promising Phase II clinical results in schizophrenia [31], while the successful reduction of CSF α‐synuclein (α‐syn) levels in patients with Parkinson's disease (PD) has been achieved using the PD01A vaccine [32], and recent findings have demonstrated that CD22‐targeted sulesomab enhances microglial Aβ phagocytosis while suppressing neuroinflammation in AD [33]. Collectively, these discoveries illustrate the rapid translation of neuroimmunological discoveries into therapeutic interventions (Figure 1).

Despite remarkable advances in neuroimmunology, significant challenges remain in elucidating the underlying mechanisms, developing technological applications, and clinical translation. Fundamental questions persist regarding context‐dependent neuro‐immune modulation; for instance, although sympathetic activation exerts anti‐inflammatory effects during acute phases (e.g., in sepsis), it may also promote inflammation under chronic stress conditions (e.g., depression). Current technologies face limitations regarding real‐time visualization of neuro‐immune synaptic dynamics in vivo, and achieving whole‐organ‐scale dynamic imaging of neuro‐immune interactions remains a notable challenge. Clinical translation is hindered by obstacles such as suboptimal response rates, exemplified by the failure of anti‐CGRP therapies in approximately 30% of patients with migraine, indicating the existence of personalized regulatory mechanisms. To overcome these challenges, researchers have developed cutting‐edge tools, including: (1) high‐resolution spatial multiomics platforms (e.g., integrated spatial transcriptomics‐proteomics) for spatiotemporal mapping of neuro‐immune interactions to construct comprehensive “neuro‐immune connectomes”; (2) optimized organoid coculture systems, particularly brain–immune cell interaction models; and (3) advanced in vivo imaging techniques (e.g., two‐photon microscopy coupled with specific reporter systems) for real‐time observation of neuro‐immune processes. Collectively, these innovations are overcoming traditional methodological constraints and enabling unprecedented precision in mechanistic studies. At the basic research level, current efforts are focused on deciphering dynamic interaction networks among neurons, glia, and immune microenvironments, with particular emphasis on microglial heterogeneity across various neural activity states, as well as systematically investigating the regulatory roles of epigenetic mechanisms (e.g., DNA methylation, histone modifications, and noncoding RNAs) in neuro‐immune crosstalk. For clinical translation, targeted drug development is focusing on key neuro‐immune nodes such as chemokine receptors and neurotransmitter receptor‐specific modulators, alongside developing personalized therapeutic strategies based on patient‐specific neuro‐immune signatures.

Thus, this review aims to integrate multidisciplinary perspectives to systematically elucidate the core molecular mechanisms and pathophysiological functions of bidirectional neuro‐immune regulation, examine its roles across a spectrum of diseases, and critically assess the potential and challenges of novel therapeutic strategies targeting the neuro‐immune axis. It will comprehensively address several key dimensions: the intricate regulation of immune function by the neuroendocrine, autonomic, and sensory nervous systems, as well as the feedback mechanisms through which the immune system modulates neural activity via cytokines and immune cell infiltration; emerging mechanisms of neuro‐immune communication, such as direct cellular contacts, epigenetic regulation, and the gut–brain–immune axis; the central role of neuro‐immune crosstalk in major disorders including neurodegenerative, chronic pain, autoimmune, neuropsychiatric, and neoplastic diseases; and the translational prospects of innovative therapeutic approaches, including neurotransmitter receptor modulators, cytokine/anticytokine therapies, and bioelectronic medicine. It is structured to progress logically from basic mechanisms to clinical applications, beginning with a detailed exposition of the molecular basis of neuro‐immune crosstalk, encompassing both neural control of immunity and immune feedback to the nervous system. It then systematically analyzes the pivotal role of neuro‐immune interactions in neuroinflammation‐driven and immune dysregulation‐related disorders, followed by a focused exploration of the unique neuro‐immune networks within the tumor microenvironment (TME), covering both peripheral and CNS tumors. Finally, it concludes with a comprehensive outlook on therapeutic strategies targeting the neuro‐immune axis, spanning pharmacological interventions and bioelectronic medicine, thereby providing a solid conceptual framework and novel insights for future research and treatment.

## 2 Molecular Mechanisms of Neuro‐Immune Crosstalk

Bidirectional signaling between the nervous and immune systems is mediated by intricate molecular pathways that play essential roles in both normal homeostasis and disease pathogenesis. Although technological advances have enhanced our understanding of immune‐mediated regulation of neural activity, the precise pathways governing brain–immune interactions, particularly at the molecular level, remain incompletely elucidated.

### 2.1 Neural Regulation of Immune Function

**FIGURE 2:**
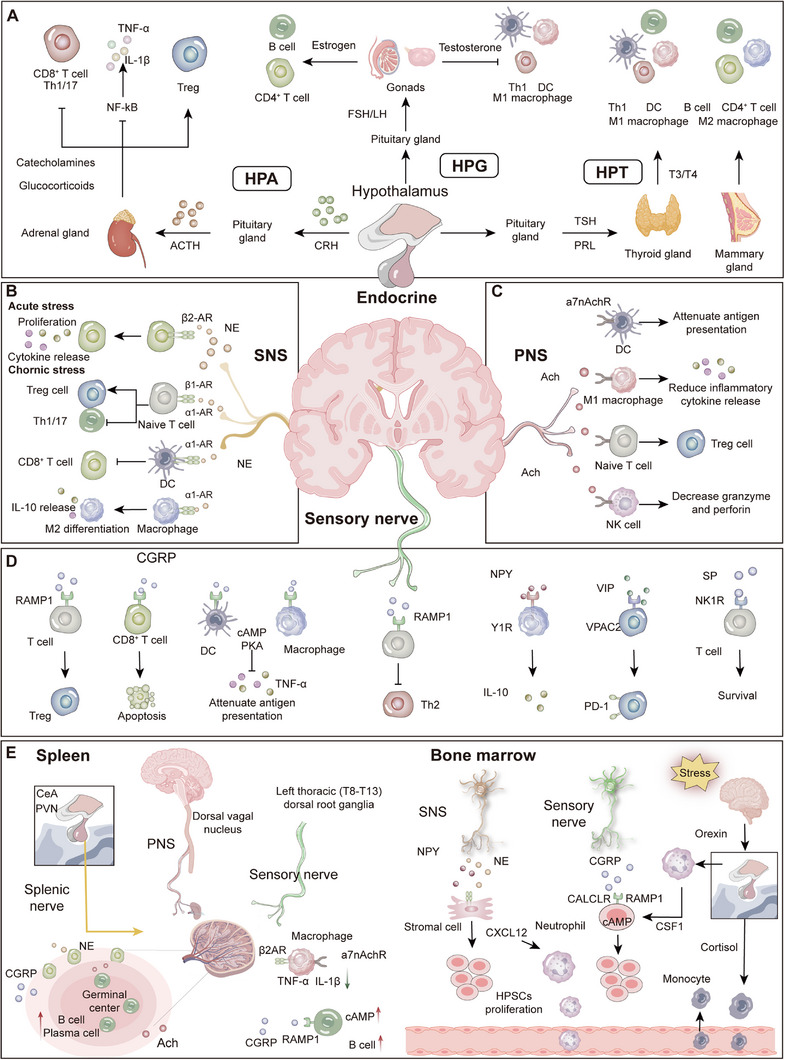
Neural regulation of the immune system. (A) Neuroendocrine immune regulation: The hypothalamic–pituitary–adrenal (HPA) axis broadly suppresses immune cell activity via the release of glucocorticoids. The hypothalamic–pituitary–gonadal (HPG) axis modulates immune responses through sex hormones (e.g., estrogen and testosterone), contributing to sex‐based differences in immunity. The hypothalamic–pituitary–thyroid (HPT) axis influences the development, activation, and metabolism of immune cells through thyroid hormones. (B) Sympathetic nervous system regulation: Mainly mediated by norepinephrine, this system modulates lymphocyte migration, proliferation, and cytokine production via α‐ and β‐adrenergic receptors. It may promote proinflammatory cytokine release during acute inflammation but suppresses excessive immune activation under chronic inflammatory conditions. (C) Parasympathetic nervous system regulation:The vagus nerve modulates peripheral immune organs such as the spleen through the “cholinergic anti‐inflammatory pathway.” Acetylcholine released from vagal terminals inhibits the release of proinflammatory factors such as TNF‐α from macrophages. (D) Sensory nerve regulation: Sensory neurons release neuropeptides (e.g., substance P and CGRP) to shape the local immune microenvironment, participating in pain, itch, and neurogenic inflammatory processes. (E) Neural control of spleen and bone marrow: Both sympathetic and sensory nerves innervate the spleen and bone marrow, where they regulate immune cell responses in the spleen and influence hematopoiesis as well as immune cell differentiation in the bone marrow through neurotransmitter signaling.

The nervous system orchestrates immune responses through multiple intricate mechanisms. Peripheral immune activity is primarily modulated via four distinct pathways: (1) the endocrine axis, (2) efferent neuronal signaling, (3) sensory neural circuits, and (4) meningeal lymphatic drainage. This regulatory activity fundamentally relies on the expression of neurotransmitter and neuropeptide receptors by immune cells, enabling direct neuronal regulation of immune cell function. Furthermore, specialized neuronal subpopulations, including peripheral sensory neurons, sympathetic and parasympathetic neurons, and enteric neurons, can directly instruct both innate and adaptive immune cells to modulate their functional activities (Figure 2).

#### 2.1.1 Neuroendocrine Regulation of Immune Function

As the central neuroendocrine regulator, the hypothalamus orchestrates immune responses through multiple intricate pathways, with the hypothalamic–pituitary–adrenal (HPA) axis being the predominant regulatory circuit. During stress responses, hypothalamic secretion of corticotropin‐releasing hormone (CRH) activates corticotropin‐releasing factor receptors 1 on pituitary corticotrophs, triggering cAMP–PKA signaling to induce adrenocorticotropic hormone release and subsequent glucocorticoid (GC; e.g., cortisol) production from the adrenal cortex. These steroid hormones exert potent immunosuppressive effects through genomic mechanisms involving GR (GC receptor)–GRE (GC response element) complexes that recruit histone deacetylases (HDACs) to epigenetically silence NF‐κB‐mediated transcription of proinflammatory cytokines (TNF‐α, interleukin [IL]‐6), while simultaneously promoting regulatory T (Treg) cell differentiation and suppressing Th1/Th17 responses to initiate rapid “anti‐inflammatory braking” [34]. Moreover, their nongenomic actions involve membrane GR‐initiated PI3K–Akt signaling that disrupts T cell receptor (TCR) activation and inhibits cytotoxic granule release in CD8^+^ T cells [35, 36]. Notably, pituitary‐resident macrophages form synapse‐like contacts with corticotrophs to deliver IL‐1β/TGF‐β signals that enhance CRH sensitivity, establishing an “immune→endocrine” feedforward loop [37], while corticotroph‐derived neuronal pentraxin‐2 (Nptx2) remotely modulates peripheral neutrophil/lymphocyte ratios, indicating that pituitary cells exert nonhormonal immunoregulatory activity [38]. The hypothalamic–pituitary–gonadal (HPG) axis similarly modulates immunity, with estrogen enhancing humoral immunity (by promoting B cell antibody production), while testosterone generally suppresses Th1 responses. Both hormones influence the T cell repertoire through thymic output regulation. Notably, the recently discovered hypothalamic–pituitary–bone marrow (BM) axis demonstrates how TME‐activated paraventricular nucleus (PVN) neurons stimulate pituitary α‐melanocyte‐stimulating hormone release, which binds melanocortin 5 receptor on hematopoietic progenitors to promote myeloid‐derived suppressor cell (MDSC) and tumor‐associated macrophage (TAM) differentiation, ultimately suppressing CD8^+^ T cell antitumor activity [39]. Other pituitary hormones that exhibit pleiotropic immunomodulation include prolactin, which enhances T/B cell function via JAK2–STAT5 while inducing macrophage M2 polarization, as well as growth hormone (GH), which maintains thymic epithelial structure through insulin‐like growth factor‐1 (IGF‐1) signaling and regulates macrophage metabolic reprogramming via mTORC1. Collectively, the neuroendocrine system employs HPA, HPG, and other axes to achieve spatiotemporally precise, hierarchical regulation of innate and adaptive immunity through hormones, neuropeptides, and neurotransmitters. However, these regulatory effects demonstrate marked cell‐type specificity and temporal dependency (e.g., β‐blockers exhibit efficacy only during early phases, and GC suppress cytokine storms acutely but promote tumor immune evasion chronically), highlighting the importance of distinguishing acute versus chronic response windows. Future research should elucidate potential universal principles governing neural control in various immune organs and develop precision therapies targeting specific cell populations or disease stages, ultimately transitioning from “broad‐spectrum immunosuppression” to “precision immune tuning” [40].

#### 2.1.2 Immunomodulation by the Autonomic Nervous System

The autonomic nervous system (primarily through its sympathetic and parasympathetic branches) exerts precise, rapid, and potent immunoregulation via the release of neurotransmitters (norepinephrine [NE], acetylcholine [ACh], etc.) that act directly on receptors expressed by immune cells. Sympathetic activation typically suppresses adaptive immunity and attenuates excessive inflammation (particularly during acute stress), while the parasympathetic system (especially via the CAP of the vagus nerve) primarily mediates rapid inhibition of excessive inflammatory responses driven by innate immunity. This neuro‐immune communication is crucial for maintaining homeostasis, coordinating stress responses, defending against pathogens, and preventing autoimmunity. Conversely, the dysregulation of this neuro‐immune communication is implicated in various diseases and has emerged as a potential therapeutic target.

##### 2.1.2.1 Sympathetic Nervous System

The sympathetic nervous system (SNS), a fundamental branch of the autonomic nervous system, establishes reciprocal communication pathways with both the parasympathetic and enteric nervous systems (ENS) to regulate immune function. Its functions are temporally dynamic: it can suppress excessive inflammation within seconds while also reshaping immune cell differentiation during chronic stress. SNS signaling relies on a two‐neuron pathway: preganglionic neurons originating from brainstem and hypothalamic nuclei as well as postganglionic neurons extending to peripheral targets such as lymphoid organs and BM [41]. NE serves as the primary SNS neurotransmitter, mediating immunomodulation through α/β adrenergic receptors (ARs); however, some sympathetic fibers corelease neuropeptides such as neuropeptide Y (NPY) to regulate immune cell function. Notably, immune cells (including T/B lymphocytes, macrophages, and dendritic cells [DCs]) ubiquitously express functional ARs, with β_2_‐AR being the most predominant [42, 43]. Meanwhile, T cells can synthesize ACh in response to NE stimulation, forming an “NE‐ACh bidirectional signaling loop” [44]. Additionally, the adrenal medulla, directly innervated by the SNS, mediates acute systemic immunomodulation through circulating epinephrine (Epi) and NE, providing a rapid stress response pathway.

SNS‐mediated immunoregulation demonstrates significant temporal and intensity dependence. For instance, transient sympathetic firing enhances CD4⁺ T cell proliferation and cytokine release within minutes via β_2_‐AR–cAMP–PKA signaling, initiating proinflammatory effects [45, 46]. Moreover, sustained NE exposure leads to β‐AR desensitization, shifting to α1‐AR or β1‐AR signaling, which upregulates Foxp3⁺ Treg cell differentiation while suppressing Th1/Th17 function and reducing proinflammatory cytokine expression [41, 47, 48, 49]. This bidirectionality stems from three factors: (1) acute NE exposure (minutes) transiently promotes inflammation, while chronic exposure (hours to days) activates negative feedback pathways; (2) immune cell subsets express distinct AR subtypes (e.g., T cells highly express β_2_‐AR, whereas macrophages enrich α1AR); and (3) NE interacts with coreleased neuropeptides (e.g., NPY) and local cytokines to induce context‐dependent effects through noncanonical pathways [50]. For B cells, β_2_‐AR agonism produces dual “activation state + costimulation”‐dependent effects: enhancing antibody production under low‐level stimulation, while suppressing it under strong Toll‐like receptors (TLR)/B‐cell receptor (BCR) signaling [51]. Regarding innate immunity, NE suppresses monocyte production of IL‐6/TNF‐α via β_2_‐AR [52] but can promote proinflammatory phenotypes through α_1_‐AR or ADRA2 activation. DCs exposed to β_2_‐AR agonists exhibit reduced IL‐12/IL‐23 expression, thus promoting Th1‐to‐Th2 polarization [53, 54]. NE promotes Treg cell differentiation via the β_2_‐AR–cAMP–PKA–CAMP‐response element binding protein (CREB) axis, where phosphorylated CREB directly binds the Foxp3 promoter [55, 56]. Aligning with sciatic nerve denervation studies demonstrating “increased interferon (IFN)‐γ levels and CD8^+^ T cell expansion” [57], these findings confirm that the SNS achieves spatially precise immunomodulation through receptor matching. BM studies reveal that chronic stress‐induced disruption of the NE–β_3_‐AR–CXCL12 axis activates HSCs, leading to myeloid skewing, immunosenescence, and tumor susceptibility [58, 59]. Furthermore, cross‐activation of the pituitary–BM–tumor axis underscores that, in addition to regulating immune organs, the SNS also reshapes distal hematopoietic niches through neural plasticity.

Overall, the SNS dynamically balances immune responses through local innervation and circulating hormonal pathways. Its dysregulation contributes to tumor immune evasion (e.g., chronic NE promotes MDSC expansion) and autoimmune pathogenesis (e.g., sympathectomy enhances local CD8⁺ T cell responses) [42, 57]. Future research should clarify AR subtype spatiotemporal expression patterns across various immune cell subsets. Meanwhile, clinical interventions should consider the “time window–receptor subtype–tissue specificity” triad; for instance, employing short‐term β_2_‐AR agonists for acute inflammation “braking” and β‐blocker/HDAC inhibitor combinations for chronic stress. Furthermore, viral tracing, ultrasonic neuromodulation, or localized drug delivery could achieve spatial targeting in key organs (e.g., the spleen and BM) to avoid systemic side effects.

##### 2.1.2.2 Parasympathetic Nervous System

Originating from the dorsal motor nucleus (DMN) and nucleus ambiguus in the brainstem, the parasympathetic nervous system (PNS) projects via the vagus nerve to thoracic and abdominal viscera. Postganglionic fibers in this system primarily use ACh as their neurotransmitter, which exerts critical immunomodulatory effects through muscarinic and nicotinic ACh receptors (nAChRs) expressed on immune cells, particularly the α7 nicotinic ACh receptor (α7nAChR) [60]. The central mechanism underlying this regulation is the well‐established “cholinergic anti‐inflammatory reflex.” Upon vagus nerve stimulation (VNS), the terminal release of ACh and other substances activates α7nAChRs on immune cells in distal tissues (e.g., spleen, visceral organs), thereby suppressing excessive inflammatory responses [61, 62]. α7nAChRs are expressed on various immune cells, including macrophages, DCs, and lymphocytes [63]. Activation of these receptors triggers intracellular signaling pathways such as JAK2–STAT3, effectively suppressing the production of proinflammatory cytokines (e.g., TNF‐α, IL‐1β, IL‐6) and activation of key inflammatory signaling pathways, including NF‐κB and NLRP3 inflammasomes [41, 63]. Notably, recent research has revealed that vagal nerve terminals release not only ACh but also various neuropeptides such as vasoactive intestinal peptide (VIP) [64]. These mediators act synergistically through dual signaling pathways by activating both α7nAChR and the VIP receptor 1 to suppress inflammation.

Breakthrough research has revealed that ACh production is not limited to neurons, as certain immune cells possess intrinsic cholinergic capacity for immunoregulation [65]. For instance, during liver regeneration, gut‐derived choline acetyltransferase (ChAT)^+^ B cells synthesize ACh to modulate Kupffer cell‐mediated (liver macrophages) IL‐6 secretion and CD8^+^ T cell‐mediated IFNγ production via α7nAChR signaling, which is crucial for postinjury hepatic repair [65]. Furthermore, B cells express multiple nAChR subtypes, with α7nAChR signaling enhancing B cell proliferation and differentiation in BM [66]. The immunomodulatory effects of α7nAChR display notable cell‐type specificity. In macrophages, receptor activation suppresses proinflammatory cytokine release and promotes M2 polarization, characterized by decreased M1 markers (CXCL9, CXCL10, and iNOS) and increased M2 markers (IL‐10, CD206) [67, 68]. In DCs, α7nAChR activation may impair antigen presentation [41]. while in Treg cells the receptor modulates their functions, with α7nAChR activation enhancing Treg cell suppressive activity to reinforce anti‐inflammatory environments [63]. Conversely, in natural killer (NK) cells, α7nAChR signaling reduces cytotoxicity by reducing the expression of effector molecules (granzyme B, perforin), NK group 2 member D (NKG2D)‐dependent cytotoxicity, and IFN‐γ secretion [69]. Accordingly, given the central role of α7nAChR in inflammation control, therapeutic strategies targeting this receptor (e.g., specific nano‐agonists) have demonstrated superior efficacy to conventional VNS in sepsis models [70]. The PNS achieves precise immunomodulation through multireceptor, multitransmitter networks, primarily mediating α7nAChR‐driven anti‐inflammatory protection during acute inflammation, while potentially exerting dual effects in specific pathologies (e.g., asthma, heart failure). Future therapeutic development should integrate tissue‐specific receptor distribution and neurotransmitter diversity to develop temporally controllable targeted therapies.

#### 2.1.3 Immunomodulation by Sensory Neurons

Sensory neurons, particularly nociceptors, represent a fundamental element of the peripheral nervous system and have garnered significant scientific attention. These neurons function as both detectors of environmental stimuli (e.g., pathogens, injury, harmful substances) and direct regulators of the immune system. They possess specialized transduction proteins (e.g., transient receptor potential vanilloid 1 [TRPV1], transient receptor potential A1, and piezo type mechanosensitive ion channel component 2 channels), which are enriched at their terminals to detect “danger signals” and subsequently influence immunity through two principal mechanisms: (1) transmitting signals to the CNS to evoke sensations (e.g., pain and itch) and (2) releasing neuropeptides (e.g., substance P [SP], VIP, NPY, and CGRP) and neurotransmitters locally via axon reflexes [71, 72]. These mediators act directly on specific receptors, including receptor activity modifying protein 1 (RAMP1/CLR), neurokinin‐1 receptor (NK‐1R), VIP receptor type 2 (VPAC2), and NPY receptor Y1 (NPY1R) expressed by neighboring immune cells (e.g., DCs, macrophages, T cells, B cells, and neutrophils), modulating their functional states and inflammatory signaling pathways (e.g., mTORC1, NF‐κB, and cAMP/PKA) to play essential roles in host defense, inflammatory responses, and tissue repair [42, 73, 74, 75, 76].

Different neuropeptides establish a sophisticated and highly context‐dependent immunoregulatory network through engagement with specific receptors. In skin barrier tissues, low‐frequency stimulation (<1 Hz) of nociceptive neurons releases CGRP, which binds RAMP1–calcitonin receptor‐like receptor (CLR) receptor complexes on DCs to promote Treg cell differentiation and suppress Th2‐mediated inflammation (e.g., in atopic dermatitis). Conversely, high‐frequency stimulation (>5 Hz) induces corelease of SP and ATP, activating mast cell degranulation and aggravating allergic responses [77]. CGRP exhibits remarkable functional duality and tissue specificity. Namely, it promotes neutrophil, monocyte, and macrophage functions during cutaneous wound healing [78]; inhibits RORγt^+^ Treg cell function via RAMP1 in the intestine [78], and facilitates Th1 differentiation via T cell‐intrinsic RAMP3 signaling during antiviral immunity (e.g., lymphocytic choriomeningitis virus infection) [78]. Additionally, CGRP suppresses antigen presentation and proinflammatory cytokine production by DCs and macrophages while enhancing IL‐10 secretion via cAMP/PKA pathways and NF‐κB inhibition [76, 79, 80]. Neuron‐derived NPY binds Y1 receptors on macrophages to inhibit mTORC1 signaling and upregulate anti‐inflammatory IL‐10 expression, thereby alleviating inflammation (e.g., in psoriasis). Intestinal sensory neurons release VIP, which directly engages VPAC2 receptors on Treg cells to induce PD‐1 expression and enhance suppressive activity, thereby suppressing autoimmune responses and offering potential therapeutic targets for inflammatory bowel disease (IBD) [81, 82]. Gut nociceptors release neuromedin U to activate ILC2s, promoting IL‐5/IL‐13‐mediated helminth expulsion, whereas tuft cell‐derived ACh suppresses ILC2 activity through α7nAChRs to prevent excessive type 2 immunity [83]. SP exerts pleiotropic effects via NK‐1Rs, promoting activated T cell survival, triggering proinflammatory cytokine secretion by macrophages, and enhancing neutrophil chemotaxis and migration [84, 85], while concurrently exerting microbiota‐dependent epithelial protection in the gut [78, 86].

Beyond paracrine signaling, recent studies have revealed direct synapse‐like connections between sensory neurons and immune cells, particularly in barrier tissues (e.g., skin, lung, intestine). For instance, in the skin, nociceptor terminals form stable structures characterized by postsynaptic density‐like features with DCs, maintained by CGRP–RAMP1 signaling. Disrupting this synaptic communication significantly attenuates Th2 inflammation in atopic dermatitis models [87, 88]. Furthermore, sensory neurons densely innervate secondary lymphoid organs (spleen, lymph nodes), influencing lymphocyte migration, differentiation, and immune responses through the release of neuropeptides (especially CGRP) to enhance antigen‐specific antibody production [89] and promote naïve T cell differentiation toward Th1 via RAMP3–CALCRL signaling [90]. Elucidating the complex mechanisms underlying sensory neuron‐mediated immunoregulation, which depend on neuropeptide types, receptors, target cells, tissue microenvironments, pathogens, and disease contexts, is essential for developing novel therapies against infections, inflammation, autoimmune disorders, and allergies. Interventions targeting specific neuropeptide pathways (e.g., NPY–Y1R, VIP–VPAC2) or neuro‐immune synapses demonstrate significant potential [91, 92]. However, the biological principles governing these neuro‐immune axes remain incompletely understood. Therefore, future research should explore how sensory neurons influence initial pathogen encounters and repeated/chronic infections, potentially uncovering their roles in influencing immune memory formation through direct peripheral effects on adaptive immunity or by encoding immunological information for CNS signaling to coordinate systemic response adaptations.

#### 2.1.4 Direct Neural Regulation of Immune Organs

##### 2.1.4.1 Spleen

The spleen serves as a critical secondary lymphoid organ that receives complex immunomodulatory input from the CNS through dedicated neural pathways. Key hypothalamic nuclei such as the PVN and central amygdala (CeA) become activated during stress or immune challenge [93], with signals transmitted to the spleen via sympathetic pathways (splenic nerve) [94]. These nerve fibers are predominantly noradrenergic (NEergic) and typically coexpress NPY (approximately 80% of celiac ganglion neurons) [95, 96, 97]. Although it was traditionally believed that the spleen lacks direct parasympathetic (vagal) innervation (with vagal modulation occurring indirectly through a “CAP” involving preganglionic vagal fibers activating postganglionic neurons in the splenic nerve) [98, 99, 100], recent research indicates the presence of direct parasympathetic innervation [101]. Additionally, sensory nerves (particularly nociceptors) have been confirmed to innervate the spleen, primarily following vascular distributions into B cell zones, originating mainly from left‐sided T8–T13 dorsal root ganglia (DRG) and displaying anatomical laterality (left‐side dominance) [89, 102].

Distinct immune cell subsets in the spleen express different AR subtypes, with B and T cells predominantly expressing β_2_‐ARs, whereas innate immune cells such as macrophages express β_2_‐ARs along with α1‐ and α_2_‐ARs [103, 104]. The neural–immune cascade starts with NE activating β_2_‐ARs on T cells, stimulating the release of ACh, which subsequently acts on α9nAChRs on adjacent B cells to promote antibody class switching and plasma cell differentiation, a process critical for establishing effective humoral immunity postvaccination [93]. Optogenetic studies have shown that activating hypothalamic CRH neurons can enhance splenic nerve firing within seconds, directly promoting B cell antibody secretion [93]. NPY serves as an important cotransmitter, with infections or inflammatory stimuli such as lipopolysaccharide (LPS) upregulating its expression in splenic nerve fibers. Single‐cell sequencing analyses demonstrate that NPY knockdown in splenic nerve ganglia alters lymphocyte proliferation and activation, suggesting that NPY represents an evolutionarily conserved neuro‐immune “language” for mitigating cytokine storms and maintaining autoimmune balance during infections [105]. Furthermore, CGRP released from splenic sensory nerves (particularly in left‐dominant regions) acts directly on B cells via CALCRL–RAMP1 receptors and cAMP signaling to enhance germinal center reactions and humoral responses [89]. Uncovering these complex CNS–splenic neuro‐immune pathways, encompassing sympathetic NE/NPY signaling, potential parasympathetic indirect pathways, sensory neural inputs, and their interactions, holds great promise for developing novel anti‐inflammatory therapies targeting splenic immune responses (e.g., enhancing vaccine efficacy or suppressing excessive inflammation). Supporting this, preliminary clinical validation indicates that splenectomy significantly reduces plasma TNF‐α levels during systemic inflammation [106, 107, 108].

##### 2.1.4.2 Bone Marrow

BM, as the primary hematopoietic organ, is subject to precise neural regulation to maintain its functional homeostasis. The BM parenchyma is densely innervated by sympathetic nerves, with nerve fibers branching along vasculature into hematopoietic zones to establish intimate interactions with HSCs, mesenchymal stem cells (BMSCs), and stromal cells, including potential synapse‐like structures [109, 110, 111, 112, 113, 114]. These sympathetic fibers primarily release NE and NPY as neurotransmitters/modulators [115]. Parasympathetic fibers are sparse in BM with unclear local effects; however, central cholinergic signaling (e.g., hypothalamic muscarinic receptor 1 activation) can indirectly influence HSC mobilization through HPA axis‐mediated GC release [116]. Notably, sensory nerves (particularly nociceptors expressing neuropeptides such as CGRP) also innervate BM, primarily in subcapsular regions, with advanced 3D imaging revealing close appositions between sensory fibers (especially those originating from left‐sided T8–T13 DRG with lateralization dominance) and immune cells, including B cells [117, 118].

The brain–BM axis is governed by specific neuronal populations in higher brain centers such as the hypothalamic PVN and CeA. Optogenetic activation of CRH neurons in the PVN/CeA can enhance BM sympathetic nerve firing within seconds, directly promoting HSC proliferation [119] and highlighting rapid neural control of hematopoiesis. Sympathetically released NE is a key mediator of HSC mobilization by acting on β‐ARs (primarily β2 and β3 subtypes) on BM stromal cells (e.g., BMSCs). This induces circadian downregulation of the expression of CXCL12, the critical chemokine anchoring HSCs in BM niches, which is essential for neutrophil and progenitor release into circulation [113, 114]. During aging, diminished sympathetic innervation and impaired β3‐adrenergic signaling play a key role in HSC dysfunction (characterized by reduced regenerative capacity and multilineage potential) [58]. Coreleased NPY promotes HSC mobilization via matrix metallopeptidase 9 (MMP‐9) activation [115]. Additionally, BM‐resident sympathetic neurons with cholinergic phenotypes are crucial for preventing progenitor exhaustion and maintaining hematopoietic homeostasis [120]. In contrast to indirect sympathetic effects via stromal cells, nociceptive sensory fibers directly influence HSCs through CGRP release, which binds RAMP1/CALCRL receptor complexes on HSCs to activate Gαs signaling (e.g., increasing intracellular cAMP expression) and promote HSC mobilization from niches [118].

Despite these mechanistic advances, significant challenges remain; for instance, the ultrastructure of sensory nerve–BM cell synapses remains unresolved, and real‐time monitoring technologies for neuropeptide (e.g., NE, CGRP) spatiotemporal dynamics in BM microenvironments are lacking. Although neuroregulation‐based strategies (e.g., BM transplantation optimization, β‐AR targeting, and sensory nerve/CGRP pathway modulation) show promise for improving hematopoiesis (e.g., restoring leukocyte output balance during aging or disease) [58, 121], overcoming these technical limitations and fully elucidating mechanistic complexities are essential for achieving precise personalized regulation.

### 2.2 Immunomodulation of Neural Function by the Immune System

The immune system exerts profound effects on nervous system function through four main mechanisms: cytokine storms, immune cell infiltration, gut–brain axis (GBA) metabolites, and neuro‐immune circuits. Although therapeutic strategies targeting IL‐6 or VNS are promising, significant challenges remain regarding BBB penetrance and spatiotemporal specificity of modulation.

#### 2.2.1 Cytokine‐Mediated Neuromodulation

Cytokines are key immune signaling molecules that influence CNS structure and function through multiple pathways, especially during inflammatory conditions. During systemic inflammation or localized CNS disorders (e.g., stroke, neuroinflammation), proinflammatory cytokines (TNF‐α, IL‐1β, and IL‐6) enhance BBB permeability by disrupting tight junction (TJ) proteins (e.g., occludin) in endothelial cells. IL‐6 crosses the BBB via receptor‐mediated transcytosis or saturable transport systems [122, 123, 124, 125]. Once within the CNS, whether through BBB penetration or produced by activated glia (microglia, astrocytes) and infiltrating immune cells, cytokines and associated chemokines (CCL2, CXCL10) exert diverse effects. These include promoting neuronal apoptosis, exacerbating oxidative stress, reducing synaptic plasticity (e.g., suppressing long‐term potentiation), and recruiting peripheral immune cells to amplify inflammatory cascades, thus playing a key role in neurodegeneration [126, 127, 128]. T cell‐derived cytokines (IFN‐γ, IL‐17) and B cell‐derived antibodies further modulate microglial activation or bind neuronal surface receptors to influence synaptic function, forming complex neuro‐immune networks [129]. Alternatively, peripheral cytokines may act in areas lacking the BBB (e.g., circumventricular organs), stimulate prostaglandin/nitric oxide (NO) release from cerebrovascular endothelia or perivascular macrophages, or activate IL‐1R/TLR4‐expressing vagal afferents that project to the nucleus tractus solitarius (NTS) and HPA axis [130].

The IL‐17 family (IL‐17A, IL‐17C) are well‐characterized “neuromodulatory” cytokines that bind IL‐17RA/RE receptors on basolateral amygdala neurons, enhancing N‐methyl aspartic acid (NMDA) receptor function via Act1–TRAF5 signaling to increase intracellular Ca^2^⁺ and neuronal excitability, thus promoting anxiety‐like behaviors [131]. Meningeal γδ T cells are a major source of IL‐17A in the CNS, establishing functional connections with hippocampal neurons through basement membrane channels. Their IL‐17A activates neuronal IL‐17RA to promote postsynaptic density protein‐95 phosphorylation and AMPA receptor membrane trafficking, enhancing synaptic plasticity and contributing to depression‐like behaviors in chronic stress models [132]. In primary somatosensory cortex dysgranular zone (S1DZ) neurons, IL‐17 suppresses neuronal activity, a potentially protective mechanism against autism‐like behaviors (repetitive actions, social deficits) associated with S1DZ hyperactivation [131, 133, 134], with IL‐10 counteracting IL‐17‐induced changes in excitability [131]. IL‐6 enters the CNS via saturable transport or transcytosis to alter neuronal excitability and promote neurotoxicity, with IL‐6 inhibitors (e.g., satralizumab, tocilizumab) proving efficacious for neuromyelitis optica (NMO) spectrum disorder [126, 135]. Stress‐induced intestinal IL‐22 crosses into septal brain regions to inhibit neuronal activation, which is both necessary and sufficient for alleviating anxiety‐like behaviors in mice [136]. Moreover, the alarmin IL‐33, through its IL‐33–ST2–AKT axis, critically regulates microglial metabolic adaptation and phagocytic function during neurodevelopment [137].

Beyond immune regulation, cytokines serve as vital neuromodulators that participate in neuronal development, synaptic plasticity, homeostasis maintenance, and adaptive behaviors (stress responses, social interaction) under physiological conditions [138]. Elucidating cytokine‐mediated neural regulation mechanisms provides novel therapeutic targets for neuropsychiatric and neuro‐immune disorders. Future research should clarify the functions of cytokines within specific neural circuits and the role of individual variability to advance the development of precision neuro‐immune therapies [135].

#### 2.2.2 Emerging Insights into Microglial Heterogeneity

In addition to their classical protective functions, microglia, which are CNS intrinsic immune cells, are now recognized as dynamic contributors to the development, maintenance, and modulation of neural circuits, employing sophisticated mechanisms to dynamically modulate synaptic plasticity, neuronal activity, and metabolic homeostasis. Under physiological conditions, microglia constitutively secrete various neurotrophic factors, including brain‐derived neurotrophic factor (BDNF) and IGF‐1, providing critical neuronal survival support and facilitating synaptic plasticity essential for cognitive functions such as learning and memory [135]. Recent studies have revealed that microglia‐derived platelet‐derived growth factor B (PDGFB) signaling through neuronal PDGFRα receptors upregulates the potassium channel subunit Kv4.3, enhancing potassium currents to prevent hyperexcitation of presympathetic neurons in the hypothalamic PVN and maintain neuronal electrophysiological homeostasis [139]. Moreover, microglia express receptors for several neuronal signaling molecules, including neurotransmitters and neuropeptides, enabling them to sensitively detect and respond to neuronal activity [140, 141]. This neuroglial communication guides dynamic microglial process extension, allowing continuous synaptic surveillance. During critical developmental periods, microglia sculpt neural connectivity through complement‐mediated pruning (C1q–C3 pathway) of redundant or weak synapses, a process tightly regulated by neuronal signals such as CX3CL1, to maintain the excitatory/inhibitory (E/I) balance [142].

Microglial responses profoundly influence disease progression in neurological disorders and exhibit context‐dependent neuroprotective or neurotoxic effects. In early AD, specific microglial subsets (e.g., disease‐associated microglia) activate protective mechanisms through triggering receptor expressed on myeloid cells 2 (TREM2)‐mediated, ApoE‐dependent amyloid‐β (Aβ) phagocytosis to delay plaque deposition [143, 144]. However, chronic pathological stimulation drives microglial dysfunction, characterized by excessive proinflammatory cytokine (TNF‐α, IL‐1β), reactive oxygen species (ROS), and glutamate secretion, which collectively impair synaptic plasticity, exacerbate neuronal damage, and hinder pathological protein clearance [140, 145]. Metabolic reprogramming underlies this phenotypic shift: proinflammatory microglia enhance glycolysis via hexokinase 2 [146], while anti‐inflammatory/repair phenotypes rely on peroxisome proliferator‐activated receptor γ coactivator 1α (PGC‐1α)‐mediated mitochondrial biogenesis and oxidative phosphorylation (OXPHOS) activation [146, 147]. Notably, microglial replacement therapy using healthy HSCs has shown preliminary success in restoring microglial function [144]. Despite these advances, critical challenges remain in optimizing targeted delivery, developing personalized intervention strategies, and comprehensively characterizing human microglial heterogeneity using advanced imaging‐omics approaches.

### 2.3 Mechanisms of Neuro‐Immune Communication

#### 2.3.1 Direct Cellular Contacts Between Neurons and Immune Cells

In addition to soluble signaling molecules, neurons can establish direct synapse‐like connections with immune cells. In peripheral organs, sympathetic nerve terminals form specialized synapse‐like structures with T cells, B cells, and macrophages within secondary lymphoid organs, such as the spleen and lymph nodes [148]. Advanced three‐dimensional clearing imaging techniques have revealed that sympathetic fibers originating from left‐sided T8–T13 DRG penetrate deep into the splenic white pulp along arterial branches, forming direct contacts with ChAT⁺CD4⁺ T cells. These T cells release ACh upon NE stimulation, subsequently inhibiting the secretion of IL‐1β and TNF‐α from macrophages through α7nAChR activation [149]. Similarly, sensory CGRP⁺ fibers establish CALCRL–RAMP1 synapses with B cells in the splenic marginal zone to enhance germinal center reactions and antibody production. These specialized structures minimize signal transmission distances and enable neurotransmitters to act with high spatial precision in specific immune cell populations [149]. In the skin, nociceptive nerve terminals form stable synapse‐like connections with DCs featuring postsynaptic density‐like structures, which are maintained by CGRP–RAMP1 signaling to directly drive Th2‐type inflammatory responses, such as those observed in atopic dermatitis [150]. Within the CNS, while the healthy brain maintains strict barriers, including the BBB, choroid plexus epithelium, and meningeal arachnoid barrier, to restrict cellular trafficking, these barriers become permeable during pathological states (e.g., neuroinflammation and demyelinating diseases), allowing selective immune cell infiltration [151]. For example, Th17 and CD8^+^ T cells can directly cross the BBB and influence cerebral immunity [152]. Particularly noteworthy is the recently discovered “velum interpositum,” an extension of the pia mater beneath the hippocampus that serves as a privileged entry route for myeloid cells (e.g., meningeal macrophages) into the brain parenchyma, directly modulating local neuroinflammation in demyelinating diseases [153, 154].

Tunneling nanotubes (TNTs) have emerged as crucial conduits for direct material and information exchange within neuro‐immune–glial networks. Recent studies highlight the multifaceted roles of TNTs in facilitating intercellular communication: DCs can directly transfer antigen peptide–MHC complexes to T cells via TNTs for efficient immune activation, while proinflammatory cytokines (TNF‐α, IL‐1β) rapidly propagate through TNTs among glial cells (microglia, astrocytes) to amplify neuroinflammatory cascades [155, 156]. Neurons utilize TNTs to transfer damaged mitochondria to neighboring microglia for mitophagic clearance, alleviating oxidative stress, whereas microglia can reciprocally deliver healthy mitochondria to neurons through TNTs to restore energy metabolism and reduce ROS levels in α‐syn pathology [157]. Intriguingly, in TMEs, glioma cells hijack this mechanism by acquiring neuronal mitochondria via TNTs to enhance their invasiveness [155, 157]. Despite these advances, significant knowledge gaps remain regarding TNT biology, including their transient nature (lasting only minutes in vivo), technical challenges in deep‐tissue live imaging, molecular triggers for F‐actin nucleation during formation, and the mechanisms governing cargo selectivity during transport. Substantial challenges persist in achieving high‐resolution, long‐term, and noninvasive monitoring of specific cellular interactions (particularly TNTs and deep‐brain synapses) within complex living systems. Similarly, it is difficult to develop spatiotemporally precise interventions to block detrimental TNT‐mediated transfers or enhance protective synaptic connections. Overcoming these limitations is crucial for elucidating the pathophysiological significance of neuro‐immune interactions and developing targeted therapeutic strategies.

#### 2.3.2 Epigenetic Regulation Mechanisms

Epigenetic mechanisms are critical molecular hubs linking environmental stimuli (e.g., stress and infection) to neuro‐immune homeostasis, dynamically reshaping chromatin landscapes to precisely orchestrate stress responses, inflammatory reactions, and the formation of long‐lasting “immune memory.” In stress integration centers such as the hypothalamic PVN and hippocampus, GRs recruit HDACs HDAC2/3 or acetyltransferase p300 to modulate chromatin accessibility at both pro‐ and anti‐inflammatory gene loci [158]. Chronic stress induces abnormal H3K9 hyperacetylation and DNA hypomethylation at the GR promoter regions, downregulating GR expression, and resulting in sustained HPA axis hyperactivity with concomitant peripheral low‐grade inflammation [159]. This epigenetic programming exhibits remarkable persistence in glial cells: astrocytes in stroke or AD models display elevated tri‐methylation of lysine 4 on histone H3 (H3K4me3) and reduced histone 3 lysine 9 trimethylation (H3K9me3) levels, driving prolonged expression of vascular endothelial growth factor (VEGF) and IL‐6 to establish a proinflammatory “neuroinflammatory memory” [160], while microglia maintain LPS‐induced DNA methylation reprogramming for at least 6 months, significantly altering subsequent susceptibility to Aβ deposition [160]. Glial metabolic states are directly correlated with epigenetic modifications: proinflammatory stimuli (LPS) enhance glycolysis and lactate accumulation to inhibit HDAC3 activity and open proinflammatory chromatin, whereas anti‐inflammatory signals (IL‐4) promote PGC‐1α‐mediated mitochondrial respiration and α‐ketoglutarate production to facilitate ten‐eleven translocation 2 (TET2)‐dependent demethylation (5mC→5hmC) at anti‐inflammatory loci like Arg1 [161]. Tet2‐mediated 5hmC regulates inflammatory gene expression by reshaping the 3D genome architecture through altered CCCTC binding factor (CTCF) binding, which is a dysregulated mechanism in aging brains [162, 163]. In peripheral immunity, neural signals rapidly switch immune phenotypes via epigenetic mechanisms: splenic sympathetic NE through β_2_‐AR activates PKA to transiently induce histone H3S10 phosphorylation and p65 acetylation, silencing TNF‐α while activating IL‐10; however, sustained NE stimulation recruits HDAC3 to the IL‐10 promoter for silencing and TNF‐α reactivation [164]. Chronic stress drives DNA methyltransferase 3 alpha (DNMT3a)‐mediated DNA methylation rewiring in HSCs, promoting granulocyte‐monocyte expansion and GC‐resistant proinflammatory subsets [159]. Notably, targeting epigenetic enzymes (e.g., HDAC3 inhibition by Trichostatin A in stroke penumbra neurons) can mitigate apoptotic gene silencing and infarct volume [161]. Collectively, these findings establish epigenetic coding as a molecular foundation for neuro‐immune adaptation, long‐term memory formation, and pathological persistence.

Epigenetic regulators may exert opposing effects across different cell types, tissue contexts, or disease stages. For instance, DNMT1 inhibition promotes axonal regeneration in neurons, while potentially accelerating glioma proliferation. Similarly, IL‐4‐induced anti‐inflammatory reprogramming (TET2 activation and histone H3 lysine 27 acetylation (H3K27ac) enrichment at anti‐inflammatory genes) underlies neuroprotection, but may exacerbate autoimmune attacks by upregulating MHC II in meningeal B cells [165]. This complexity necessitates cell‐ and context‐specific understanding of epigenetic regulation. Epigenetics provides crucial insights into the plasticity of neuro‐immune interactions, serving both as a central mediator of pathogenesis and as a promising therapeutic target.

### 2.4 The Gut–Brain–Immune Axis

**FIGURE 3:**
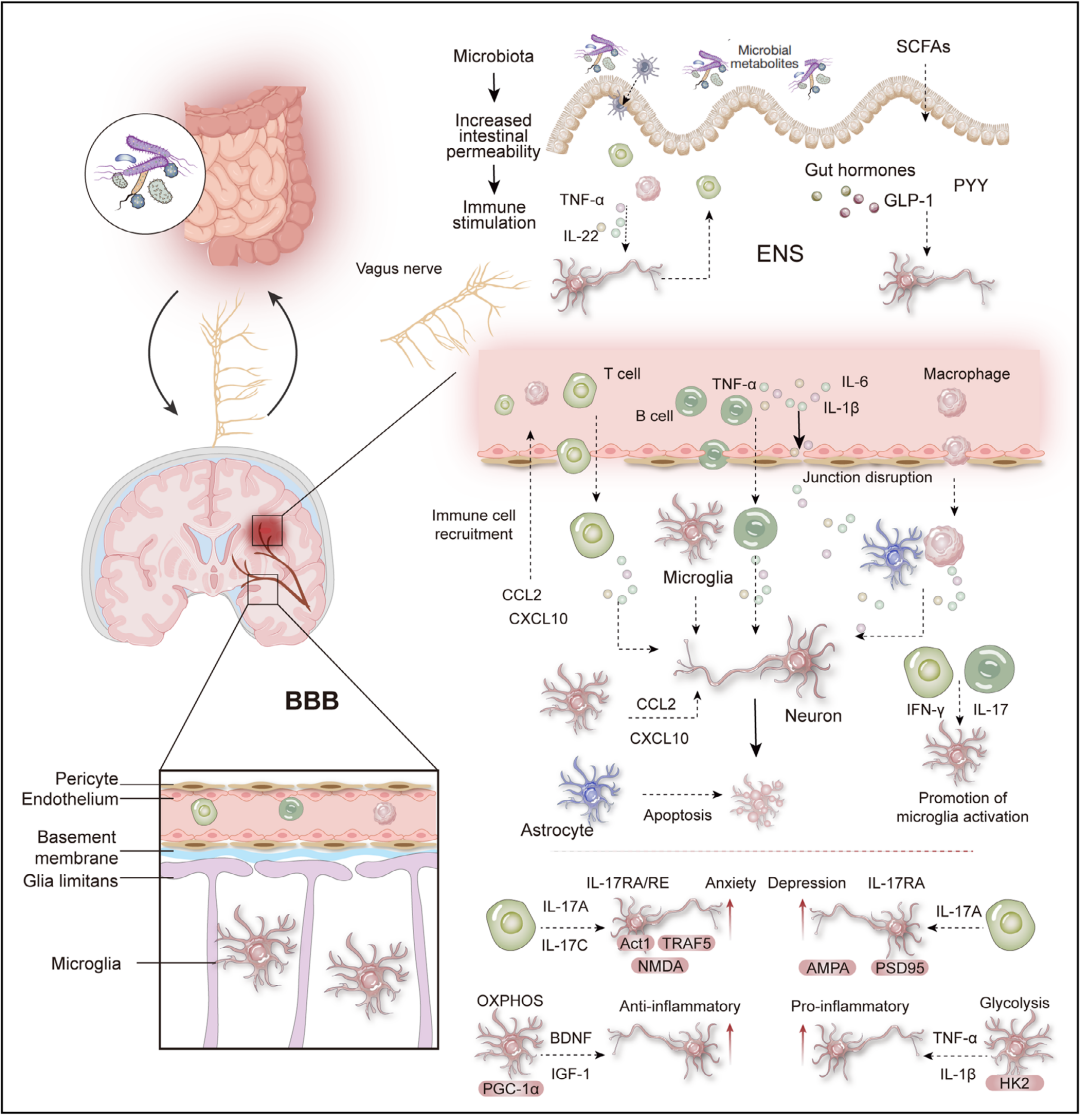
Immunoregulation of the nervous system. The immune system modulates central nervous system (CNS) function and pathological processes through multiple mechanisms: Proinflammatory cytokines (e.g., TNF‐α, IL‐1β, IL‐6) disrupt tight junctions of the blood–brain barrier, increasing its permeability and facilitating the infiltration of peripheral immune cells into the brain parenchyma. These infiltrating immune cells, together with activated microglia, contribute to a neuroinflammatory milieu characterized by sustained release of inflammatory mediators. This process alters neuronal metabolic activity, induces synaptic dysfunction and oxidative stress, and ultimately leads to neuronal damage or apoptosis. The gut–brain axis serves as a critical regulatory pathway through which gut microbiota and their metabolites influence neuroinflammation and cognitive behavior via immune, neural, and endocrine routes. Microbial metabolites such as short‐chain fatty acids (SCFAs) regulate microglial maturation and function, while dysbiosis exacerbates neuropathology by promoting systemic inflammation.

The GBA is a complex bidirectional network, interlinking the CNS, ENS, microbial communities, metabolic byproducts, immune elements, and neural pathways to facilitate cross‐system communication, particularly through the vagus nerve. Gut microbiota are the core components of this axis and their composition and function profoundly influence systemic homeostasis [63, 166, 167] (Figure 3). Intestinal vagal afferents process sensory inputs (including microbial metabolites) and relay signals to the brainstem nuclei (e.g., NTS), subsequently modulating brain function, including behavior, mood, and cognition, autonomic output, inflammatory responses, and gut function [168, 169, 170]. Microbiota dysbiosis (e.g., short‐chain fatty acid [SCFA] depletion and free fatty acid receptor 2/3 dysregulation) disrupts this fine‐tuned regulation, promoting proinflammatory microenvironments such as those found in colorectal cancer and compromising health [171]. GBA dysfunction is associated with multiple disorders, and microbiota and their metabolites influence brain function via vagal signaling and immune mediators (e.g., IL‐22), offering novel perspectives on stress, mood disorders (depression, anxiety), and neurodegeneration (PD) [136]. Future research must decipher specific “microbe–metabolite–immune–neural” mechanisms within this axis, overcoming microbiome complexity, to develop precise interventions (targeted metabolite supplementation, pre/probiotics, VNS, and fecal microbiota transplantation) for oncological, neuropsychiatric, and neurodegenerative diseases [63, 171, 172].

## 3 Neuro‐Immune Crosstalk in Nonmalignant Diseases

This section systematically examines the pivotal role of neuro‐immune interactions in the pathogenesis and progression of a spectrum of nonmalignant diseases. We begin with neuroinflammation‐driven disorders, including neurodegenerative diseases (such as AD, PD, Huntington's disease [HD], amyotrophic lateral sclerosis [ALS], and MS) and chronic pain conditions (such as fibromyalgia [FM] and migraine), highlighting how dysregulated neuro‐immune communication initiates and sustains pathological processes. We then explore immune dysregulation‐driven disorders, encompassing autoimmune diseases (such as rheumatoid arthritis [RA], SLE, and psoriasis) and neuropsychiatric conditions (such as depression and schizophrenia), emphasizing the bidirectional interplay between immune activation and neural dysfunction. Finally, we discuss the environment–immune–nervous system axis, focusing on allergic/asthmatic and gastrointestinal diseases (such as irritable bowel syndrome [IBS] and IBD), where environmental triggers disrupt neuro‐immune homeostasis. Throughout, we identify shared mechanisms, current research challenges, and emerging therapeutic strategies targeting the neuro‐immune interface.

### 3.1 Neuroinflammation‐Driven Disorders

Dysregulated neuro‐immune interactions are key drivers of neuroinflammation, that trigger and maintain a self‐sustaining inflammatory cycle in the CNS. Chronic neuroinflammation significantly impairs neuronal function, causing damage and contributing to core symptoms of multiple disorders. Dysregulated neuro‐immune communication is a key driver in the onset, advancement, and persistence of various conditions, including neurodegenerative diseases and chronic pain disorders. The following sections describe the underlying pathological mechanisms behind these disorders and highlight the therapeutic value of targeting neuro‐immune interactions.

#### 3.1.1 | Neurodegenerative Diseases

**FIGURE 4:**
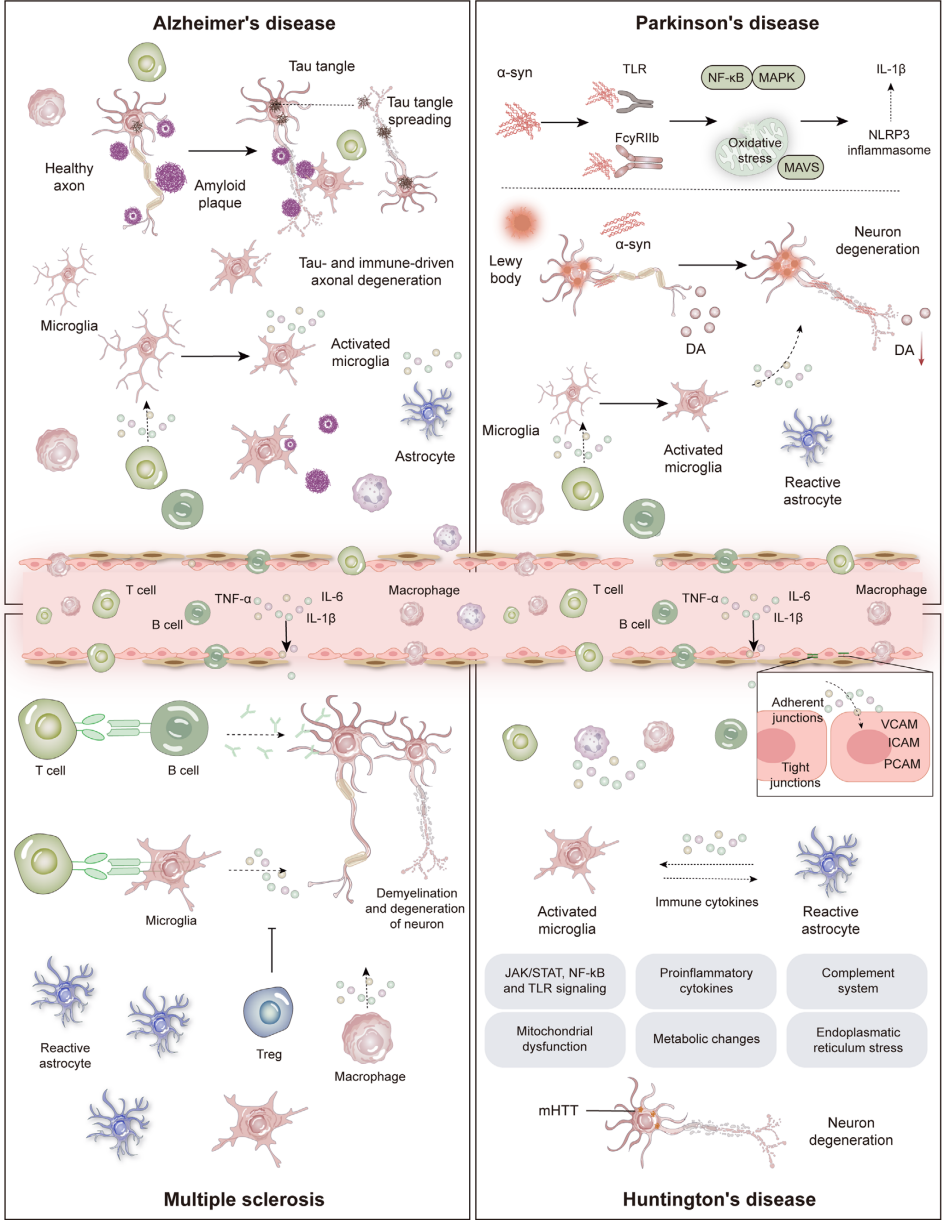
Dysregulation of neuro‐immune crosstalk in neurological disorders. Alzheimer's disease (AD): Aβ deposition and hyperphosphorylated Tau protein activate microglia and astrocytes, triggering sustained neuroinflammation. Impaired Aβ clearance by microglia and their sustained release of proinflammatory factors such as IL‐1β and TNF‐α contribute to synaptic dysfunction and accelerated cognitive decline. Parkinson's disease (PD): Aberrant aggregation of α‐synuclein activates microglia, leading to the degeneration of dopaminergic neurons in the substantia nigra. Infiltration of peripheral immune cells and release of inflammatory cytokines further amplify neuroinflammatory responses, which—together with mitochondrial dysfunction and oxidative stress—mediate neuronal loss. Multiple sclerosis (MS): Autoreactive T and B cells cross the blood–brain barrier and recognize central myelin antigens, initiating demyelination. Activated microglia and astrocytes recruit additional immune cells, fostering an inflammatory microenvironment that results in axonal damage and neurological impairment. Huntington's disease (HD): mHTT expression drives chronic activation of microglia and astrocytes, promoting the release of proinflammatory cytokines including IL‐6 and TNF‐α, and exacerbating neuroinflammation in the striatum and cortex. Peripheral immune cell infiltration and altered cytokine profiles further contribute to neuronal dysfunction and degeneration. *Abbreviations*: Aβ: β‐amyloid; mHTT: Mutant huntingtin.

Neurodegenerative diseases are characterized by a progressive nervous system dysfunction resulting from neuronal degeneration. Emerging evidence suggests that neuro‐immune crosstalk plays a pivotal role in the initiation and progression of these disorders. A sophisticated bidirectional interplay exists between the neural and immune systems, which typically function in a coordinated manner to sustain the physiological equilibrium of the body. However, this balance is disrupted in neurodegenerative diseases. Factors such as the abnormal activation of immune cells, excessive release of proinflammatory factors, and dysregulation of neuro‐immune signaling pathways can mediate neuronal injury and death, accelerating the disease process. Such diseases include AD, PD, and HD (Figure 4).

##### 3.1.1.1 Alzheimer's Disease

AD, a prevalent neurodegenerative condition, manifests as a progressive deterioration of the CNS, wherein the underlying pathology directly targets both neurons and associated support cells (notably glia), inducing functional impairments via multifactorial neurobiological mechanisms [173].

The core pathological process of AD manifests as complex neuro‐immune network dysregulation. In this network, microglia and astrocytes, key immune sentinels of the CNS, are activated by abnormally accumulated proteins such as Aβ peptides, derived from abnormal digestion of amyloid precursor protein, and phosphorylated tau proteins, derived from intrinsic pathological changes [174]. Tau hyperphosphorylation changes its role from maintaining neuronal microtubule stability to that of a key causative agent in the formation of neurofibrillary tangles [175]. Glial cell activation triggers the secretion of proinflammatory factors (TNF‐α, IL‐1β, IL‐6) that both directly intensify neuronal injury and induce pathological tau hyperphosphorylation [176]. Microglial activation is often chronic, and their cytokine release may be regulated by local neuronal signaling (e.g., the CX3CL1–CX3CR1 axis) [177]. Peripheral infiltrating macrophages enter the brain owing to the increased permeability of the BBB caused by the AD pathological state. Conversely, these peripheral macrophages can potentiate neuroinflammation and accelerate tau hyperphosphorylation via trans‐systemic signaling axes like TLR4/NF‐κB [178]. Inflammatory factors released by glial and peripheral immune cells form a self‐reinforcing vicious cycle that directly damages neurons and inhibits normal neurotransmitter function, such as that of ACh, with a decline in its level being directly associated with cognitive decline, and drives abnormal tau phosphorylation. Furthermore, phosphorylated tau can, in turn, activate the NLRP3 inflammasome within microglia, exacerbating the release of mediators such as IL‐1β [179].

The disruption of astrocytic activity contributes significantly to disease progression, dysregulating glutamate metabolism, leading to excitotoxicity and the exacerbation of neuronal damage and damaging BBB integrity, increasing its permeability [180]. This enables the peripheral inflammatory cells and mediators to enter the brain parenchyma more easily, further worsening the inflammatory environment [181]. Simultaneously, released complement proteins such as C1q and C3 mediate harmful synaptic pruning [182].

In AD, inflammatory signaling hinges principally on pattern recognition receptors (PRRs), with TLRs representing pivotal mediators. As damage‐associated molecular patterns (DAMPs), Aβ oligomers and tau are detected by TLRs, stimulating microglia and astrocytes to secrete inflammatory mediators [183]. This process both amplifies inflammatory factor release and induces tau hyperphosphorylation, ultimately aggravating neuroinflammation [184]. Further, microglial surface TLR4/MyD88 activation results in the initiation of downstream NF‐κB/MAPK cascades by Aβ, potently stimulating proinflammatory factor synthesis. Conversely, TLR4 engagement may suppress TREM2‐mediated Aβ clearance, inducing “inflammatory clearance imbalance” [185]. As a pivotal transcriptional regulator, NF‐κB undergoes activation and broadly induces inflammatory gene expression, propagating the inflammatory storm and accelerating neuronal death [186]. Additionally, the pathogenesis of AD involves an intrinsic imbalance in cellular homeostasis. Aβ and tau evoke oxidative burden and mitochondrial impairment, worsening neuronal injury and provoking inflammatory responses [187, 188]. Impairment of the essential protein clearance pathways, autophagy, and the ubiquitin–proteasome system (UPS), concurrently occurs, fostering the aggregation of pathogenic proteins including Aβ and tau, that initiates a self‐reinforcing loop that exacerbates inflammatory and neurodegenerative processes [189, 190]. In conclusion, the core pathological manifestations of AD have several distinct characteristics. These features mainly consist of: Aβ‐driven senile plaques from aberrant accumulation, neurofibrillary tangles composed of hyperphosphorylated tau protein, significant neuronal loss, and extensive synaptic damage. It should be emphasized that these pathological changes are not isolated phenomena and are rooted in an extremely complex neuro‐immune inflammatory network.

The significance of studying neuro‐immune crosstalk in AD is widely recognized; however, it still faces several challenges. Whether the peripheral immune system in AD is overactivated, under‐responsive, or functionally exhausted remains unclear. Further, translating findings from rodent models to humans is difficult, primarily due to a lack of effective animal models of aging, neglect of sex differences, and the inability to reflect multiple comorbidities specific to human aging, which leads to the clinical failure of many immune‐modulating strategies that are effective in animal models. Additionally, current imaging and fluid biomarker analyses lack cell type specificity and cannot capture the spatiotemporal dynamics of central and peripheral immune interactions in real time. These issues lead to imperfect clinical trial designs due to uncertainty of intervention time windows, inconsistent endpoint indicators, and high patient heterogeneity, ultimately resulting in repeated failures of anti‐inflammatory or immune‐modulating therapies; however, the exact reasons for these failures remain unclear. In summary, despite the accumulation of a large amount of immune data, the key immune interactions determining neuronal fate remain unclear, making it difficult to achieve precise and effective immune intervention in the population.

##### 3.1.1.2 Parkinson's Disease

PD, synonymous with “tremor paralysis,” is a degenerative neurological condition. Its hallmark pathology involves progressive dopaminergic neuronal depletion within the substantia nigra pars compacta, which culminates in striatal dopamine (DA) decline and Lewy body formation (7), resulting in direct damage to the pathway that regulates motor function, causing a significant decrease in DA levels in the striatum [191].

Microglial activation is mediated by the surface expression of various PRRs, including TLRs and NOD‐like receptors (NLRs), that detect pathogen‐associated molecular patterns (PAMPs) and DAMPs. The pathogenic α‐syn fibers act as key DAMPs that activate the NLRP3 inflammasome in microglia through the CD36 receptor, triggering IL‐1β secretion and promoting the formation of an inflammatory microenvironment [192]. Furthermore, cytokines and chemokines such as IFN‐γ, IL‐1β, CCL2, and monocyte chemoattractant protein‐1 (MCP‐1), released after neuronal damage, can also activate microglia, and activated microglia further secrete cytokines and chemokines such as TNF‐α and IL‐6, exacerbating the inflammatory response [193]. Moreover, neurotransmitters also regulate microglial activation. For example, DA inhibits the activation and reduces the release of inflammatory mediators [194] and NE activates microglia and promotes inflammatory response [195]. T cell‐secreted cytokines can also activate microglia, further aggravating inflammation [196].

Astrocytes are widely activated in patients with PD. Postinjury neuron‐derived ATP/glutamate serves as an astrocyte‐activating signal, and microglial inflammatory mediators also similarly stimulate these cells. Activated astrocytes secrete NO, prostaglandin E2 (PGE2), and neurotoxins, causing immediate harm to dopaminergic neurons and intensifying inflammation and neuronal injury through the activation of other immune cells [197]. α‐Syn can interact with the lymphocyte activation gene 3 receptor located on the surface of astrocytes, triggering the release of CXCL10 and drawing peripheral Th17 cells into the CNS, thereby further exacerbating immune‐mediated neuronal impairment [198, 199]. Astrocytes communicate with neurons through direct channels to relay neuronal injury signals. CCL2/MCP‐1 subsequently guides their migration toward impaired DA neurons, worsening inflammation and neuronal damage. Furthermore, inflammatory mediators including TNF‐α, IL‐1β, and IL‐6 activate astrocytes, stimulating the release of additional inflammatory factors and neurotoxic compounds [200]. Mitochondrial DNA (mtDNA) released from damaged dopaminergic neurons in the substantia nigra functions as a DAMP, activating the cGAS–STING axis to potentiate IFN synthesis and amplify neuroinflammation [201]. After the NF‐κB signaling pathway is activated, it leads to the activation of microglia and astrocytes, releasing more inflammatory mediators and neurotoxic substances. Its activation can be triggered by PRRs on the surface of microglia and astrocytes, as well as cytokines and chemokines released by neuronal damage. And the cytokines and chemokines secreted by these cells will further activate this pathway [202]. The MAPK signaling pathway, including extracellular signal‐regulated kinase (ERK), p38 MAPK, and c‐Jun N‐terminal kinase (JNK), is also activated, recognizing PAMPs and DAMPs through PRRs and the cytokines and chemokines released by neurons, thereby activating microglia and astrocytes, and promoting the release of inflammatory mediators [203, 204]. Additionally, autophagy and UPS dysfunction leads to a vicious cycle of neuroinflammation. The blocked clearance of α‐syn aggregates caused by defects in autophagy or the UPS, results in continual microglial activation, leading to cytokine release and persistent oxidative stress [205, 206]. These inflammatory mediators further exacerbate α‐syn accumulation by impairing lysosomal activity and enhancing the mammalian target of rapamycin [207].

In research on neuro‐immune crosstalk in PD, the core controversy focuses on the nature of microglial cell activation, as the balance between neuroprotective clearance of α‐syn and neurotoxic inflammatory response is complicated, and the role of peripheral immunity, including the pathological contribution of T cell infiltration into the midbrain and the discrepancy in the amount of anti‐inflammatory regulation. The primary factor complicating research efforts is the complexity of multisystem interactions, including the difficulty in modeling the dynamic coupling of α‐syn pathological spread, mitochondrial dysfunction, and immune metabolic reprogramming, as well as existing technical bottlenecks, such as the lack of methods for real‐time monitoring of the dynamic changes in BBB permeability, and the inability of animal models to replicate the chronic neuroinflammatory process in the human multigene background. The key shortcomings lie in the translational bottleneck: peripheral blood inflammatory markers such as IL‐1β and TNF‐α have weak correlation with the central immune state, and the clinical application of cerebrospinal fluid (CSF) sampling or PET imaging techniques is also limited. Further, treatment strategies have limitations: targeting NLRP3 or LRRK2 immunomodulatory therapies are only effective for specific subgroups, and lack predictive biomarkers; interdisciplinary integration is insufficient and organoid models have not fully simulated the neural–immune–glial cell metabolic network. Moreover, the predictive accuracy of computational models for the inflammatory spread in the substantia nigra–striatum pathway needs improvement.

In summary, the pathology of PD is a complex process driven by the interaction and cooperation of microglia, astrocytes, and multiple signaling pathways. Microglia are activated by damage signals such as α‐syn through PRRs, releasing a large amount of inflammatory factors to exacerbate neuroinflammation. Their activation is regulated by multiple factors such as neurotransmitters. Astrocytes are activated by neuronal injury signals and inflammatory mediators from microglia, releasing toxic substances, and recruiting peripheral immune cells to aggravate neuronal damage, exerting an inflammatory amplification effect on microglia. The signaling pathways such as cGAS–STING, NF‐κB, and MAPK are not only the key drivers of the activation of these glial cells but also form a vicious cycle with the dysfunction of autophagy/UPS, promoting the progressive degeneration of dopaminergic neurons. This multilevel and networked pathological mechanism suggests that future treatments for PD must overcome the limitations of single‐target approaches. By coordinating the regulation of the activation state of glial cells, intervening in key signaling pathways, restoring protein homeostasis, and other multidimensional strategies, it is possible to break the vicious cycle of inflammation and neuronal damage, thus providing new ideas for delaying or even blocking disease progression.

##### 3.1.1.3 Huntington's Disease

HD is an autosomal dominant neurodegenerative disorder caused by an abnormal expansion of CAG trinucleotide repeats in the huntingtin (HTT) gene and is characterized pathologically by the progressive degeneration and death of striatal neurons [208, 209]. Neuro‐immune crosstalk plays a critical role in HD pathogenesis.

The microglia in the HD brain are activated, and the activation intensity correlates with disease progression. Dying nerve cells discharge DAMPs such as ATP and high mobility group box 1 (HMGB1), that stimulate NF‐κB through microglial P2×7 receptors and TLR4 pathways, promoting continuous production of proinflammatory cytokines (IL‐1β, TNF‐α, IL‐6), inducing neuroinflammation and intensifying the neural damage [210]. Oligomers of mutant HTT (mHTT) bind to microglial surfaces via unknown receptors, inducing polarization from the quiescent M0 to the neurotoxic proinflammatory M1 phenotype. M1 microglia secrete MMP‐9 to disrupt the BBB [211], while impaired phagocytosis prevents the clearance of mHTT inclusions, with toxic proteins being transmitted via exosomes [212]. M1 polarization also reduces anti‐inflammatory IL‐10 secretion and disrupts immune homeostasis. Similarly, astrocytes are activated in HD, releasing inflammatory mediators that synergize with microglia to exacerbate neuroinflammation [213]. These cells maintain neural homeostasis by regulating neurotransmitter metabolism (e.g., glutamate uptake/release); however, functional abnormalities in HD cause glutamate dysmetabolism, triggering excitotoxicity and worsening neuronal damage [214]. Increased BBB permeability in HD allows peripheral immune cells (T lymphocytes, macrophages) to invade the brain: Th1 cells release IFN‐γ to activate microglia and induce MHC‐I expression in astrocytes, potentially triggering cytotoxic attacks on degenerating neurons [215]; Treg cells, compromised by reduced numbers or functional defects (e.g., mHTT‐inhibited Foxp3 expression), lose their anti‐inflammatory capacity [216]. Aberrant mHTT expression in peripheral monocytes overactivates the IL‐6/STAT3 pathway, promoting differentiation into the proinflammatory M1 phenotype, which then invade the CNS through the damaged BBB and release inflammatory mediators [217]. Additionally, mHTT suppresses PGC‐1α‐mediated mitochondrial biogenesis, inducing ROS accumulation and activating the NADPH oxidase 2 complex in microglia, thus driving an NF‐κB‐dependent inflammatory response [218]. Simultaneously, mHTT disrupts the ubiquitination and degradation of the STING protein, resulting in sustained IFN signaling activation [219]. In HD, mHTT‐induced oxidative stress and mitochondrial dysfunction synergize with impairments in autophagy and the UPS [220], collectively promoting aberrant mHTT accumulation [221]. These pathological alterations not only directly trigger neuronal damage and death but also activate microglia and astrocytes to release inflammatory mediators. This cascade establishes a chronic neuroinflammatory cycle that accelerates disease progression. In the GBA, HD mouse models show dysbiosis (imbalanced Firmicutes/Bacteroidetes ratio) and reduced SCFA‐producing bacteria, increasing intestinal mucosal permeability and allowing LPS translocation [222]. Circulating LPS activates peripheral monocytes to release IL‐6, which indirectly promotes microglial activation [223]. Additionally, IL‐1β secreted by intestinal lamina propria immune cells activates vagal afferents, transmitting inflammatory signals to the nucleus of the solitary tract to “preactivate” brain microglia, enhancing their sensitivity to mHTT stimulation [224].

Research on neuro‐immune crosstalk in HD remains controversial. For instance, it remains unclear whether peripheral and central inflammation drive disease progression in the early stages of the disease or are passive outcomes of neuronal damage. The key difficulty lies in the lack of tools for the real‐time tracking of immune cell dynamics in living organisms, which leads to inconsistent correlations between inflammatory markers in the CSF and peripheral blood and symptom severity in clinical cohorts, making it impossible to determine the optimal intervention time window. Additionally, due to differences in CAG repeat length, genetic background, and so on in mouse models, the patterns of gliosis are different, and young mice lack obvious inflammation, making it difficult to simulate early immune activation in humans, limiting the translation of mechanistic research. Large animal models have potential, but are limited by high costs, long cycles, and ethical restrictions. Moreover, the causal relationship between the peripheral immune status and central immune cells is unclear, and there is a lack of standardized longitudinal sampling procedures and antigen response tracking technologies, making it difficult to clarify the interaction mechanism. From a clinical perspective, HD progresses slowly and exhibits large individual variation. Traditional scales find it difficult to quantify the effects of immune interventions and lack rapid assessment alternatives, resulting in long follow‐up periods for anti‐inflammatory trials. Although a large amount of immune data has been accumulated, the key immune interactions that determine the fate of striatal neurons have not been elucidated, and precise and effective immune interventions have not been achieved in patients.

In summary, the pathogenesis and progression of HD are closely linked to dysregulated neuro‐immune communication. This multifaceted mechanism involves the synergistic involvement of central glial elements, peripheral immune populations, and diverse molecular signals, which perpetuate a self‐reinforcing cycle that aggravates neuronal injury. Aberrantly activated microglia and astrocytes compromise BBB integrity, facilitating peripheral immune infiltration, and mHTT‐driven chronic inflammatory signaling via multiple routes contributes not only to direct striatal neurodegeneration, but also propagates neuroinflammation through disrupted immune homeostasis, thereby accelerating disease advancement. This suggests that in‐depth research on HD needs to overcome the limitations of single cells or pathways and start from the overall perspective of the neuro‐immune network to explore comprehensive strategies for targeting the regulation of glial cell functions, restoring immune balance, and blocking the inflammatory cascade reaction. As such, it will contribute significantly to the development of HD treatments that mitigate disease progression, in addition to furnishing a foundational reference for understanding the complex nexus of neurological and immunological processes in neurodegenerative diseases.

##### 3.1.1.4 Amyotrophic Lateral Sclerosis

ALS is a progressive chronic neurological disorder [225]. Its core pathological features include the degeneration of axons and atrophy of cell bodies in the cerebral cortex, brainstem, and anterior horn motor neurons of the spinal cord, ultimately leading to dysregulated nerve impulse conduction and functional loss [226].

Abnormal protein aggregation is a significant pathological marker in neurons, with manifestations including mutated superoxide dismutase 1 and abnormal localization of TAR DNA‐binding protein 43 (TDP‐43) [227]. TDP‐43 migrates from the nucleus to the cytoplasm to form ubiquitin‐positive inclusions, interfering with RNA metabolism and protein homeostasis, activating the pERK/eIF2α pathway to trigger the unfolded protein response, and simultaneously inhibiting X‐box binding protein 1‐mediated adaptive responses, ultimately triggering CHOP‐dependent apoptosis [228]. Glial cells play important roles in the complex pathophysiology of ALS. Microglia, as the central immune sentinels, release inflammatory factors such as IL‐1β and TNF‐α after activation, directly causing neuronal damage [229]. Impairment of microglial phagocytic function leads to abnormal protein accumulation and persistent inflammation [230]. Moreover, dysregulation of the gut microbiota has also been described to be involved in ALS progression. For example, a decrease in the abundance of Firmicutes leads to a decrease in SCFA levels, which inhibits the HDAC3 activity of microglia and hinders their polarization toward the anti‐inflammatory M2 phenotype [231].

Astrocytes undergo reactive transformation, releasing neurotoxic molecules such as NO, ROS, and PGE2, and reduce neuronal glutamate uptake capacity to trigger excitotoxicity [232]. Additionally, the chitinase‐3‐like protein 1 secreted by astrocytes can bind to the IL‐13Rα2 receptor of motor neurons, activating the JAK2/STAT3 pathway and exacerbating excitatory injury [233].

A pronounced influx of immune cells into the spinal cord and CSF is also observed in ALS, where infiltrating T cells contribute to disease pathology by secreting inflammatory factors like IFN‐γ and TNF‐α, thereby intensifying the local inflammatory milieu. B cells may produce autoantibodies against motor neurons [234], whereas macrophages participate in the deterioration of the microenvironment through phagocytosis and the release of inflammatory mediators [235]. The abnormal activation of the cytokine and chemokine network, such as elevated levels of TNF‐α, IL‐6, and CCL2/MCP‐1, can not only activate microglia and astrocytes to promote inflammatory responses, but also directly damage neurons [236]. Elevated CCL2/MCP‐1 levels can recruit macrophages and T‐cells to lesion sites, exacerbating the inflammatory response. Oxidative stress and mitochondrial dysfunction are key injury mechanisms in ALS pathophysiology. Abnormal energy metabolism in motor neurons leads to excessive ROS production, directly damaging proteins, lipids, and DNA structures, and inducing cell death [237]. Simultaneously, ROS, as potent activators, can trigger the inflammatory transformation of microglia and astrocytes, forming a positive feedback loop for neuroinflammation [238]. This oxidative damage is closely associated with an imbalance in protein homeostasis, and dysfunction of autophagy and the UPS leads to the continuous accumulation of abnormal proteins, such as SOD1 and TDP‐43, which directly damage neurons and further activate glial cells to release inflammatory mediators [239]. Moreover, oxidative stress, abnormal protein deposition, and inflammatory factors jointly constitute the core activating factors of the NF‐κB and MAPK signaling pathways including the ERK, p38 MAPK, and JNK pathways [240]. The activated NF‐κB and MAPK pathways form a cascade of amplification effects, strongly driving glial cell activation and the release of inflammatory mediators and regulating the migration and activation of immune cells to the lesion site, thereby deeply participating in the spread of neuroinflammation and motor neuron damage [241].

The core controversy in research on neuro‐immune crosstalk in ALS is not whether immune responses are involved, but rather the timing and extent of their involvement. Whether peripheral immunity causes the early loss of protective function or subsequent excessive activation exacerbates nerve damage remains unclear. The key difficulty lies in the lack of a stable immune dynamic timeline that can be reproducibly observed in patients with different genetic mutations and progression rates, resulting in unstable correlations between peripheral inflammatory markers and clinical severity, and even completely opposite immune characteristic profiles in the same patient over a short period of time. Methodologically, the traditional human superoxide dismutase transgenic mouse model, owing to its accelerated progression and single mutation background, cannot simulate the multigene participation and multisystem aging process in humans, reducing the reliability of inferring the “peripheral to central” immune causal chain. TSPO–PET imaging can detect glial cell activation, but the signal plateaus early, making it impossible to distinguish between functional polarization states and synchronously monitor peripheral immune dynamics, limiting the interpretation of the immune cascade initiation process. Furthermore, the immune state of ALS is highly individualized. For example, IL‐6 blockers can alleviate inflammation in patients with elevated inflammation and enhance inflammation in those with low inflammation. Targeted mutation gene therapy may trigger unpredictable neuroinflammation, forcing clinical trials to repeatedly explore intervention time, duration, and dosage, but lacks a dynamic stratified diagnostic intervention system that integrates BBB permeability, peripheral immune cell function, and central glial activity. Although local immune abnormalities can be identified, no dynamic monitoring and stratification models covering the entire process or guiding precise immune interventions have been established.

Overall, the neuro‐immune axis in ALS begins with a stress response triggered by gene mutations and protein abnormalities in neurons, which causes microglial activation and BBB disruption, allowing peripheral immune cells to infiltrate and trigger autoimmune responses. Abnormal signal transduction involving cytokine networks, NF‐κB, and MAPK pathways results in a vicious cycle, leading to progressive loss of motor function. Based on this framework, future treatments will need to integrate multiple approaches such as anti‐inflammatory strategies, immune regulation, and genetic intervention.

##### 3.1.1.5 Multiple Sclerosis

MS is a complex disease with an autoimmune response as the core initiating mechanism. Neurodegenerative features gradually emerge with disease progression. MS is characterized by multiple demyelinating plaques, mainly involving the white matter of the brain, spinal cord, and optic nerve regions, causing myelin destruction while axons are relatively preserved, eventually forming glial scars [242].

The initial phase of MS features a vigorous immune reaction involving substantial immune cell invasion into the CNS, leading to swift demyelination and BBB disruption, which are observed as new enhancing lesions on MRI [243]. As the disease progresses to the chronic phase, the inflammatory response weakens and demyelinating plaques gradually evolve into glial scars, that severely hinder axonal and myelin regeneration, complicating the recovery of neurological function. Thus, treatment strategies need to account for the dynamic changes in immune responses between the acute and chronic phases; for instance, suppression of the immune inflammatory response should be the focus in the acute phase, whereas in the chronic phase, promoting neural repair is necessary [244].

Microglia become activated by specific immune signals (e.g., ATP, TNF‐α) and subsequently secrete various cytokines (e.g., IL‐1β) and chemokines (e.g., CCL2) to orchestrate neuro‐immune responses. They can regulate neuronal survival and repair, providing protection by releasing neurotrophic factors in the early stages of demyelination but may also be overly activated in persistent inflammation, exacerbating neuronal damage [245]. Astrocytes participate in MS pathophysiology by proliferating and forming glial scars to isolate the damaged area; however, excessive scarring can inhibit the regenerative capacity of nerve fibers. Signal pathways involve multiple mechanisms: the activation of microglia and astrocytes depends on pathways such as TLR, NF‐κB, and MAPK, which can recognize self‐antigens or DAMPs and trigger the release of inflammatory mediators [246]. The activation of cytokine receptors regulates immune activity through JAK/STAT signaling, whereas chemokine receptors activate pathways such as phospholipase C and protein kinase C (PKC) to control the migration and chemotaxis of immune cells. In T cells, the binding of TCR to MHC–antigen peptide complexes initiates activation, and CD28‐B7 costimulation further promotes proliferation. Upon activation, T cells release inflammatory cytokines like IFN‐γ and TNF‐α, that augment the inflammatory response through autocrine and paracrine signaling. B cells are activated through BCR–antigen binding and CD40–CD40L costimulation and differentiate under the influence of cytokines to produce autoantibodies [247]. B cell‐activating factor signaling drives the differentiation of follicular helper T cells, promoting the formation of germinal centers and the production of antibodies, such as antiaquaporin‐4 antibodies.

From a core mechanistic perspective, autoimmunity is the key driver of MS pathogenesis. CD4^+^ T cells cross the BBB and enter the CNS, recognizing myelin antigens and releasing cytokines to recruit macrophages to phagocytose myelin, thereby accelerating the formation of demyelinating plaques [248]. CD8^+^ T cells recognize antigens on the surface of oligodendrocytes via MHC‐I and release granzyme B to induce apoptosis. Th17 cells secrete IL‐17, activating the IL‐17R/Act1/TRAF6 axis in astrocytes, promoting the release of CXCL1 and recruiting neutrophils for infiltration [249]. Throughout this process, a complex interaction network is formed between immune and glial cells.

Currently, research on neuro‐immune crosstalk in MS faces several challenges. For instance, the acquisition of lesion tissue samples is limited, clinical biopsies are rare, and autopsy tissues mostly show terminal stage changes, making it difficult to directly study early immune events. Additionally, clinical phenotypes are highly heterogeneous and different subtypes can transform within the same disease course, resulting in inconsistent associations between peripheral inflammatory markers and the progression of neurological dysfunction in different patient groups. Moreover, long‐term reliance on experimental autoimmune encephalomyelitis (EAE) mouse models, owing to significant differences in dominant T cell subsets, disease progression, and human MS, reduces the reliability of inferring a causal relationship between peripheral and central immunity. Furthermore, the functions of microglial cell subpopulations are controversial, with no consensus on classification, and the dual nature of proinflammatory and protective properties remains unclear. Moreover, the contribution of B cells, CD8^+^ T cells, and the Epstein–Barr virus is established, and existing treatments can control relapses but cannot prevent progressive neurodegeneration, suggesting that the central retention mechanism may be independent of peripheral immune regulation. Further, the lack of unified multiomics research standards leads to differences in the interpretation of the functions of the same immune cell populations, and imaging and fluid biomarkers cannot simultaneously monitor BBB permeability, peripheral immune dynamics, and central glial activation. These problems force clinical trials to repeatedly adjust intervention timing and dosage, making it difficult to establish an intervention monitoring system that guides precise treatment.

Therefore, MS is considered an “immune‐mediated neurodegenerative disease.” Autoimmune responses initiate and cause disease relapse, and targeted immunotherapy can control acute immune activity. Neurodegeneration is the root cause of disability progression, especially in the chronic stage where it continues to deteriorate independently of inflammation. Thus, future treatments must be dual‐pronged, specifically inhibiting pathogenic immune responses in the acute phase and blocking neurodegenerative pathways, such as targeting glial scars, enhancing mitochondrial function, and promoting myelin regeneration in the chronic phase, to achieve true disease treatment.

In summary, current research on neurodegenerative diseases has achieved breakthroughs in several aspects. Disease‐modifying therapies (DMTs) such as lecanemab have demonstrated efficacy in clearing Aβ plaques and delaying progression in early‐stage AD, with data showing significant symptom stabilization or improvement in early patients. Drug repurposing strategies (e.g., letrozole plus irinotecan combination) can reverse memory decline and pathological damage in AD animal models by targeting aberrant gene networks in neurons and glial cells [250]. Blood‐based biomarkers (e.g., pTau217) and artificial intelligence (AI) screening technologies have enhanced early diagnostic feasibility, enabling the intervention window to shift to the preclinical stage. Single‐cell technologies have revealed disease‐specific cellular heterogeneity, such as the identification of the microglial subpopulations MG2 (impaired electron transport chain) and MG4 (IFN response) as precise therapeutic targets in ALS [251]. However, significant limitations and challenges remain. Translational bottlenecks remain prominent due to discrepancies between animal models and human pathology, exemplified by senolytic agents such as navitoclax, which demonstrated safety and efficacy in mice yet induced nonselective neurotoxicity in human neuronal models [252]. Mechanistic understanding is also incomplete, as illustrated by the role of the glymphatic system in AD; while validated in animals, human verification is hindered by the lack of noninvasive detection tools, and its causal relationship with Aβ/tau accumulation remains unconfirmed [253]. Furthermore, high treatment costs limit widespread accessibility and current strategies inadequately address disease complexity. To address these issues, clinical translation efforts should prioritize human cell models (e.g., neuron–glia coculture systems) for drug safety validation to mitigate species‐disparity risks [252], while early diagnosis and intervention require the integration of blood biomarkers with community screening networks to shift AD treatment to the preclinical stage, explore preventive DMT applications, and advance noninvasive monitoring tools (e.g., dynamic MRI assessment of the glymphatic system) for optimized efficacy tracking [253].

#### 3.1.2 Chronic Pain

Chronic pain disorders are characterized by persistent inflammatory sensitization and amplified nociceptive signaling cascades, manifesting as inflammation mediator‐driven hyperalgesia and maladaptive neural circuit remodeling. The core mechanism involves bidirectional dysregulation of peripheral and central neuro‐immune regulatory networks.

##### 3.1.2.1 Fibromyalgia

FM is characterized by widespread chronic pain, fatigue, and sleep disorders [254]. The core pathology involves central sensitization and the interaction between the nervous and immune systems is a key driver of this process [255]. Elevated proinflammatory factors such as IL‐6, TNF‐α, and IL‐1β in the serum and CSF of patients directly stimulate sensory neurons and induce the release of neurotrophic factor (NGF), enhancing the sensitivity and receptor expression of nerve endings [256, 257]. Moreover, immune cells establish direct “communication channels” with the nervous system: for instance, chemokines secreted by macrophages and T cells, such as CXCL13, can promote the formation of similar “immune synapses” between sensory nerves and immune cells, continuously transmitting inflammatory information through the CX3CL1–CX3CR1 signaling axis, persistently maintaining the pain state [258]. Abnormal excitation of the sympathetic nerve constitutes a crucial positive feedback loop, which stimulates mast cells to release mediators such as histamine (HA) and trypsin and promotes the secretion of neuropeptides such as SP and CGRP, further exacerbating local neurogenic inflammation and immune activation [259].

HPA axis dysfunction is another important dimension of neuro‐immune imbalance in FM. Insufficient cortisol secretion weakens the body's anti‐inflammatory capacity, promoting the activation of proinflammatory Th17 cells and the release of IL‐17 and relieving microglial inhibition, jointly exacerbating the inflammatory environment in the CNS [260]. Mitochondrial dysfunction is also closely associated with FM, leading to excessive ROS production and triggering oxidative stress [261], which directly damage tissues and trigger systemic inflammation, amplifying pain and fatigue. Importantly, mitochondrial damage products (such as mtDNA, ROS) are themselves potent DAMPs, which can overactivate the innate immune system (such as the NLRP3 inflammasome), leading to uncontrolled inflammatory responses and forming a “mitochondrial–inflammation axis,” further worsening symptoms [262].

In recent years, the gut microbiota has emerged as a crucial “third‐party participant” in the neural–immune crosstalk of FM [263]. Studies have shown that transplanting the gut microbiota of patients with FM into germ‐free mice can successfully induce pain sensitivity and depression‐like behaviors similar to those in patients with FM, accompanied by peripheral immune activation and spinal microglial cell activation [264], providing evidence of the role of gut microbiota dysbiosis in triggering and maintaining the pain pathology in FM, thus offering a compelling scientific basis for therapeutic strategies targeting the gut microbiome.

Currently, the core controversy in research on the neuro‐immune crosstalk in FM lies in the causal status of neuroinflammation, whether it is the primary driving factor or a secondary manifestation of chronic stress. This controversy stems from inconsistent research results, the unstable correlation between peripheral inflammatory factors and pain and disability levels, the lack of validation of central microglial cell activation signals in large sample studies and their influence by genetic polymorphisms, and the absence of a clear diagnostic threshold for “low‐grade neurogenic inflammation.” Methodologically, the small sample size and high heterogeneity of research designs have made it difficult to replicate the results across studies. The temporal and spatial correspondence between CSF and plasma immune markers, brain imaging, and peripheral blood immune cell functions lacks unified collection and analysis standards. Additionally, there are contradictory reports on the function of the HPA axis and vagal nerve tension, with an unclear direction of change that hinders clarification of the causal relationship between stress, immunity, and pain. Owing to the lack of subgroup classification based on immune biomarkers, clinical trials rely solely on clinical symptoms for patient selection, intervention timing, and efficacy assessment, making it impossible to identify the population that benefits from the interventions, and difficult to verify whether the intervention strategy acts on the core pathological mechanism.

In conclusion, the neural–immune crosstalk in FM presents as a multilevel, mutually reinforcing complex network involving proinflammatory factors, abnormal direct communication between neural and immune cells, HPA axis dysregulation, activation of the mitochondrial–inflammation axis, and a disordered GBA. These mechanisms collectively drive central sensitization and systemic symptoms. Future research is urgently needed to more deeply analyze the dynamic interaction relationships among these pathways, especially how the gut microbiota precisely regulates the neural–immune axis. At the translational medicine level, developing precise therapies based on these mechanisms holds great promise, such as targeting specific proinflammatory pathways (IL‐6, IL‐17, and NLRP3), regulating sympathetic nerve activity, repairing mitochondrial function, regulating HPA axis reactivity, and reshaping the overall neural–immune homeostasis of the body by intervening in the gut microbiota and its metabolites, thereby providing more effective treatment options for patients with FM.

##### 3.1.2.2 Migraine

Migraine is a common chronic neurological disorder classified as a primary headache. It is characterized by recurrent, often one‐sided, moderate‐to‐severe throbbing pain, frequently accompanied by nausea and vomiting. Headaches can be exacerbated by light, sound, or routine activities, whereas rest in a quiet environment often provides relief. The development of migraine is closely linked to a dynamic imbalance in the neuro‐immune system [265], which manifests as neural activity triggering an immune response, which in turn acts back on the nervous system, forming a pathological “neuro‐immune crosstalk” loop.

Migraine attacks often begin with the activation of the trigeminal nerve, releasing neuropeptides such as CGRP and SP [266]. CGRP dilates the meningeal blood vessels and increases vascular permeability, causing plasma protein leakage and sterile inflammation. Simultaneously, CGRP promotes the accumulation of immune cells, including mast cells and macrophages, to the meninges and induces them to release proinflammatory factors such as TNF‐α and IL‐6, further intensifying inflammation [267]. Furthermore, excessive neuronal excitation in the trigeminal nuclei (e.g., trigeminal nucleus caudalis) can cause central sensitization. The CNS regulates peripheral immune cell function via the autonomic nervous system (e.g., sympathetic nerves) [268]. Neuropeptides such as CGRP can also disrupt the TJs of the BBB, making it easier for peripheral immune cells such as T cells and monocytes to enter the CNS. Peripheral immune activation triggered by pathogen infection, stress, or genetic factors causes pain sensitization. Macrophages and DCs gather around the meninges or cerebral blood vessels, releasing proinflammatory factors like IL‐1β, IL‐6, and TNF‐α, which directly stimulate the trigeminal nerve endings, lowering the pain threshold and triggering migraine attacks [269, 270]. An imbalance in the gut microbiota can also activate intestinal mucosal immunity. The resulting inflammatory factors (like LPS) travel via the bloodstream to the CNS, activating microglia and enhancing pain signal processing in the trigeminal nuclei. This explains the gastrointestinal symptoms experienced by some patients with migraine [271]. Additionally, peripherally activated T‐cells can cross the BBB into the CNS and interact with neurons or glial cells. IFN‐γ released by Th1 cells heightens neuronal sensitivity to pain signals, while reduced numbers or impaired function of Treg cells may weaken inflammation suppression, promoting the development of chronic migraines [272].

Current research on neural–immune crosstalk in migraines faces several challenges. For instance, the associations between peripheral and central inflammatory markers and the frequency and intensity of attacks are often inconsistent across different studies, and there is a lack of consistent cross‐regional and cross‐ethnic thresholds and time points, making it difficult to determine whether immune abnormalities are triggering factors or secondary reactions to attacks. Moreover, existing studies generally have small sample sizes and high protocol heterogeneity. Single‐cell and multiomics data mainly come from European populations and lack validation in Asian and Latin American populations, limiting the generalizability of the conclusions. Additionally, most animal models and in vitro experiments simulate acute attacks and are unable to reproduce the long‐term, low‐level inflammatory state and comorbid environment of chronic migraine in humans. At the same time, although studies on the brain–gut axis and activation of meningeal mast cells suggest a tripartite interaction between the microbiota, immunity, and the nervous system, there is a lack of standardized sampling procedures and longitudinal follow‐up data, making it difficult to determine the effectiveness of potential intervention targets and the evaluation methods. Therefore, comprehensive studies with large sample sizes, multiple centers, covering different migraine subtypes and populations of different races, are urgently needed in order to establish an immune characteristic stratification system that can be used to guide treatment.

In summary, migraine results from a neuro‐immune dynamic imbalance. Neural signals initiate immune inflammation via neuropeptide release, whereas immune activation feeds back on the central and peripheral nervous systems via cytokines and inflammatory signals. This bidirectional interaction constitutes the core pathological mechanism of migraines.

### 3.2 Immune Dysregulation‐Driven Disorders

Dysregulation of the neuro‐immune system not only drives local neuroinflammation but also serves as the core pathophysiological basis for a wide range of disorders, from classic autoimmune diseases targeting neural tissues to complex neuropsychiatric conditions. Focusing on autoimmune and neuropsychiatric diseases, we analyze how distinct patterns of immune dysregulation manifest within the neuro‐immune axis, identifying key pathways driving core pathological processes, and explore potential therapeutic directions.

#### 3.2.1 | Autoimmune Diseases

Autoimmune diseases represent a category of pathological conditions characterized by an aberrant immune response that targets tissues and organs. Neuro‐immune interactions play a pivotal regulatory role in the pathogenesis, progression, and clinical manifestations of diverse autoimmune disorders, such as RA, SLE, and psoriasis.

##### 3.2.1.1 Rheumatoid Arthritis

RA is an autoimmune disease characterized by synovitis as its pathological basis, which may eventually lead to joint deformities [273]. Its etiology is still being explored, but studies have shown that it may be closely related to factors such as autoimmunity, genetics, and microbial infections [274].

The core of RA involves an imbalance in the autoimmune system. Chronic synovitis results in progressive joint damage and systemic symptoms [275]. In this immune‐centered pathological process, neuro‐immune interactions, as key additional regulatory layers, bidirectionally amplify inflammation and damage [276]. In an immune‐activated joint environment, sensory nerves such as C fibers release neuropeptides such as SP. By binding to NK1 receptors on synovial macrophages and T cells, they directly stimulate the production of key proinflammatory factors such as IL‐1β, TNF‐α, and IL‐17, exacerbating local inflammation [277]. The role of the SNS is contradictory: in theory, its neurotransmitter NE can inhibit proinflammatory M1 macrophages through β_2_‐AR; however, sympathetic nerve dysfunction commonly seen in RA joints, such as neurotransmitter depletion, significantly weakens this inherent anti‐inflammatory brake [278]. Furthermore, NE exacerbates Th1/Th2 imbalance through β_2_‐AR, promotes IFN‐γ, inhibits IL‐4, and strongly drives neutrophil infiltration and oxidative bursts through β_1_‐AR, synergizing with the immune system to promote synovial inflammation and damage [279]. At the systemic level, long‐term dysfunction of the HPA axis weakens the ability of endogenous GCs to suppress immune storms, thus providing a background for uncontrolled neuro‐immune‐mediated inflammation [280]. Meanwhile, the immune system also profoundly shapes neural functions in reverse: local inflammatory factors continuously sensitize pain‐sensing neurons, while circulating proinflammatory factors such as TNF‐α and IL‐6 can penetrate or signal to the CNS, directly inducing disabling systemic symptoms like fatigue and depression, forming a vicious circle [281].

Currently, there are still controversies and deficiencies in the research on neuro‐immune crosstalk in RA. The causal sequence between peripheral inflammatory signals and central pain sensitization has not been clarified. The correlations between the levels of cytokines such as IL‐6 and TNF‐α and pain scores in different cohorts vary, and there is a lack of a reproducible time window threshold. Moreover, animal models (CIA mice) and human samples showed opposite patterns in circadian rhythms, microbial composition, and immune rhythms, making it difficult to directly extrapolate the experimental conclusions. Additionally, existing imaging and body fluid markers lack cell‐type specificity and cannot simultaneously assess BBB permeability, central glial activation, and peripheral immune cell function, resulting in continuous indecision in clinical intervention trials regarding subject stratification, intervention timing, and efficacy endpoints. Furthermore, most studies remain at the level of gene expression or single‐cell populations, lacking functional validation by integrating multiomics data and long‐term follow‐up. There is also a lack of systematic assessment of the infection risk and neuropsychiatric comorbidity associated with the long‐term use of biologics.

In summary, while immune system dysfunction is the core driver of RA, complex bidirectional communication within the neuro‐immune network acts as a significant amplifier of persistent inflammation, progressive tissue destruction, and the substantial accompanying disease burden. Future breakthroughs need to involve precise dissection of the key nodes within these interactive networks, such as specific neuropeptide receptors, regulation of sympathetic tone, and restoration mechanisms of the HPA axis, to develop synergistic therapeutic strategies that move beyond traditional immunosuppression. Furthermore, overcoming the aforementioned challenges requires the development of coordinated therapies spanning both the neural and immune domains (e.g., biologics targeting neurotransmitter receptors and neuromodulation techniques combined with immunosuppressants) and establishing corresponding biomarker monitoring systems.

##### 3.2.1.2 Systemic Lupus Erythematosus

SLE involves the abnormal activation of the immune system that attacks the tissues of the body. This autoimmune storm also fiercely invades the nervous system, leading to its extensive and complex neuropsychiatric manifestations (NPSLE) [282]. Although the specific etiology of SLE involves multiple factors, such as genetics, environment, and estrogen, the core driver of nerve damage clearly points to uncontrolled autoimmune responses [283].

The core driver of this pathological process stems from the attack of autoantibodies, such as anti‐dsDNA and antiphospholipid antibodies, on cerebrovascular endothelial cells. This trigger complement activation, endothelial apoptosis, and degradation of TJ proteins, such as zonula occludens‐1 and claudin‐5 (CLDN5), which damage the integrity of the BBB [284]. The breakdown of the BBB results in high levels of circulating proinflammatory factors such as IL‐6 and TNF‐α to flood into the CNS. IL‐6 further disrupts the BBB by upregulating MMP‐9, and together with TNF‐α, strongly activates cerebrovascular endothelial cells, upregulates the adhesion molecules ICAM‐1 and VCAM‐1, and recruits a large number of peripheral immune cells to infiltrate the brain parenchyma [285].

Factors such as IFN‐γ and IL‐1β released by these invading immune cells polarize resting microglia into a proinflammatory, neurotoxic M1 phenotype, which continuously produce ROS, NO, and chemokines (such as CXCL10), directly damaging neurons and amplifying local inflammation [286]. These inflammatory factors also affect astrocytes: IL‐6 and TNF‐α inhibit their key glutamate transporter, leading to the accumulation of excitotoxic glutamate; their secretion of neurotrophic factors (such as BDNF) decreases, further depriving neurons of survival support [287]. Eventually, this neuroinflammatory storm, initiated by peripheral autoimmunity, is transmitted through BBB leakage and immune cell infiltration and is amplified by activated neuroglial cells, completely disrupting the neural microenvironment, neurotransmitter balance, and synaptic function. Its destructive power is directly reflected in the diverse neuropsychiatric symptoms of patients, and the levels of these inflammatory factors in CSF are significantly correlated with symptom severity [288].

Current research on the neuro‐immune crosstalk in SLE is marred by multiple controversies and deficiencies. There is a lack of consensus on etiology, and the direct causal relationship between antiphospholipid antibodies and IFN‐α signaling and central damage has not been unanimously confirmed. Diagnosis relies on exclusionary methods. However, the sensitivities and specificities of these biomarkers are insufficient. There are significant laboratory differences in serum and CSF tests, and the association between the severity and prognosis of neurological and psychiatric symptoms is unclear. Animal models have obvious limitations, and it is difficult to replicate long‐term low‐level inflammatory characteristics and dynamic changes in BBB permeability in the context of the human multigene background. Moreover, the clinical trial data are also limited. Treatment of neurological lupus is mostly based on small‐sample studies or expert consensus, lacking randomized controlled evidence of biological agents and novel JAK inhibitors, making it difficult to assess their efficacy and safety. The research design was highly heterogeneous, with significant racial and sex differences. An insufficient follow‐up time limits the observation of late neurological and vascular events, further amplifying the inconsistency in the conclusions.

The mechanistic complexity of NPSLE far exceeds current understanding. It remains challenging to definitively distinguish whether neurological symptoms that result directly from systemic SLE immune activity are driven by localized immune/inflammatory events primarily within the CNS, or stem from a complex bidirectional interplay between the two. Furthermore, the activation states and phenotypes of microglia and astrocytes exhibit high heterogeneity across the different stages of NPSLE, various brain regions, and distinct pathological subtypes. Reliable biomarkers for early identification of high‐risk patients, differentiation of NPSLE pathological subtypes, and objective assessment of disease activity and treatment responses are also lacking. Future breakthroughs will require not only continued in‐depth analysis of the precise interactions between specific autoantibody subtypes and neural targets and the development of strategies to repair the BBB or precisely modulate detrimental glial activity, but also the establishment of a more refined NPSLE classification system integrating clinical phenotypes, neuroimaging, and CSF/blood biomarkers. This system should facilitate early diagnosis, subtype classification, prognostic prediction, and treatment monitoring.

##### 3.2.1.3 Psoriasis

Psoriasis is a common chronic, stubborn and recurrent inflammatory skin disease, and it belongs to an autoimmune disorder. Although genetic and environmental factors are involved in the onset, immune abnormalities are the engine of the inflammatory cascade reaction [289].

The core driving factor of psoriasis is the dysregulation of the immune system, specifically the chronic inflammation mediated by the IL‐23/Th17 axis, and the involvement of the nervous system significantly amplifies and maintains this pathological process. Chronic stress serves as a key trigger, disrupting the function of the HPA axis, leading to an imbalance in immune regulation, such as impaired Treg cell function, providing a favorable environment for excessive immune activation [290]. At the skin site, neural signals are closely intertwined with the immune response: external stimuli activate DCs to initiate the core pathway of Th17 differentiation. Neurotransmitters released by sensory nerves, such as SP and CGRP, play a promoting role, enhancing the maturation and function of DCs, and synergistically promoting the secretion of IL‐17 and IL‐23 by Th17 cells [291]. IL‐17 directly acts on keratinocytes, driving their excessive proliferation, releasing inflammatory mediators, and recruiting more immune cells. IL‐23 further maintains the Th17 response and stimulates keratinocytes to produce antimicrobial peptides/cytokines, jointly constituting the core driving force of epidermal proliferation and inflammation. It is notable that the activation of acid‐sensing ion channel 3 on sensory neurons induces CGRP release, which has been proven to be the key upstream signal for initiating the IL‐23/Th17 inflammatory cascade, directly triggering the psoriasis‐like phenotype [292]. The polarization of macrophages is also influenced by the neuro‐immune dialogue: although NE can induce the anti‐inflammatory M2 type, in the inflammatory environment of psoriasis, factors such as neuropeptides often interfere with this protective mechanism, promoting the transformation of macrophages to the proinflammatory M1 type, and subsequently releasing factors such as TNF‐α and IL‐1β to amplify local inflammation [293]. The latest research has revealed a deeper neuro‐immune integration: inflammatory‐induced tenasin‐C⁺ fibroblasts are located at the dermal‐epidermal junction, not only promoting abnormal growth of sensory nerve axons but also forming “neuro‐immune synapse”‐like structures, providing a physical platform for the direct regulation of immune cells such as DCs and T cells by neural signals, ultimately exacerbating skin inflammation [294].

The research on the neuro‐immune crosstalk in psoriasis currently faces multiple controversies and limitations. On one hand, the correlations between peripheral inflammatory markers and central symptoms (such as depression, anxiety, and cognitive impairment) vary in different cohorts, and the causal sequence remains unclear. On the other hand, there is a lack of a unified neuro‐psychiatric outcome assessment tool in clinical trials, and the skin‐related indicators such as dermatology life quality index and psoriasis area and severity index cannot be collected simultaneously with the neuro‐symptom scales, which limits cross‐study comparisons. Moreover, treatments targeting pathways such as IL‐23 and TNF‐α may trigger rare but serious neurological adverse reactions, whose mechanisms are not yet clarified, and the long‐term effects of existing biological agents on central immune homeostasis lack sufficient follow‐up data. The research samples are mainly of European descent, and data for Asian, African, and other populations are insufficient, which limits the generalizability of the conclusions. Animal models can only partially simulate the chronic low‐level inflammation and multisystem comorbidity in humans, making it difficult to verify the causal chain of peripheral‐to‐central immune interaction. Finally, there is no standardized multiomics scheme to integrate dynamic information on peripheral immune cell function, BBB permeability, and central glial activation, which leaves the intervention timing, dosage, and population stratification strategies lacking empirical evidence.

In conclusion, the neuro‐immune crosstalk in psoriasis is not an independent event, but is deeply embedded and strengthened through multiple levels such as neuropeptides, channel activation, and cell interactions, forming an immune‐inflammatory circuit centered on Th17. Future research is urgently needed to analyze the key nodes of these interactions, which will pave the way for developing new therapeutic strategies targeting the neuro‐immune interface such as neuropeptide antagonists and sensory nerve modulation to synergistically inhibit the immune‐inflammatory circuit.

#### 3.2.2 Neuropsychiatric Disorders

Neuro‐immune interactions play a pivotal role in the pathogenesis of neuropsychiatric disorders such as depression and schizophrenia. Their pathological basis involves multidimensional crosstalk across regulatory networks, encompassing immune‐inflammatory activation, dysregulation of neurotransmitter systems, and alterations in neural plasticity.

##### 3.2.2.1 Depression

Depression is a mental disorder characterized by core symptoms of significantly and persistently depressed mood, diminished interest, and cognitive dysfunction. A series of past studies have indicated that depression involves structural abnormalities in brain tissue and neuroinflammation [295]. Depression correlates with various brain alterations involving neuronal and astroglial compromise, neuroinflammation, activated microglia polarizing to M1 phenotype, impaired astrocyte activity, and diminished oligodendrocyte functionality [296, 297]. Such alterations can result in impaired neural circuit connectivity and metabolic dysregulation impacting synaptic signaling.

The activation of the peripheral immune system induced by chronic stress is closely linked to depression. Stimulated macrophages and T lymphocytes secrete proinflammatory mediators (including IL‐1β, IL‐6, TNF‐α). These molecules cross into the CNS via a compromised BBB, coinciding with pathological migration of peripheral T cells and monocytes into brain tissue. Within the parenchyma, these infiltrating cells discharge ROS and chemokine molecules, establishing a potent “peripheral‐central immune interface” [298, 299]. Evidence also indicates that in patients with major depressive disorder (MDD), levels of inhibitory cytokines (including IL‐4, IL‐10, and IL‐12p70) are decreased, while neurotoxic cytokines (including CCL5) are relatively elevated [300], and oxidative stress levels are increased [301]. Conversely, when depressive symptoms remit, levels of cytokines (e.g., IL‐16, TNF, M‐CSF, IL‐4), growth factors (including PDGF and stem cell growth factor), and chemokines (CCL3, CCL4, CCL5, CXCL8, and CXCL12) increase [302].

Peripheral immunity can drive central pathological processes: On one hand, cytokines activate glial cells (particularly microglia), inducing neuroinflammation and releasing toxic mediators like ROS and NO, directly damaging neurons [303]. On the other hand, these factors significantly inhibit tryptophan hydroxylase activity, leading to reduced synthesis of the key mood‐regulating neurotransmitter serotonin (5‐HT) [304]. However, direct evidence of brain tissue changes in depression patients largely comes from postmortem studies. Postmortem prefrontal cortex from completed suicide subjects shows elevated levels of proinflammatory cytokines, such as TNF, IL‐1β, and IL‐6 [305], along with neuroinflammation and M1 microglial activation in specific brain regions [306]. Neurons also exhibit reduced dendritic branching and arborization, as well as decreased neuronal density [296, 307]. Furthermore, deep involvement of neuroendocrine pathways further amplifies immune imbalance: Dysfunction of the HPA axis itself can exacerbate inflammation, while diminished vagus nerve signaling weakens a crucial anti‐inflammatory pathway [308, 309]. The GBA plays a key pivotal role in this process, allowing gut‐derived immune signals (including cytokines) to directly influence central neurotransmitter systems and endocrine regulation via vagal afferent fibers [308, 309].

More clinical data and basic research are needed to distinguish between recurrent depression, differences between first and multiple episodes, stages of episodes (acute, remission, partial remission), treatment‐resistant depression, and chronic depression. Future research priorities include developing drugs to prevent disease relapse by targeting the repair of the gut barrier and BBB, and inhibiting key inflammatory signaling pathways.

##### 3.2.2.2 Schizophrenia

Schizophrenia is a severe chronic psychiatric disorder characterized by multidimensional dysfunctions in cognition, affect, and behavior. Its etiology involves complex synergism among genetic susceptibility, aberrant neurodevelopment, environmental factors (e.g., infections, stress), and neuro‐immune interactions, ultimately leading to disrupted brain network functionality [310].

The neuro‐immune crosstalk in this disease exhibits bidirectional dynamics: immune abnormalities drive pathological progression by inducing neuroinflammation, disrupting neurotransmitter balance, and impairing neurodevelopment. Specifically, hyperactivation of the HPA axis in patients elevates cortisol levels, which on one hand suppresses immune functions of T cells and NK cells to compromise immune surveillance, and on the other hand acts via GCRs on the hippocampus to exacerbate neural damage, forming a vicious “immune suppression‐neural injury” cycle [311]. Concurrently, genetic factors (e.g., Complement Component C4 gene variants) or environmental stimuli (e.g., chronic inflammation) induce microglial overactivation, causing excessive synaptic pruning in key brain regions like the prefrontal cortex and hippocampus; this aberrant neural circuit connectivity constitutes the pathological basis of early‐stage (e.g., prodromal phase) cognitive decline [312]. Notably, dysregulated neuromodulation (e.g., HPA axis and vagus nerve dysfunction) further amplifies immune imbalance, with both systems interacting through complex networks to collectively shape the pathophysiological phenotype.

The core controversy in the current research on neuro‐immune cross‐talk in schizophrenia lies in the uncertainty of the direction of immune activation: The traditional theory of enhanced neuroinflammation is challenged, and some severe patients exhibit contradictory phenomena of reduced microglial cell activity and elevated immune‐inhibitory molecules. The causal relationship between immune markers and symptoms is difficult to establish, and it fluctuates and contradicts in different stress models, making it impossible to distinguish between causation and comorbidity associations. The abnormal complement‐mediated synaptic pruning, although a genetic risk factor, has different dynamic contributions at different disease stages, which is inconsistent. The research difficulty stems from the extremely complex nature of the neuro‐immune network: The bidirectional regulation of microglia and neurotransmitter systems involves multiple receptors and temporal dynamics, making it difficult to simulate in experimental models; the human evidence of peripheral immune cells infiltrating the CNS is weak; the significant clinical heterogeneity makes it difficult to interpret the increased specificity of autoantibodies in different subtypes. The key deficiencies are concentrated in three major bottlenecks: Animal models cannot accurately simulate human chronic neuro‐immune dysregulation, and the results of acute and chronic interventions are significantly different; the lack of universal biomarkers makes immunotherapy effective only in specific inflammatory subgroups; peripheral blood indicators are difficult to reflect the central immune status, and the application of CSF detection and neuroimaging is limited; the long‐term safety assessment system for immune regulation treatment has not been established, and the integration of interdisciplinary methods is insufficient.

### 3.3 Environment–Immune–Nervous System Axis

The interaction between the nervous system and the immune system is profoundly influenced by environmental factors, together forming an “environment–immune–nervous system axis.” Environmental exposures such as allergens and pollutants, as key triggering factors, continuously act on local immune cells and sensory nerves in barrier sites like the skin, respiratory tract, and intestines. This interaction can disrupt immune homeostasis, cause dysregulation of neuro‐immune crosstalk, and trigger abnormal inflammatory signals and neuronal sensitization. The dysregulation of this axis is the core pathogenesis of environment‐related diseases such as allergic diseases, asthma, and gastrointestinal disorders.

#### 3.3.1 | Allergies and Asthma

In allergies and asthma, sensory nerves in the nasal cavity or skin release neuropeptides such as SP and CGRP when stimulated by allergens. SP can induce mast cell degranulation and HA release, which in turn triggers urticaria [313]. CGRP promotes the expression of adhesion molecules in vascular endothelial cells, recruiting eosinophils and Th2 cells. At the same time, downregulated function of sympathetic nerve β2 receptors weakens the inhibition of Th2 cells, exacerbating airway inflammation. During allergic reactions, the vagus nerve‐mediated CAP is inhibited (IL‐6 downregulates α7nAChR expression), losing its anti‐inflammatory effect on mast cells and macrophages [314]. This reveals that allergic diseases essentially result from a bidirectional imbalance in the neuro‐immune network: neuropeptides initiate immune activation, while immune factors in turn sensitize nerve signals, forming a persistent inflammatory cycle.

Similarly, the development of asthma is essentially an imbalance in the bidirectional regulation between airway nerves and the immune system. It drives airway inflammation, hyperreactivity, and tissue remodeling through a pathological axis of “nerve‐initiating immunity, immunity acting back on nerves, and structural remodeling solidifying interactions.” When inhaled allergens activate airway sensory nerves, they also release SP and CGRP. SP activates mast cells via NK1 receptors, releasing HA and leukotriene (LTC4) to cause bronchial smooth muscle contraction and vascular leakage. IL‐33 released by immune cells upregulates TRPV1 channels in nerve endings, leading to airway hypersensitivity [315]. In the chronic inflammatory phase, desensitization of sympathetic nerve β_2_‐AR and excessive vagal tone further aggravate inflammation. IL‐13 secreted by Th2 cells induces nerve fiber proliferation, forming a vicious cycle. During airway remodeling, SP stimulates fibroblasts to secrete TGF‐β1, promoting myofibroblast differentiation and collagen deposition; CGRP induces vascular endothelial cells to express VEGF, facilitating airway angiogenesis; and eosinophils release NGF and other factors to promote nerve sprouting. These processes ultimately result in airway hyperreactivity, persistent inflammation, and irreversible tissue damage.

At present, in the research on the neural–immune crosstalk in allergies and asthma, there are various controversies, difficulties and deficiencies. The main controversy lies in the inconsistent conclusions regarding the roles of key molecules or pathways. For instance, the immunomodulatory effects of some neuropeptides often yield contradictory results (proinflammatory or anti‐inflammatory) due to differences in sample sources, model types or detection methods. There is no consensus on the dynamic effects of the disease at different stages. The research difficulties are mainly concentrated on the model and technical aspects. A major limitation of animal models is their inability to capture the full complexity of the human neural–immune microenvironment. The differences in neural innervation, immune cell composition and disease heterogeneity limit the extrapolation of basic research to human pathophysiology. The existing technologies are unable to precisely capture the real‐time dynamic communication and molecular exchange between nerve endings and immune cells. The analysis of spatial positioning and temporal changes is insufficient. The research deficiencies are reflected in the lack of systematic understanding and clinical translation. Current research mostly focuses on a single molecule or local effects, lacking a systematic analysis of the overall regulatory mechanism of the neural–immune–endocrine network. There is insufficient understanding of the specific differences in neural–immune crosstalk in different disease subtypes. The potential targets of basic research have a low correlation with clinical phenotypes. Target intervention strategies are difficult to be generalized due to large individual differences and unstable efficacy. A precise diagnosis and treatment system based on neural–immune characteristics has not yet been established.

#### 3.3.2 Gastrointestinal Diseases

The intestine, a unique organ densely populated with nerve and immune cells, forms a complex microenvironment. Within this environment, the ENS engages in continuous, precise neuro‐immune crosstalk with various immune cell populations. This dynamic interaction is crucial for maintaining intestinal homeostasis, regulating inflammatory responses, and defending against pathogens, serving as the core of intestinal health. However, when this balance is disrupted and neuro‐immune crosstalk becomes disordered, it becomes the core pathological basis for various gastrointestinal diseases such as IBS and IBD.

##### 3.3.2.1 Irritable Bowel Syndrome

IBS is characterized by abdominal pain, abnormal bowel movements (diarrhea/constipation), and intestinal dysfunction, with its pathological core residing in neuro‐immune crosstalk along the “gut–brain–immune axis” [316]. This dynamic network drives disease onset and progression while modulating symptom manifestation. Specifically, IBS patients exhibit reduced gut microbiota diversity accompanied by dysbiosis (e.g., decreased Bacteroides, increased Escherichia coli), where pathogen‐derived metabolites like LPS activate intestinal mucosal TLR4 receptors, inducing macrophages and DCs to secrete IL‐6 and TNF‐α; concurrently, diminished production of Bacteroides‐derived SCFAs compromises their anti‐inflammatory protective effects on enteric neurons [317]. Mediators including HA, 5‐hydroxytryptamine (5‐HT), and IL‐6 released by local immune cells (mast cells, T cells) in the intestinal mucosa constitute key effector mechanisms: mast cell‐derived tryptase directly activates sensory nerve endings in the gut wall, while 5‐HT transmits pain signals to the CNS by stimulating dorsal horn neurons of the spinal cord [318, 319]. Aberrant bidirectional regulation in the GBA further amplifies pathology—gut‐derived immune‐inflammatory factors act on the CNS via the vagus nerve and bloodstream, disrupting HPA axis and limbic system functions to trigger mood disorders like anxiety and depression; conversely, psychological stress exacerbates intestinal immune activation through neurotransmitters such as cortisol and catecholamines, forming an “immune–neuro‐psychological” vicious cycle [320]. In essence, IBS pathogenesis results from synergistic interactions among microbial dysbiosis, immune activation, and neural dysregulation, wherein neuro‐immune interplay establishes a self‐reinforcing mechanism via the bidirectional “peripheral inflammation‐central regulation‐peripheral feedback” pathway.

At present, in the research on the neuro‐immune crosstalk in IBS, there are many controversies, deficiencies, and difficulties. The controversies mainly lie in the specific effects of key regulatory mediators (such as neuropeptides released by intestinal nerves and cytokines secreted by immune cells) on symptom regulation. Due to the differences in subtypes of the research subjects and the different sample sources, contradictory results of sensitizing or inhibiting effects often occur, and the dynamic role of these mediators in different stages of the disease has not yet reached a consensus. The deficiencies in research include a lack of systematic understanding, as current studies mostly focus on a single molecule or local pathways, lacking a systematic analysis of the overall regulatory mechanism of the neuro‐immune–gut microbiota–endocrine network. There is also insufficient understanding of the specific differences in neuro‐immune crosstalk in different triggers and subtypes of IBS. The difficulties in research lie in the model and technical aspects. Animal models are difficult to fully simulate the complex pathological features of human IBS, especially the differences in gut microbiota composition, nerve control patterns and psychological factors compared with humans, which lead to limitations in the extrapolation of basic research results to clinical applications. At the same time, neuro‐immune interactions involve multiple levels such as intestinal mucosa, intestinal ganglia, CNS, and so on. Current technologies are unable to accurately capture the real‐time dynamic communication process between nerve endings and immune cells, and the analysis of spatial positioning and temporal changes is still insufficient.

Therefore, the onset of IBS is essentially the result of the synergistic interaction among intestinal flora imbalance, local immune activation, and neurological dysfunction (including enteric nerves and central nerves). Here, the nervous and immune systems form a self‐reinforcing mechanism for the persistence of the disease through a bidirectional pathway of “peripheral inflammation‐central perception/regulation—peripheral functional feedback.” Looking to the future, in‐depth analysis of the interactions among key nodes in this complex crosstalk network, especially the direct dialogue mechanism between specific immune cell subsets and neurons, as well as the development of targeted therapies that can effectively interrupt this vicious cycle, such as regulating the flora–immune interface or the neural–immune interface, will be important directions for understanding the overall picture of IBS and achieving precise intervention.

##### 3.3.2.2 Inflammatory Bowel Disease

Over the past several years, the role of neuroimmunology in IBD has been extensively validated. The ENS is no longer viewed merely as a regulator of motility and secretion but is recognized as an active conductor of mucosal immunity. Catecholaminergic, cholinergic, and gamma‐aminobutyric acid (GABA)ergic neurons within the ENS express MHC class I and costimulatory molecules, enabling direct antigen presentation to CD8⁺ T cells to either trigger or terminate local inflammation [321]. GABA secreted by intestinal GABAergic neurons inhibits the proliferation and IL‐17A secretion of group 3 innate lymphoid cells (ILC3s) via Gabbr1/2 receptors, thereby maintaining barrier homeostasis. When the GABA‐C/EBP‐β–IGFBP7 axis is suppressed in IBD patients, ILC3s become hyperactivated, rapidly amplifying inflammation [322]. The ENS modulates mucosal immunity by inhibiting Th1 cell activation through VIP released by intestinal neurons, although VIP secretion decreases during inflammation [323]. The ENS regulates mast cell and T cell activation as well as inflammatory factor release through neuropeptides such as SP and CGRP, alongside autonomic nerves. For instance, SP released by the ENS activates mast cells to release HA, exacerbating intestinal mucosal inflammation [324, 325]. Conversely, cytokines produced by immune activation (e.g., IL‐6, TNF‐α) sensitize intestinal sensory nerves, enhance visceral hypersensitivity, and affect the CNS via the vagus nerve, forming a vicious cycle of “intestinal inflammation‐central sensitization.” Simultaneously, the CAP of the vagus‐spleen‐gut axis has been repositioned: α7nAChR agonists or chronic VNS significantly reduce TNF‐α and IL‐6 levels, decrease neutrophil infiltration, and demonstrate efficacy comparable to anti‐TNF‐α monoclonal antibodies in dextran sulfate sodium salt (DSS) and trinitro–benzene–sulfonic acid murine models [326]. However, the “plasticity” of the ENS leads to phenotypic shifts in inflammatory environments: sustained GC exposure induces a proinflammatory enteric glial cell subset that secretes CSF1, driving the monocyte‐TNF axis, while TGF‐β2‐mediated neuronal transcriptional immaturity disrupts intestinal motility, further aggravating microbial translocation [321].

The discovery of the microbiota–metabolite–neural axis has significantly advanced IBD research. Microbiota‐derived SCFAs not only inhibit HDACs via FFAR2/3 receptors to enhance Treg cell function but also directly act on Olfr78 receptors on enteric neurons to promote 5‐HT synthesis and maintain ENS rhythmicity. Conversely, indole derivatives generated by tryptophan metabolic dysregulation are significantly elevated in IBD patient plasma. These metabolites activate TRPV1⁺ sensory neurons via the AHR pathway, triggering CGRP release and inducing Th17 expansion, thereby establishing a “microbiota–neural–immune” positive feedback loop. Clinical metagenomic‐metabolomic studies further confirm that butyrate‐producing bacteria (Faecalibacterium, Roseburia) positively correlate with enteric nerve density, whereas Candida albicans enrichment coincides with synchronized increases in neuronal sprouting and IL‐17A peaks [321]. Notably, the dual “mechano‐chemical” sensing capacity of sensory neurons to the microenvironment has been confirmed: Piezo1 channels detect luminal pressure changes and regulate Ach release from cholinergic neurons, thereby inhibiting NLRP3 inflammasome activation in macrophages.

Despite these discoveries offering multidimensional therapeutic targets for IBD, significant knowledge gaps and challenges persist. The specific division of labor between the ENS and CNS across different IBD stages remains unclear: while the ENS dominates local inflammation during acute phases, how central sensitization intervenes and solidifies the gut–brain feedback loop in chronic stages requires deeper investigation. Current animal models inadequately recapitulate the diversity and plasticity of the human ENS, particularly lacking integrated organoid–microfluidic–live imaging platforms that incorporate microbiota, immune, and neural networks. Finally, interventions targeting the microbiota–neural interface remain nascent: strategies to reconstruct an “anti‐inflammatory–proneural” microbiota through FMT, postbiotics, or CRISPR‐engineered bacteria—while avoiding reactivation of TRPV1⁺ neurons by potential pathogens—represent a critical scientific challenge.

## 4 Neuro‐Immune Crosstalk in Tumors

The nervous system precisely orchestrates organ development, maintains physiological homeostasis, and actively shapes tumor initiation and progression [327]. The significant influence of neural activity on tumors establishes the “neuro‐tumor interface” as a key regulatory hub central to tumorigenesis and progression [328]. Moreover, the immune system, an indispensable core component of the TME, directly dictates the trajectory and outcome of the oncogenic process through its functional states, including immune surveillance, immune editing, and immune escape [329].

Neural infiltration within the TME is an active process driven by multiple factors, involving complex signaling interactions among tumor cells, immune cells, and stromal cells [330]. Tumor cells overproduce neurotrophic factors (such as NGF and BDNF) and axon guidance molecules (like Netrin‐1), which attract nerve fibers into the tumor and may promote new nerve formation [331]. Immune cells such as M2‐type macrophages directly support neural growth by secreting NGF and BDNF, while Treg cells contribute to an immunosuppressive microenvironment via IL‐10 and TGF‐β, weakening antitumor immunity [332]. Infiltrating nerves further modulate immune responses by releasing neurotransmitters including NE: adrenergic signaling promotes M2 macrophage polarization and enhances MDSC function, whereas cholinergic signaling reduces inflammation via α7nAChR, together forming a “neural–immune–tumor” positive feedback loop [333]. This loop accelerates nerve invasion and promotes angiogenesis, matrix remodeling, and tumor metastasis. Therefore, understanding this mechanism not only reveals the regulatory role of nerves in tumors, but also provides a basis for treatment strategies targeting neural signaling or guidance molecules.

Given the pivotal roles the nervous and immune systems play in tumor development and progression, coupled with the complex and dynamic bidirectional communication existing between them, the in‐depth investigation of the neuro‐immune crosstalk and its regulatory mechanisms within the TME has therefore emerged as a high‐profile frontier research focus in contemporary cancer biology.

### 4.1 Peripheral Tumors

In the TME of peripheral solid tumors, the immune system suppresses tumor development by identifying and eliminating abnormal cells via immune surveillance [334]. In the early stages of tumor progression, immune effector cells, such as cytotoxic T lymphocytes (CTLs) and NK cells, can specifically recognize antigens on tumor cell surfaces and induce targeted killing. However, tumor cells can escape immune clearance through various mechanisms in a dynamic process called cancer immune evasion [335]. Key strategies include abnormal activation of immune checkpoints, where tumor cells overexpress immune checkpoint molecules such as PD‐L1, binding to PD‐1 receptors on T cells to inhibit their activation and cytotoxic function [336]. Additionally, the TME actively recruits immunosuppressive cell populations to establish a protumor growth immunosuppressive microenvironment. Predominant effector cells comprise TAMs, which adopt an M2‐type polarization and inhibit T cell activity through secretion of immunosuppressive mediators including IL‐10 and TGF‐β [337, 338]; Treg cells, which directly inhibit effector T cell activity through cell contact‐dependent suppression and secretion of IL‐10 and TGF‐β; and MDSCs, which block T cell proliferation and induce apoptosis by releasing mediators such as arginase‐1 and ROS [339]. These mechanisms collectively weaken the antitumor immune response, promoting tumor immune evasion and malignant progression.

**FIGURE 5:**
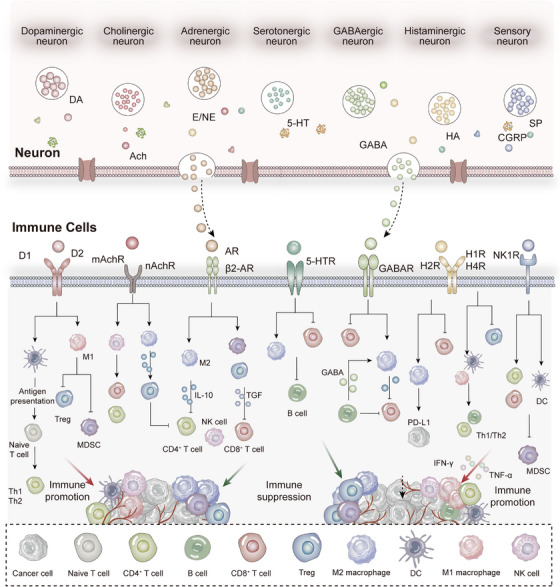
The interplay between neurons and immune cells significantly influences peripheral tumor progression. Neurotransmitters secreted by neurons playing a crucial role in modulating immune responses within the tumor immune microenvironment. For instance, Ach released from synaptic terminals can promote the differentiation of M2‐type macrophages and the formation of Tregs by engaging acetylcholine receptors on immune cells, thereby inhibiting the function of CD4^+^ T cells. E and NE, through the activation of AR, facilitate the formation of MDSCs, Tregs, and M2 macrophages, which in turn secrete immunosuppressive factors such as IL‐10 and TGF‐β, inhibiting the functionality of both CD4^+^ and CD8^+^ T cells. 5‐HT, secreted by serotonergic neurons, inhibits the normal functions of CD4^+^ and CD8^+^ T cells through the activation of its corresponding receptors. The GABAergic signaling pathway predominantly promotes tumorigenesis, with B cells further secreting GABA to suppress antitumor immune responses. Histamine signaling exhibits a complex role, with the potential to either promote or inhibit tumor growth. Activation of H2 receptors primarily enhances the activation of suppressive immune cells, while activation of H1 and H4 receptors promotes antitumor immune responses by increasing the secretion of factors such as TNF‐α and IFN‐γ. Additionally, dopaminergic and sensory neuronal signals play a pivotal role in immune promotion, enhancing antigen presentation and promoting the differentiation of T cells into effector T cells, thereby exerting antitumor effects. *Abbreviations*: DA, dopamine; Ach, acetylcholine; E, epinephrine; NE, norepinephrine; 5‐HT, serotonin; GABA, gamma‐aminobutyric acid; HA, histamine; SP, substance P; CGRP, calcitonin gene‐related peptide; D1, D1‐like dopamine receptors; D2, D2‐like dopamine receptors; AchR, acetylcholine receptors; AR, adrenergic receptors; NK1R, neurokinin 1 receptor. TGF‐β, transforming growth factor‐beta; TNF‐α, tumor necrosis factor‐alpha; IFN‐γ, interferon‐gamma; DCs, dendritic cells; Tregs, regulatory T cells; MDSCs, myeloid‐derived suppressor cells; IL‐10, interleukin‐10; NK cell, natural killer cell.

The PNS is an important component of the cancer microenvironment, and has been proven to support cancer progression [340]. It engages in sophisticated bidirectional communication networks with tumor‐infiltrating immune cells, thereby modulating the initiation and progression of neoplastic processes. Neurons and glial cells secrete an array of signaling molecules capable of binding to distinct receptors on immune cell surfaces, directly influencing cellular activity and significantly altering immune cell activation, migratory patterns, and effector functions, ultimately shaping the immune landscape of the TME [341]. Conversely, immune cells reciprocate by releasing cytokines, chemokines, and other signaling entities that modulate neuronal activity [342]. This dynamic interaction influences neuronal behavior and impacts tumor growth and progression (Figure 5).

#### 4.1.1 Neurotransmitter‐Driven Immune Modulation

Neurotransmitters are key signaling molecules that mediate signal transduction between neurons [343]. Within the TME, multiple immune cell types express specific neurotransmitter receptors, enabling neurotransmitters to significantly regulate tumor‐associated immune responses through ligand‐receptor interactions.

##### 4.1.1.1 Dopaminergic Signaling

DA, a central catecholamine neurotransmitter, exerts significant regulatory influence over a multitude of physiological and pathological processes within the nervous system. Despite its inability to cross the BBB, DA is released by postganglionic sympathetic nerves in the PNS, where it plays a crucial role in modulating vascular tone and cardio‐renal functions [344]. Recently, the intricate relationship between DA and tumorigenesis has garnered substantial attention, underscoring its vital regulatory function within the TME. The actions of DA are largely mediated through its specific receptors, which are divided into two primary categories: D1‐like receptors (comprising DRD1 and DRD5) and D2‐like receptors (including DRD2, DRD3, and DRD4) [345]. Notably, these receptors are expressed across tumor cells, tumor‐associated vascular endothelial cells, and immune cells, facilitating DA's capability to modulate oncogenic signaling pathways and immune responses [346].

In the realm of innate immune cells, particularly within myeloid populations such as macrophages, DCs, and MDSCs, DA receptors (DRs) exhibit notably high expression levels. MDSCs primarily express D1‐like receptors, including DRD1 and DRD5, which DA activates to suppress the functionality of MDSCs’ [339]. This activation alleviates their inhibitory effects on T cell proliferation and IFN‐γ production, thereby bolstering antitumor immune responses and effectively decelerating tumor growth [347]. Furthermore, the activation of DRs plays a pivotal role in modulating the shift between proinflammatory (M1) and anti‐inflammatory (M2) macrophage phenotypes [348]. DRD2 interacts with proteins such as β‐arrestin2 and DEAD‐box helicase (5DDX5), limiting NF‐κB signaling and promoting M1 polarization, which effectively inhibits the growth of breast cancer cells [349]. Additionally, DA regulates TAMs through DRD4 by constraining the cAMP/PKA/p38 signaling pathway, thus reducing protumor inflammatory signals [350]. This modulation suppresses M2 differentiation by TAMs, markedly enhancing chemotherapy efficacy in murine pancreatic cancer models [350]. In the context of DCs regulation, DCs can autonomously secrete DA, which interacts with DRD5 to impair TRAF6‐mediated NF‐κB activation, thereby modulating macrophage functions. This signaling cascade further enhances the production of IL‐12 and IL‐23, fostering the differentiation of naïve CD4^+^ T cells into Th17 cells and amplifying inflammatory responses [351, 352].

In the realm of adaptive immunity, DA exerts a multifaceted and dynamic regulatory influence, intricately linked to the specific DRs subtypes engaged. Within NK cells, activation of D1‐like receptors enhances cytotoxicity through the cAMP/PKA/CREB signaling cascade, whereas D2‐like receptor activation exerts inhibitory effects [353]. For instance, DRD1 expression shows considerable correlation with the infiltration of macrophages, B cells, and CD8^+^ T cells in hepatocellular carcinoma [354]. Contrarily, some studies indicate that DA can inhibit NK cell activity and reduce IFN‐γ production by upregulating DRD5, thereby attenuating NF‐κB signaling [355]. Such discrepancies may arise from the intrinsic complexity of DA signaling, the dynamic modulation of receptor expression, and variations across experimental contexts. Immune cells residing in the TME, inflammatory sites, and normal tissues exhibit significant differences in phenotype and function. Conditions within the TME—such as hypoxia, high lactate levels, and low pH—can modulate the expression of DRs. An important factor contributing to these regulatory differences lies in the source of NK cells used across studies (e.g., peripheral blood, tumor‐infiltrating lymphocytes, or different cell lines), which may display considerable variation in the expression ratios of DRD1 to DRD5. Furthermore, experimental conditions employed in vitro, including cytokine preactivation status and coculture duration, also significantly influence the outcomes. On the other hand, DA often exerts biphasic or bell‐shaped curve effects on immune cells, closely correlated with its local concentration. At low concentrations, DA primarily facilitates immune activation via high‐affinity receptors, whereas at high concentrations, it mediates immunosuppression through low‐affinity receptors. In vivo, DA largely diffuses via volume transmission, resulting in considerable concentration gradients across different regions. Thus, immune cells located in various parts of the same microenvironment may be exposed to vastly different DA concentrations, leading to divergent regulatory outcomes. It is important to emphasize that results from in vitro and in vivo experiments frequently differ substantially. In living organisms, DA originates from multiple sources, including neuronal release, immune cell autocrine secretion, and gut microbiota metabolism. Its metabolic fate and interactions with other neurotransmitters or hormones (e.g., NE and Epi) form an exceedingly complex regulatory network, making it difficult to isolate the role of any single receptor. Although upregulation or dominant signaling through DRD5 often suppresses NK cell function, it should be noted that elevated cAMP levels may sometimes feedback‐inhibit immune function by inducing inhibitory molecules such as SOCS [356, 357]. More plausibly, DRD5 activation may not rely solely on the canonical Gs pathway but could couple to alternative signaling routes (e.g., β‐arrestin) or form heterodimers with D2‐class receptors, ultimately leading to suppression of NF‐κB signaling [358, 359]. This suggests that even within the same receptor family (e.g., DRD1 and DRD5), different members may exhibit subtle yet critical functional distinctions. Therefore, DA‐mediated immunoregulation should not be viewed as a simple “on‐or‐off” switch, but rather as a finely tuned network that depends on the cellular receptor expression profile, cell state, local DA concentration, and microenvironmental factors—demonstrating high context dependency. In T cells, the interaction between DA and its receptors is characterized by a complex interplay of concentration levels, receptor subtypes, and receptor densities, which collectively modulate T cell activation and differentiation [360]. DA's regulatory role in T cell activity is notably dynamic: it can stimulate resting T cells by engaging DRD3, facilitating the migration and homing of naïve CD8^+^ T cells, enhancing antigen presentation by DCs, and supporting both CD8^+^ T cell functionality and the formation of tissue‐resident memory (TRM) cells, thus bolstering antitumor immunity [361, 362]. Conversely, DA can suppress activated CD4^+^ and CD8^+^ T cells, inhibiting their proliferation and antitumor efficacy [363].

DA's influence within the TME extends beyond immune cell modulation, playing a crucial role in tumor vasculature dynamics. By stabilizing tumor blood vessels, DA significantly impacts tumor growth and metastasis. Studies have demonstrated that exogenous DA can inhibit VEGF A‐induced ERK1/2 phosphorylation and matrix metalloproteinase (MMP) 9 synthesis by activating DRD2 on endothelial progenitor cells, thereby hindering their mobilization and migration [364]. Furthermore, DA interacts directly with receptors on tumor cells, regulating their biological behaviors, such as inducing apoptosis. However, tumor cells often downregulate DR expression; the underlying mechanisms of this downregulation require further investigation [365].

Although the potential of the DA signaling pathway in tumor immune regulation is increasingly evident, current research still faces significant controversies, challenges, and deficiencies. The core controversy lies in the bidirectionality and context‐dependency of DA on tumor immunity. Whether it inhibits or promotes tumor development highly depends on the type of tumor, stage, composition of the microenvironment, and the temporal and spatial dynamics and concentration gradients of the DA signal. The complexity of the interplay between the nervous system, immunity, and tumors precludes broad generalizations. Key research hurdles include delineating the direct and indirect influences of DA on the in vivo immune microenvironment and deciphering its interactions with competing signaling pathways. The deficiencies include unclear receptor mechanisms, multiple DR subtypes with cell and tumor specificity, and the lack of systematic clarification of the conduction of downstream signals in immune cells and their interaction with tumor cell pathways. The model limitations are significant. Animal models are difficult to simulate the neural control and immune landscape of human tumors, and in vitro experiments lack the physiological neural–immune microenvironment. Dynamic studies are scarce. There is a lack of longitudinal studies on the dynamic changes of DA signaling and immunological consequences during tumor progression or treatment. The translational bottleneck is prominent. The immune regulation of DA‐based drugs faces controversy over dose effects, potential off‐target effects, and the problem of precise local delivery to tumors. In‐depth analysis of the precise action mode, overcoming model technical bottlenecks, and exploring safe targeting strategies are the directions that need to be broken through in the future.

In a broader sense, DA extensively influences immune cell functionality through its interaction with various DRs subtypes across different pathways. Each immune cell type within a given TME may exhibit distinct regulatory responses to DA, even when engaging the same receptor subtype. The heterogeneity of tumor immunology across different cancer types leads to unique and sometimes opposing alterations in immune cells when stimulated by DA.

This complex regulatory landscape is likely connected to the dynamic modulation of DRs during tumor progression and metastasis, although our understanding is still evolving. Future research should aim to elucidate the alterations in DRs expression during different tumor stages and explore the intricate regulatory mechanisms further, enhancing our ability to predict therapeutic outcomes.

##### 4.1.1.2 GABAergic Signaling

GABA is the principal inhibitory neurotransmitter in the CNS, operating through ionotropic (GABA‐A and GABA‐C) and metabotropic receptors (GABA‐B) to diminish neuronal excitability. Beyond its central nervous functions, the GABAergic system has been detected in immune and tumor cells, where it plays a critical role in modulating the TME by secreting GABA, thereby influencing adjacent cellular activities [366, 367]. Furthermore, the system holds potential in regulating antitumor immune responses and contributes to the tricarboxylic acid cycle, supplying energy for tumor cell sustenance [368, 369].

Studies have indicated that the accumulation of GABA is associated with tumorigenesis and progression. Elevated levels of GABA may facilitate glioma proliferation by generating succinate, thus fueling the tricarboxylic acid cycle in tumor cells [370]. In lung and colorectal cancers, GABA levels escalate with clinical staging and are significantly negatively correlated with patient survival, suggesting a potential promotive role for GABA in tumors [367]. Tumor cells achieve increased GABA synthesis through the aberrant expression of glutamate decarboxylase 1 (GAD1), inhibition of GAD1 reduces GABA production in tumor cells, thereby hindering tumor growth [367]. In breast cancer, the GABA‐A receptor pi subunit (GABRP) promotes cellular proliferation and differentiation through the activation of the ERK1/2 signaling pathway, enhancing the invasive potential of tumor cells [371]. Additionally, GABA influences angiogenesis within the TME by promoting the expression of fibroblast growth factor 2 in macrophages, thereby facilitating neovascularization [372].

GABAergic signaling exerts profound effects on the proliferation and function of various innate immune cells. In pancreatic ductal adenocarcinoma (PDAC), GABA engages with the π subunit of the A‐type receptor in cancer cells, activating the Ca2^+^/NF‐κB/CXCL5–CCL20 signaling cascade. This interaction significantly fosters macrophage infiltration, thereby expediting tumor growth and metastasis [373]. Through GABA secretion, tumor cells suppress the NF‐κB and STAT3 pathways, inhibiting M1 polarization and promoting M2 phenotype development via STAT6 activation [372], thus suppressing antitumor immunity [374]. Research further reveals that GABA, upon activating receptors on macrophages, directly boosts the synthesis and release of IL‐10 and enhances mitochondrial respiration, promoting monocyte transformation to an anti‐inflammatory phenotype within the TME [375]. Beyond its impact on macrophages, GABA receptor activation diminishes the proinflammatory capabilities of other antigen‐presenting cells such as DCs, inhibiting their secretion of proinflammatory cytokines IL‐6 and IL‐1β, thereby reducing the capacity of immune cells to elicit inflammatory responses [376].

In adaptive immune cells, GABA signaling exerts significant regulatory influences. This involves its interaction with GABA‐B receptors, where studies have demonstrated that GABA activation of these receptors in tumor cells inhibits GSK‐3β activity, thereby enhancing β‐catenin signaling. This pathway not only augments tumor cell proliferation but also obstructs CD8^+^ T cell infiltration into tumor tissues [367]. By blocking GABA in tumors, it is possible to suppress the β‐catenin pathway, thereby activating the expression of specific cytokines that promote DC infiltration. This process subsequently facilitates T cell infiltration and enhances tumor cell cytotoxicity [367]. Furthermore, GABA secreted by tumor or immune cells, particularly B cells, directly suppresses CD8^+^ T cell activity while steering monocyte differentiation toward anti‐inflammatory IL‐10^+^ macrophages, thus constraining cytotoxic and antitumor T cell responses [375]. Additionally, in inflammatory conditions, GABA has been observed to inhibit the secretion of Th1 and Th2 cytokines by CD4^+^ T cells, thereby impeding T cell differentiation. However, these results require further validation within tumor settings [377].

Currently, the role of γ‐aminobutyric acid (GABA) in tumor immunity remains highly controversial. It inhibits T cell activity through GABA_A/GABA_B receptors and promotes Treg cell function to drive immune escape, but in certain tumors, it exhibits dual potential of directly inhibiting tumor proliferation or inducing tolerance. This contradictory effect is highly dependent on tumor location, microenvironment components, GABA concentration gradients, and receptor heterogeneity, hindering the establishment of a universal theory. The research difficulty stems from the complexity of the neuro‐immune–tumor network‐GABA sources are diverse (tumor autocrine/neuronal release/microbiome metabolism) and interact with glutamate signals, cytokines, and metabolites, making it difficult to interpret the specific immune effects in the body. Key deficiencies include the dynamic expression of receptor mechanisms in immune subsets (such as macrophages, MDSC, NK cells) and downstream metabolic pathways that have not been clarified; existing models are unable to simulate the spatiotemporal heterogeneity of neural control and the three‐dimensional microenvironment structure; the lack of longitudinal studies on the dynamic remodeling of the GABA pathway during tumor progression or treatment; and the challenges of clinical translation, including dose balance, CNS toxicity risks, and the absence of local delivery strategies. In the future, it is necessary to analyze the microenvironment‐specific logic in the spatiotemporal dimension, develop biomimetic models and neurotoxicity avoidance strategies.

Overall, GABA can inhibit antitumor immune responses, a function linked to receptor activation and metabolic alterations. On one hand, GABA intrinsically suppresses immune cell infiltration and promotes tumor proliferation. On the other, it influences immune cell behavior by activating different GABA receptor subtypes. Currently, the complexity of GABA's role within the TME eludes a unified conclusion.

Therefore, a deeper exploration of the specific roles of abnormally expressed GABA receptors in the TME is crucial for advancing tumor prediction and therapy. Drugs targeting GABA receptors are under development, but their efficacy faces challenges due to the variable roles of GABA at different disease stages. The development of antibodies targeting specific subunits of GABA receptors highly expressed in tumors presents a novel avenue for cancer treatment.

##### 4.1.1.3 Serotonergic Signaling

5‐HT, a crucial monoamine neurotransmitter, plays a significant role in regulating neural and physiological functions within both the CNS and peripheral tissues [378]. It influences neural activity by engaging with 5‐HT receptors (5‐HTRs) across various brain regions, thereby affecting mood, cognition, and sleep. Recently, the involvement of 5‐HT in antitumor immunity has drawn heightened interest. Research suggests that 5‐HT promotes tumor progression and exerts its biological effects by interacting with immune cells in the TME [379]. Through a complex, multistep mechanism, 5‐HT and its metabolites orchestrate key biological processes, including tumor cell proliferation, angiogenesis, and immune regulation, thus playing a crucial role in tumorigenesis and progression [380].

The presence of 5‐HTRs on tumor and immune cells alike allows these cells to respond dynamically to 5‐HT's regulatory effects. In lung adenocarcinoma, the activation of the 5‐HT1A receptor initiates the pSTAT3 and autophagy pathways, resulting in increased PD‐L1 expression in tumor cells and fostering an immunosuppressive microenvironment [381]. Additionally, 5‐HT reduces the antiangiogenic activity of MMP12 by downregulating its expression and potentially enhances angiogenesis by modulating vascular endothelial cadherin expression in TAM [382]. In macrophages, 5‐HT promotes the development of anti‐inflammatory M2 macrophages through 5‐HT2B and 5‐HT7 receptors, thereby supporting tumor progression [383, 384]. Moreover, 5‐HT influences tumor‐associated fibroblasts, encouraging macrophage polarization toward the M2 phenotype and thus weakening the antitumor immune response [385]. On the T‐cell front, 5‐HT impacts differentiation and function via the 5‐HT1A receptor. Specifically, 5‐HT‐stimulated DCs ramp up the production of the Th2 chemokine CCL22 while reducing Th1 chemokine CXCL10, leading to the polarization of naive T cells toward a Th2 phenotype and diminishing CTL activity [386]. Owing to the tumor‐promoting actions of 5‐HT, current therapeutic research is probing strategies to curb tumor growth by targeting 5‐HT levels across various models, to enhance CD8^+^ T cell infiltration in the TME and reduce PD‐L1 expression [387]. Although current evidence highlights the role of 5‐HT in promoting tumor progression via the suppression of antitumor immune responses, its precise influence within the tumor immune microenvironment remains insufficiently elucidated. Recent research suggests that 5‐HT may enhance glycolytic metabolism and strengthen CD8^+^ T cell‐mediated antitumor immune responses [379]. This critical disparity is likely attributable to the role of posttranslational modifications. Specifically, 5‐HT can be covalently conjugated to glutamine residues on target proteins via a catalytic process mediated by tissue transglutaminase 2, resulting in a modification known as serotonylation. This process directly modulates the function of the target proteins. In contrast to the rapid and transient receptor‐mediated effects characterized by Ca^2^⁺ signaling, serotonylation provides a sustained and stable activation signal that contributes to the long‐term maintenance of CD8⁺ T cell activation. Notably, activated CD8⁺ T cells can enhance both the synthesis and uptake of 5‐HT by upregulating tryptophan hydroxylase 1 (TPH1) and the 5‐HT transporter (SERT), while also increasing the expression of monoamine oxidase A to degrade 5‐HT. This coordinated regulation forms a self‐tuning metabolic network that precisely controls intracellular 5‐HT levels, thereby preventing potential adverse effects caused by overactivation. On the other hand, the source of 5‐HT within the TME is highly complex: it may be released in large quantities by tumor cells and platelets, or accumulated through endogenous synthesis mediated by TPH1 in CD8⁺T cells themselves and via SERT‐dependent uptake from the extracellular milieu [379]. Dynamic changes in local 5‐HT concentration directly influence its immunomodulatory effects—low concentrations tend to activate high‐affinity receptors such as 5‐HT1A, whereas high concentrations may further stimulate low‐affinity receptors and trigger nonreceptor‐dependent cellular responses.

Currently, there is a fundamental controversy regarding the role of 5‐HT in tumor immunity: it drives immunosuppression through specific receptors, but in certain circumstances it enhances the antitumor activity of CD8⁺ T cells. This dual contradictory effect of promoting tumor growth and antitumor response is highly dependent on the heterogeneity of time and space such as tumor type and microenvironment, and it is difficult to establish a unified regulatory logic. Moreover, the dual sources of 5‐HT, dynamic metabolism within the tumor, and interaction with other pathways make the interpretation of specific immune effects within the body challenging. Key deficiencies include: the expression profiles, heterodimerization, and nonclassical pathways of the 14 5‐HTRs in immune subgroups have not been clarified; there is a lack of longitudinal studies on the dynamic remodeling of the 5‐HT pathway during tumor progression or immunotherapy; the clinical translation lacks an unclear dose threshold, off‐target risk of selective 5‐HT reuptake inhibitors (SSRIs), and absence of local delivery strategies.

Future research should resolve the interaction network between neurotransmitter receptors and immune cells at the single‐cell resolution, construct a dynamic spatiotemporal model, and design specific intervention strategies targeting the TME. Further studies are imperative to delineate the effects of targeting 5‐HT across different cancer types, thereby deepening our understanding of its functions in tumor immunology. Such insights will be foundational in the development of innovative therapeutic strategies.

##### 4.1.1.4 Adrenergic Signaling

Epi and NE, pivotal neurotransmitters within the adrenergic system, are essential for cardiovascular modulation and smooth muscle regulation, being released by the SNS. Recent investigations have elucidated the profound influence of adrenergic signaling on immune cell functionality via ARs expressed across diverse immune cell populations [388]. These receptors, categorized into α (α_1_, α_2_) and β (β_1_, β_2_, β_3_) subtypes, are integral to the modulation of the TME and its associated immune dynamics [389].

In myeloid immune cells, particularly macrophages, adrenergic signaling—particularly β‐adrenergic—plays a crucial role in tumor progression by facilitating the secretion of various immunosuppressive molecules such as IL‐6, TGF‐β, VEGF, MMPs, and COX‐2 [390, 391]. Furthermore, α_2_‐AR agonists have been shown to enhance macrophage‐mediated T cell activation, further illustrating the complex role of adrenergic signaling in immune regulation [392]. β‐Adrenergic stimulation augments macrophage infiltration and cancer metastasis, a process counteracted by β‐adrenergic antagonists such as propranolol [393]. The interplay of adrenergic signaling in macrophage recruitment is underscored by NE‐induced IL‐6 production and stromal cell activation, which bolster tumor cell migration and neural invasion [394]. β_3_‐Adrenergic signaling prompts ovarian cancer cells to secrete neurotrophic factors that modulate macrophage and neutrophil polarization, thereby facilitating interactions with hematopoietic and mesenchymal stem cells [395, 396]. In breast cancer, NE fosters macrophage infiltration, directing M2 polarization and enhancing metastasis, albeit without influencing primary tumor growth [397, 398]. Stimulation with nonselective β‐adrenergic agonists such as isoproterenol upregulates ARG1 and PD‐L1 in MDSCs, thereby impairing T cell function and promoting immune evasion—an effect reversible by β‐receptor blockers [399].

In lymphoid immune cells, β‐adrenergic signaling attenuates responses in CD8^+^ T and NK cells, reducing IFN‐I expression and thereby facilitating metastasis, as corroborated by studies in breast cancer [400]. This signaling can induce lymphocyte apoptosis, reconfiguring NK cell distribution and activity to promote immune escape [401]. Moreover, adrenergic signaling modulates lymphocyte trafficking; within lymph nodes, β_2_‐adrenergic signaling impairs T cell ingress into lymphatic vessels, consequently diminishing their infiltration into the TME [402]. Activated memory CD8^+^ T cells, predominantly expressing β_2_‐AR, experience regulated effector functions within the TME through this signaling [403]. Adrenergic signaling also weakens CD8^+^ T cell efficacy against therapies such as anti‐PD‐1, markedly decreasing tumor‐free survival rates [404]. It can induce exhaustion in CD8^+^ T cells when exposed to catecholamines, with β‐adrenergic antagonists used in tandem with ICB to improve CD8^+^ T cell responses and develop TRM T cells [405, 406]. Additionally, adrenergic signaling regulates immune checkpoint expression. In breast cancer, the adrenergic influence dampens the expression of PD‐1 and Foxp3 in tumor‐infiltrating T cells when modulated by adrenergic nerve activity or β_2_‐AR inhibitors [407]. Pancreatic cancer analyses reveal NE exposure leads to decreased expression of major histocompatibility and CD80 molecules while increasing the levels of immunosuppressive indoleamine 2,3‐dioxygenase (IDO) and PD‐L1, hence preventing effective antigen recognition by T cells [408].

The role of adrenergic signaling in tumor immunity remains highly contradictory: activation of β‐AR typically induces the expansion of MDSCs, inhibits the functions of CD8⁺ T/NK cells, and upregulates PD‐L1 to promote immunosuppression; while agonism of α_2_‐AR can enhance antigen presentation by macrophages, reduce MDSCs, and activate T‐cell antitumor responses. What is more complex is that subtypes such as β_3_‐AR exhibit bidirectional effects in different tumors (for example, in renal cancer, they initially promote tumor growth but later inhibit it and exacerbate metastasis). This functional conflict is highly dependent on tumor type, receptor spatial distribution, catecholamine concentration gradients, and microenvironment heterogeneity. The difficulty in research stems from the complex entanglement of the neuro‐endocrine–immune network throughout the body—sympathetic nerve endings NE, adrenal medulla E, and stress‐induced catecholamine fluctuations interact hierarchically with local cytokines, metabolic reprogramming, and immune checkpoint formation, making it difficult to interpret specific immune effects in vivo. Key deficiencies include: the dynamic expression of the nine AR subtypes in T/NK/DCs and the heterogeneity of “cold” and “hot” tumor functions is unknown; animal models are difficult to simulate the anatomical specificity of sympathetic nerve innervation and systemic stress fluctuations, resulting in a disconnect between preclinical and clinical results (for example, β‐blockers are effective in mice but the clinical survival benefit is not proven); the lack of dynamic tracking of pathways during tumor progression or ICB treatment; and the challenges in clinical translation due to the risks of cardiovascular effects of β‐blockers, the toxicity of α_2_‐AR agonists at extremely high doses, the lack of local delivery, and the dilemma of receptor subtype selectivity regulation (such as the immunosuppressive effect of β_2_‐AR, the metastasis‐promoting effect of β_3_‐AR).

These insights into adrenergic signaling and immune response interconnections underscore the critical nexus between the adrenergic system and antitumor immunity. Understanding these cancer‐specific dynamics promises to enhance the design and success of immunotherapies by harnessing adrenergic pathways to overcome therapeutic resistance, offering a refined approach to augment cancer treatment efficacy.

##### 4.1.1.5 Cholinergic Signaling

Ach, a primary neurotransmitter of the PNS, originates from neuronal and non‐neuronal sources. These include epithelial, mesothelial, endothelial, immune, and malignant cells. Beyond its CNS role, Ach also plays a critical part in peripheral organ function, where cholinergic nerves modulate tumor immunity by acting on specific cholinergic receptors found on immune cells [409]. These receptors are divided into nicotinic (nAChRs) and muscarinic (mAChR) types [410]. The vagus nerve, a significant source of Ach, influences tumor progression by affecting cancer cell proliferation and the TME through these receptors [167].

The dynamic interaction between cholinergic signaling and immune cells, particularly myeloid and lymphoid types, significantly influences tumor immunity. Ach has been shown to inhibit tumor necrosis factor secretion via nAChRs and to promote IL‐10 production in macrophages, highlighting its immunosuppressive effects [411]. In breast cancer, TAMs with mAChR subtypes M1 and M2 activate pathways that encourage tumor angiogenesis [412]. Antigen‐presenting cells experience downregulated T‐cell differentiation due to nAChR activity, while M1 mAChR stimulation aids CD8^+^ T cells in becoming CTLs [413].

In pancreatic cancer, high Ach levels impede PDAC cells from efficiently recruiting CD8^+^ T cells by repressing CCL5 through HDAC1, thereby altering the Th1/Th2 balance and weakening antitumor responses [414]. Cholinergic vagal signals are known to boost trefoil factor 2 release from memory T cells, reducing inflammation and slowing colon cancer progression. Additionally, these signals can elevate PD‐L1 expression in MDSCs, impede IFN‐γ production and CD4^+^ T cell proliferation, thus facilitating immune evasion [415]. In lung adenocarcinoma, nAChR activation increases PD‐L1 expression via pSTAT3 and JAB1 pathways, fostering immune escape [416]. Colorectal cancers synthesize Ach, and while mAChR antagonists such as atropine do not alter Ach production, they inhibit PD‐L1 and PD‐L2 expression, impacting immunotherapy responsiveness [417]. Ach also enhances the self‐renewal of CD133^+^ thyroid cancer cells through the CD133–AKT pathway and boosts PD‐L1 expression [418].

The current role of ACh in tumor immunity is subject to much controversy, and the research faces difficulties and limitations. The main controversy lies in the contradictory effects: for macrophages, it can exert immunosuppressive effects as well as activate the tumor angiogenesis pathway; for T cells, the nicotinic receptor inhibits T cell differentiation, while the M1‐type muscarinic receptor facilitates the differentiation of CD8⁺ T cells into CTLs. These differential effects are influenced by receptor subtype distribution, tumor type, composition of immune cells in the microenvironment, and heterogeneity of the spatial concentration gradient of ACh, making it difficult to clearly define its overall role and unify the regulatory logic. The research difficulties stem from the wide sources of ACh (neurons, T cells, tumor cells, gut microbiota), its multilevel interaction with other neurotransmitters, cytokines, and metabolic pathways in the microenvironment, and its short half‐life, which makes it difficult to conduct dynamic detection in vivo. The technical bottleneck of precisely dissecting its specific immune effects lies in this. Additionally, animal models are unable to simulate the specificity of human neural innervation and the systemic metabolism of ACh, resulting in a disconnect between clinical translation (such as the effectiveness of α7‐nAChR agonists in mice but insufficient evidence in humans); the lack of dynamic tracking of the cholinergic pathway during tumor progression or PD‐1 treatment; and the absence of local delivery strategies for tumor delivery in clinical translation.

In summary, muscarinic receptors generally enhance immune responses, whereas nicotinic receptors tend to promote immune suppression. mAChRs generally enhance immune responses, whereas nAChRs tend to promote immunosuppression. This functional dichotomy is closely associated with their distinct expression patterns and signaling properties in immune cells. The α7nAChR is the most widely expressed subtype in the immune system, found on nearly all myeloid and lymphoid cells. As a high‐affinity, rapidly desensitizing ligand‐gated ion channel, it mediates Ca^2^⁺ influx within milliseconds before entering a desensitized state—a characteristic consistent with its role as a “rapid brake” on immune activation. Ca^2^⁺ influx further triggers downstream anti‐inflammatory signaling pathways: on one hand, it activates the JAK2/STAT3 axis, leading to nuclear translocation of phosphorylated STAT3, which suppresses NF‐κB transcriptional activity; on the other hand, α7nAChR activation may directly impede NF‐κB nuclear translocation. Additionally, signaling through this receptor inhibits the assembly and activation of the NLRP3 inflammasome, thereby broadly suppressing inflammatory responses. In contrast, muscarinic receptors belong to the GPCR family and mediate slower but more sustained signaling, making them suitable for amplifying cytokine cascades and sustaining proliferative signals—aligning with a “sustained acceleration” mechanism of immune enhancement. These receptors modulate immune cell function through second messenger systems (e.g., Ca^2^⁺, PKC, and cAMP): elevated intracellular calcium and PKC activation promote immune cell activation and degranulation in lymphocytes, mast cells, and others, while reduced cAMP levels alleviate suppression of immune responses, indirectly exerting proinflammatory effects. The complementary “accelerator” and “brake” mechanisms formed by these two receptor families in immune regulation reflect an evolutionarily refined division of labor aimed at maintaining immune homeostasis: mAChRs fine‐tune local immune responses to efficiently eliminate pathogens, whereas nAChRs—particularly through the CAP—serve as a central inhibitory system that prevents excessive immune activation and subsequent tissue damage. Ach plays a pivotal role in upregulating PD‐L1 and facilitating immune escape. A breadth of studies substantiates the involvement of cholinergic systems and neurotransmitters such as Ach in tumor progression, underscoring their potential significance for clinical applications. However, to realize these applications, more precise basic research is required to elucidate the mechanisms and mitigate potential side effects of drugs targeting these pathways.

##### 4.1.1.6 Histaminergic Signaling

HA is a vital biogenic amine with diverse physiological functions, acting as a neurotransmitter in the CNS and a key signaling molecule across the immune system, gastrointestinal tract, and dermal layers. The interaction between HA signaling and immune responses within tumor immunology has captured significant scholarly attention. HA modulates immune cell function via activation of distinct HA receptor subtypes—H_1_R, H_2_R, H_3_R, and H_4_R [419]. Engagement of these receptors influences a multitude of immune cell activities, modulating immune responses, controlling inflammatory processes, and inducing vasodilation, thus exerting multifaceted effects in the TME [420].

Within the TME, HA assumes a unique immunomodulatory role by interacting with its homologous receptors on immune cells. The expression of H_1_R through H_3_R and histidine decarboxylase in MDSCs underscores HA's regulatory capacity over these cells [421], particularly in modulating their survival and proliferation as well as elevating IL‐4 and IL‐13 levels by differentially managing ARG1 and iNOS expression, leading to T‐cell suppression [422]. H_2_R antagonists effectively counteract HA's impact on MDSCs, promoting their apoptosis, reducing ARG1 and iNOS expression, and diminishing MDSC accumulation, thereby enhancing antitumor immunity [423]. Moreover, HA influences tumor immunity by stimulating DC maturation. Activation of H_1_R and H_3_R during DC differentiation enhances their antigen presentation and proinflammatory cytokine production capabilities, encouraging Th1 polarization [424]. In contrast, H_2_R activation dampens antigen presentation, fosters IL‐10 production, and shifts responses toward Th2 polarization [425]. H_4_R expression in DCs further restricts Th2 responses by curtailing CCL2 and IL‐12 secretion [426].

Additionally, HA modulates the intricate Th1/Th2 and Treg cells balance within tumor tissues, affecting cytokine secretion and subsequently potentially promoting tumor proliferation [427]. Through H_2_R, HA undermines peripheral antigen tolerance by influencing Foxp3 expression in Treg cells. The H2R antagonist cimetidine destabilizes Foxp3, thereby compromising Treg cell functionality and augmenting cellular immune responses [428]. Furthermore, HA stimulates CD8^+^ T cells to secrete IL‐16, a T cell chemoattractant, an effect impeded by H_2_R antagonists [429]. B‐cells express HA receptors and secrete HA, functioning as immunomodulatory agents that reduce immunoglobulin production—a suppression counteracted by the H_2_R antagonist ranitidine [430]. Additionally, NK cells, expressing H_4_R, are drawn to HA‐mediated chemoattractant pathways. HA stimulation via H_2_R preserves NK cell activation, enhances IL‐2‐induced effector function, and supports NK cell‐mediated oncocyte destruction [431].Emerging research underscores that H_1_R inhibition may bolster ICB therapies by upregulating MHC‐I expression in pancreatic carcinoma [432].

Although the immunomodulatory potential of HA in tumor immunity has been widely reported, the role of HA has not yet reached a unified consensus. The controversy lies in the coexistence of “tumor‐promoting” and “tumor‐suppressing” conclusions in different studies: Activation of H2R can enhance NK cell activity, promote DC maturation and Th1 polarization, exerting antitumor effects; however, activation of the same receptor on Treg cell promotes immune escape; while H1R blockade can enhance the efficacy of immune checkpoints in some models. This functional contradiction stems from the dynamic interaction of receptor subtypes (such as inhibitory H1R, activating H2R/H4R), tumor types, local HA concentration, and the composition of the microenvironment. Key research deficiencies include: The dynamic expression patterns and signaling networks of the four HA receptors in immune subgroups such as T cells, macrophages, and MDSCs have not been systematically clarified; in clinical translation, H1R antagonists are effective in mice but only partially benefit some human cancer types, and the clinical translation faces a dose balance dilemma (such as H2R antagonists inhibiting Treg cell but weakening NK activity).

Thus, the multifaceted engagement of HA and its receptors significantly orchestrates the recruitment and regulation of immune cells within the TME, modulating tumor growth dynamics. These insights present a promising frontier for innovative cancer immunotherapeutic strategies. Nonetheless, the precise roles of HA and its receptors in immune modulation require comprehensive elucidation in continued research endeavors.

##### 4.1.1.7 Nociceptive Signaling

Peripheral sensory neurons, especially nociceptive ones, are central to sensing harmful stimuli and relaying these signals to the brain, thus initiating pain perception and adaptive responses. Nociceptors, the sensory fibers responsible for detecting and modulating immune and inflammatory reactions, are key players in this process [433]. Such pain‐inducing triggers encompass neuropeptides—notably CGRP and SP. These molecules modulate immune activity across inflammatory, infectious, and malignant conditions [434]. It is noteworthy that nociceptive neurons within solid tumors interact with cancer‐associated fibroblasts (CAFs) by releasing neuropeptides such as CGRP and NGF, significantly suppressing antitumor immune responses in the TME. In PDAC, this interaction downregulates IL‐15 expression in CAFs, thereby impairing NK cell infiltration and cytotoxicity, which promotes tumor progression and cancer‐related pain. Clinical data further indicate that in PDAC patients, the density of nociceptive innervation is negatively correlated with IL‐15 expression and NK cell infiltration, but positively correlated with pain intensity scores [435]. Moreover, this neuro‐immune regulatory pattern serves as an independent adverse prognostic factor for both overall survival (OS) and recurrence‐free survival.

SP, an eleven‐amino acid neurotransmitter from the tachykinin family, is active in both the CNS and PNS and affects emotional behavior [436]. It acts through NK1R, exerting various immune‐modulating effects such as promoting lymphocyte proliferation, activating phagocytes, and enhancing cytotoxic activities [437]. Moreover, the SP–NK1R pathway aids in the survival of T lymphocytes and induces macrophages to secrete proinflammatory cytokines while facilitating neutrophil chemotaxis [84]. Aprepitant, an NK1R antagonist, can modulate the oxidative states within the TME, demonstrating protective effects against oxidative stress [438]. However, the precise roles of SP and NK1R in antitumor immunity warrant further study.

CGRP, a 37‐amino‐acid neuropeptide, exists in two primary forms in humans: α‐CGRP and β‐CGRP. Its involvement in tumor immunity is increasingly recognized [439]. By interacting with its receptor complex—comprising a G protein‐coupled receptor, CLR, and RAMP1—CGRP influences immune cell functions, affecting both immune responses and inflammation [440]. CGRP can suppress cytokine expression in macrophages and DCs, thereby reducing antigen presentation effectiveness. Within the TME, sensory nerve fibers show RAMP1 expression, and CGRP activation prompts macrophages to secrete IL‐10, reduces effector T cells, and increases exhausted CD8^+^ T cells [441, 442]. Targeting the CGRP receptor RAMP1 or TRPV1 significantly mitigates sensory signaling, decreases T cell exhaustion, curtails tumor growth, and extends survival in experimental models [443].

Presently, there is still significant controversy regarding the role of peripheral sensory nerve signals in tumor immunity. The core disagreement centers on whether the same neuropeptide enhances or weakens antitumor immunity in different tumor types or disease stages. For example, CGRP has been shown to inhibit DC maturation and promote T cell exhaustion in some models, while in other studies, it was observed to activate effector T cells through an indirect pathway, resulting in contradictory results; similarly, the SP‐NK1R axis has been reported to both enhance the proinflammatory function of macrophages and promote an immunosuppressive microenvironment.

In addition, targeting the sensory pathway faces dose dilemmas (such as the NK1R antagonist aprepitant can inhibit oxidative stress but high doses may block beneficial inflammatory responses), off‐target effects of systemic intervention (such as the induction of vascular dilation and constriction by CGRP antagonists), and the lack of tumor‐specific neurotransmitter regulatory strategies (such as the technological gap in focused ultrasound‐targeted nerve ablation).

In conclusion, the interactions between tumors and sensory nerve fibers are multifaceted and have significant implications for tumor progression and immune dynamics. These interactions may influence tumor behavior variably depending on cancer characteristics. Understanding how peripheral sensory neurons affect tumor growth and antitumor responses could reveal novel therapeutic targets, such as neuropeptides and their receptors. Incorporating these discoveries into existing cancer therapies could potentially enhance treatment effectiveness, offering promising avenues for future research and clinical application.

#### 4.1.2 Glial–Immune Interactions in TME

Glial cells, while not directly involved in the transmission of electrical signals, are critical components of the nervous system [444]. They provide essential support and protection to neurons, facilitate nutrient metabolism, and contribute to the maintenance of neuronal homeostasis [445]. Glial cells play an indispensable role in the formation and maintenance of the myelin sheath, a lipid structure that envelops neuronal axons and is essential for efficient and rapid transmission of nerve signals. Specifically, in the CNS, oligodendrocytes extend their plasma membranes to wrap around multiple axons in segmented intervals, forming insulated myelin segments. In the PNS, Schwann cells (SCs) are responsible for myelinating individual axons. The structure of the myelin sheath enables nerve impulses to propagate via saltatory conduction, significantly increasing conduction speed while reducing energy consumption [446]. Although the interactions between neurons and tumor immunity have been partially explored, the intricate roles of peripheral glial cells in tumor immunity await further elucidation [447].

**FIGURE 6:**
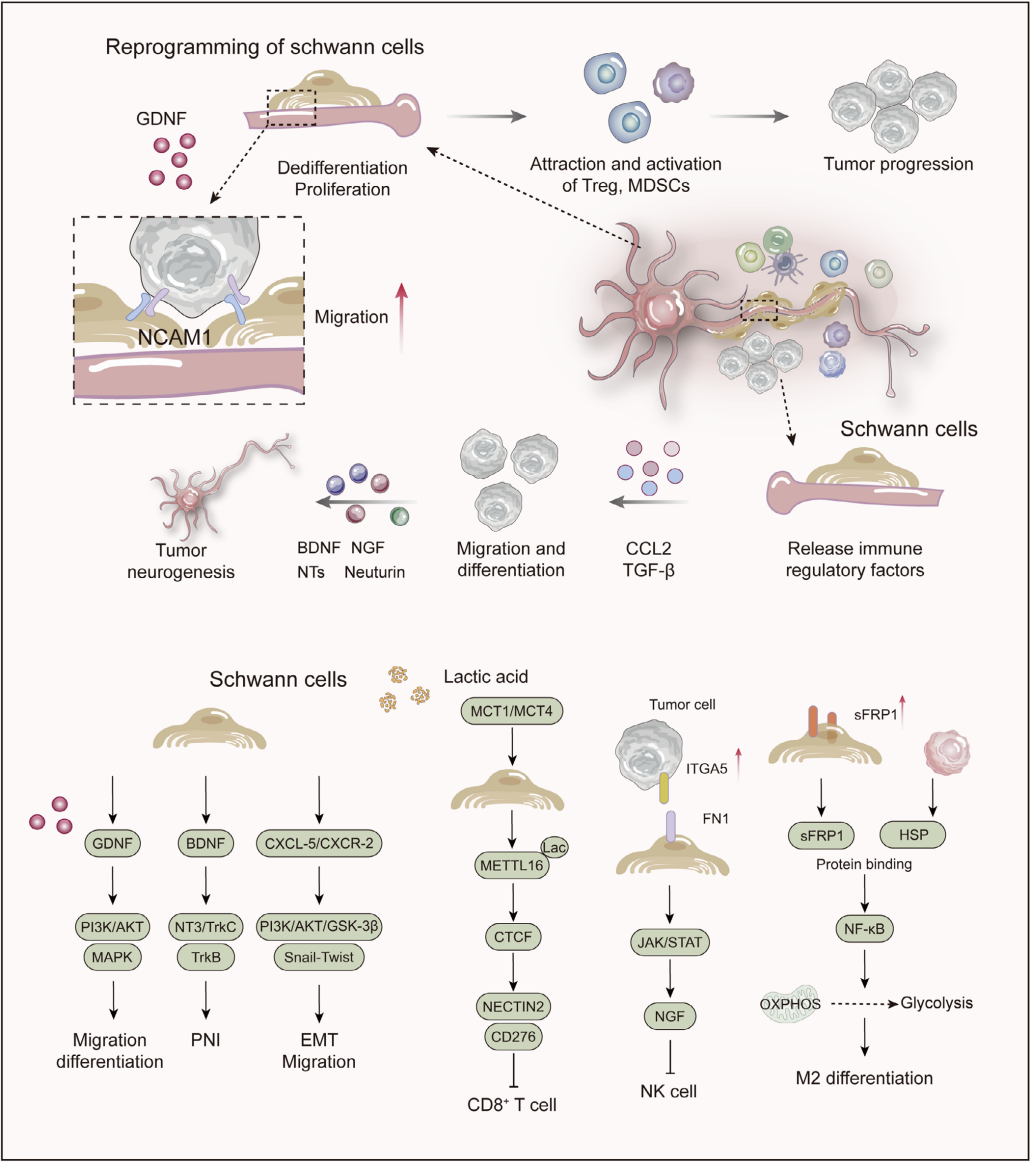
Interactions between Schwann cells and tumor cells. Schwann cells express NCAM1, which facilitates the adhesion of tumor cells and thereby promotes their migration along nerve fibers. As immune modulators, Schwann cells attract immature myeloid cells, dendritic cells, and T cells, polarizing them into immunosuppressive MDSCs and Tregs. By modulating the secretion of neurotrophic factors—such as NGF and BDNF—from tumor cells, Schwann cells activate multiple signaling pathways including PI3K/AKT and JAK/STAT, thereby suppressing immune cell function and exacerbating the microinvasive behavior of tumor cells. Additionally, lactate accumulation within the tumor microenvironment induces lactylation of METTL16 protein, which in turn promotes the expression of downstream immunosuppressive molecules such as CD276 and NECTIN2. *Abbreviations*: NCAM1, neural cell adhesion molecule 1; MDSCs, myeloid‐derived suppressor cells; Tregs, regulatory T cells; NGF, nerve growth factor; BDNF, brain‐derived neurotrophic factor.

SCs, the predominant glial cell type within the PNS, have recently garnered attention for their significant involvement in tumorigenesis [448] (Figure 6). Studies have shown that SCs can specifically activate receptor tyrosine kinase family members on the surface of tumor cells (including the RET, TrkA, TrkB) by secreting GDNF, NGF, and BDNF [449]. GDNF activation subsequently induces chemotactic migration of tumor cells through the PI3K/AKT and MAPK signaling pathways. The BDNF/TrkB axis, along with NT‐3/TrkC signaling, promotes PDAC progression through perineural invasion. Compared with normal tissues, TrkB is overexpressed in approximately 50% of PDAC cases [450]. Furthermore, elevated SCs expression of neural cell adhesion molecule (NCAM) and L1 cell adhesion molecule (L1CAM) facilitates calcium‐dependent cell‐to‐cell binding. This markedly strengthens malignant cell–nerve bundle adhesion, creating a physical foundation for PNI [451]. SCs drive epithelial–mesenchymal transition (EMT) and enhance metastatic potential in lung cancer through pathways involving CXCL‐5/CXCR‐2/PI3K/AKT and GSK‐3β/Snail–Twist, demonstrating distinct tumor‐promoting behavior [452]. Within the TME, hypoxia and lactate accumulation synergistically promote immune evasion and tumor progression. Lactate enters tumor‐associated SCs (TASCs) via monocarboxylate transporters MCT1/MCT4, binds to METTL16, and induces lactylation at lysine 269, thereby enhancing m6A‐dependent CTCF protein stability and facilitating the transcriptional activation of immunosuppressive ligands. This epigenetic reprogramming mechanism significantly upregulates immune checkpoint molecules such as CD276 and NECTIN2, which subsequently inhibit CD8⁺ T cell function and accelerate malignant progression in PDAC [453]. Furthermore, across multiple cancer models, SCs interact with tumor cells exhibiting high ITGA5 expression, leading to marked upregulation of GFAP and c‐Jun, indicative of a transition toward a proliferative and nerve‐repair phenotype. In this process, ITGA5 directly binds to fibronectin (FN1) secreted by SCs, activating the JAK–STAT signaling pathway to further promote SC proliferation and secretion of NGF. Elevated NGF levels significantly suppress the cytotoxicity of NK cells, thereby facilitating immune escape and supporting tumor growth [454]. Following nerve injury, SCs possess the remarkable ability to undergo dedifferentiation, re‐enter the cell cycle, and initiate a process known as SCs reprogramming. This reprogramming facilitates the secretion of chemokines that enhance the chemotactic migration of immune cells [455]. Studies employing coculture models of SCs with cervical cancer cell lines, such as HeLa and ME180, have demonstrated that SCs secrete the chemokine CCL2, impacting the immune landscape in the TME [456]. CCL2 plays a crucial role in regulating immune suppression in the TME and is closely linked to poor prognoses in cervical cancer patients. Additionally, CCL2 recruits TAMs and activates the TGF‐β/Smad signaling axis, promoting their polarization toward the M2 phenotype and thereby reducing antitumor immune activity [457]. Following nerve injury, SCs highly express secreted frizzled‐related protein 1 (sFRP1). sFRP1 interacts with heat shock proteins in macrophages, driving NF‐κB transcription and promoting a metabolic shift in macrophages from OXPHOS to aerobic glycolysis [458]. A reciprocal bFGF/IL‐33 signaling axis between SCs and TAMs has also been found to play a key role in promoting PNI in PDAC, thereby establishing a self‐amplifying feedback loop that drives neural infiltration [459]. SCs assist in drawing and activating MDSCs within the TME, thereby facilitating the advancement of melanoma, pancreatic, and prostate cancers [457, 460, 461].

Research on nerve injury has revealed that SCs express TLRs, which detect alarmins secreted by various cancer cells, including those of pancreatic, breast, and colon origins, as well as glioblastoma (GBM) multiforme [462]. TLRs on macrophages and DCs within the TME perceive these alarmins, enhancing innate immune responses and facilitating antigen presentation and T cell activation [463, 464]. This suggests a potential role for SCs as antigen‐presenting entities. The expression of markers such as CD74, CD1a, and cell adhesion proteins on SCs further supports their potential antigen‐presenting function, although further investigation is warranted [465, 466, 467]. However, SCs can also epigenetically reprogram TASCs, leading to the production of immunosuppressive molecules such as CD276 and NECTIN2. This process impairs CD8⁺ T cell function and promotes resistance to anti‐PD‐1 therapy [453].

The role of SCs in tumor immunity is highly controversial and the research faces numerous difficulties and shortcomings. On one hand, SCs promote M2‐type macrophage polarization and the recruitment of MDSCs by secreting CCL2, thereby driving immunosuppression. On the other hand, SCs express TLRs to recognize tumor alarm signals and carry antigen‐presenting markers such as CD74/CD1a, which may participate in innate immune activation and antigen presentation. The functional contradiction leads to the difficulty in defining the core role. The research difficulties stem from the complexity of the interaction between SCs, tumors, and immunity. The specific effects of the various factors secreted by them (such as GDNF, NGF, chemokines, etc.) in different tumor types and microenvironments have not been clearly identified. Moreover, the mechanism of the dedifferentiation reprogramming process on immune regulation lacks in‐depth analysis. Additionally, current research mostly relies on cell coculture models or limited observations of specific tumor types, lacking systematic in vivo studies for verification, especially the lack of direct evidence for their antigen‐presenting function. At the same time, the differences between animal models and human TMEs also lead to significant obstacles in the translation of research results to clinical applications. More realistic research models and more precise mechanism exploration are urgently needed to promote the progress of related research.

In conclusion, SCs exhibit multifaceted roles within the TME, akin to their response to nerve injury. They release a spectrum of chemokines and cytokines that orchestrate immune cell recruitment and modulation, thereby fostering an immunosuppressive environment that supports tumor progression. While direct evidence of their antigen‐presenting capacity in the context of tumor immunity is currently inconclusive, this remains an intriguing avenue for future research. A deeper understanding of SC interactions with tumor and immune cells will shed light on their influence on tumor growth, invasion, and metastasis, and may identify novel avenues for targeting cancer therapeutically by manipulating SCs functions.

### 4.2 CNS Tumors

Malignant tumors of the CNS are primarily divided into two categories: one is primary tumors originating from brain parenchyma or meninges (e.g., gliomas, medulloblastomas), and the other is brain metastases (BrM) formed by the metastasis of extracranial malignancies (e.g., lung cancer, breast cancer, melanoma) to the brain [468]. The brain microenvironment (BME), serving as the niche for tumor growth, is characterized by the presence of highly heterogeneous immune cell populations, including resident microglia, peripherally recruited macrophages, T cells, neutrophils, and others [469]. These cells participate in tumor immunoediting through complex signaling networks. Although traditional views held that the physical barrier function of the BBB rendered the brain “immune‐privileged,” recent research confirms that the BBB is not an absolute barrier: under physiological conditions, it regulates neuro‐immune communication (e.g., meningeal lymphatic vessel‐mediated immune surveillance), while under pathological conditions, it actively participates in immune responses [470]. Neuro‐immune interactions play a central role in maintaining brain homeostasis and driving neuroinflammation, completely overturning previous understanding [22]. This dynamic immune interplay is particularly prominent in brain tumors. When the BBB is disrupted due to aberrant tumor angiogenesis or erosion by inflammatory factors, vascular permeability significantly increases, causing the peripheral immune cells (e.g., monocytes, neutrophils, T cells) to massively infiltrate the CNS, forming an immunosuppressive microenvironment [471]. Notably, fundamental differences exist in the immune microenvironments of primary brain tumors (e.g., gliomas) and BrM.

#### 4.2.1 Glioma: Neuronal Activity‐Driven Immunosuppression

Gliomas are the most common primary CNS tumors, originating from neuroepithelial tissue [468]. Specifically, they refer to tumors arising from glial cells. Their progression involves significant remodeling of the TME, characterized particularly by BBB disruption and an immunosuppressive microenvironment [469, 472]. This immunosuppressive nature constitutes a central therapeutic challenge, limiting the efficacy of combined interventions—including surgical resection, radiotherapy, temozolomide chemotherapy, and emerging immunotherapies—resulting in poor patient prognosis [473, 474]. Recent research emphasizes that the immunosuppressive mechanisms in gliomas are closely linked to the interplay network among cellular components (e.g., malignant cells, neurons, immune cells) within the microenvironment [475, 476].

##### 4.2.1.1 Crosstalk between Glioma and Immune Cells

TAMs constitute one of the most abundant cellular populations in the BME [477], comprising two subsets defined by tissue origin: CNS‐resident TAMs and peripherally infiltrating monocyte‐derived macrophages (MDMs) [478]. The CNS‐resident TAM subset can be further subdivided into major tissue‐resident macrophages (microglia) and border‐associated macrophages [479]. Microglia, the resident macrophages of the CNS, maintain a resting state under physiological conditions, exhibiting their characteristic ramified morphology [480]. Upon exposure to pathological stimuli, microglia rapidly activate and undergo morphological remodeling, subsequently executing immune surveillance and defensive functions [145]. TAMs possess a dual regulatory role in either promoting or inhibiting tumor growth [481]. Their functional phenotype primarily depends on their polarization state and local microenvironmental factors [482]. Within the TME, glioma‐associated microglia and macrophages are recruited to the tumor site under the influence of chemokines such as CCL2, where they polarize into M2‐type macrophages [483]. This polarization process suppresses antitumor immune responses and supports tumor growth. Microglia promote tumorigenesis through CCL5 chemokine expression, stimulating oligodendrocyte precursor cell multiplication and viability [484]. Activated microglia additionally enable malignant cell dispersion and infiltration by releasing diverse signaling molecules and growth regulators, including IL‐6, TGF‐β, and VEGF [485]. In the GBM microenvironment, oncostatin M (OSM) derived from tumor‐associated macrophages (GAMs) activates STAT3 by binding to the OSMR/LIFR–GP130 receptor complex, driving mesenchymal transition of GBM cells [486]. Concurrently, integrin αvβ5 (ITGαvβ5) highly expressed on M2 macrophages interacts with its ligand osteopontin (OPN) to maintain an immunosuppressive phenotype; OPN deletion reduces M2 infiltration, enhances CD8⁺ T cell cytotoxicity, and improves survival [487]. This integrin further promotes M2 polarization and aberrant angiogenesis via the Src–PI3K–YAP pathway [488]. Additionally, GBM‐secreted SLIT2 mediates GAM chemotaxis and polarization through PI3K‐γ [489], synergizing with OPN to reinforce immunosuppression and angiogenesis [490]. EZH2 inhibition upregulates M1 markers while suppressing M2 properties in microglia and enhances their phagocytic capacity [491]. TAMs contribute to a profoundly immunosuppressive environment by secreting cytokines such as TGF‐β and IL‐10, which not only inhibit the activation of effector T cells but also promote the proliferation of Treg cell [492, 493]. Concurrently, TAMs directly suppress T cell functional activity by expressing immune checkpoint molecules, such as PD‐L1 [494]. Additionally, immunosuppressive signals secreted by both TAMs and tumor cells exacerbate T cell exhaustion, further weakening the antitumor immune response. Lactate produced by glioma cells drives the polarization of TAMs toward the immunosuppressive M2 phenotype. TAMs, in turn, release HMGB1, promoting malignant tumor behavior [495]. Lactate dehydrogenase A (LDHA) regulates macrophage behavior by modulating metabolism and signaling, thereby promoting GBM progression [496]. Consequently, LDHA and its downstream pathways represent potential therapeutic targets. Studies have shown that the inhibitory function of MDMs is closely related to an increase in glycolysis [497]. GBM induces glycolysis, lactate production, and IL‐10 secretion in MDMs [498]. IL‐10 subsequently inhibits T cells via histone lactylation [499]. Furthermore, GBM activates the PERK–ATF4 pathway in MDMs, upregulating glucose transporter 1 to enhance glycolysis [499]. Therefore, targeting PERK/ATF4 in MDMs reduces histone lactylation, increases intratumoral T cell infiltration, inhibits tumor growth, and enhances the efficacy of immunotherapy. A distinct lipid‐rich TAM subpopulation accumulates cholesterol by phagocytosing myelin fragments [500]. These TAMs then directly transfer lipids to GBM cells to fulfill their heightened metabolic demands.

As key adaptive immune cells within the glioma TME, the normal activity of T cells is frequently constrained by multiple immunosuppressive mechanisms [501]. These include tumor‐induced upregulation of immune checkpoint molecules, T cell exhaustion, and the infiltration of Treg cells. Compared with isocitrate dehydrogenase (IDH)‐mutant gliomas, T cell infiltration is more pronounced in IDH‐wildtype gliomas [502, 503]. However, most T cells infiltrating the tumor site exist in a state of functional exhaustion, primarily due to persistent tumor antigen stimulation. Exhausted T cells exhibit significant dysfunction, characterized by reduced proliferative capacity, impaired effector function, and increased expression of inhibitory receptors such as PD‐1 and CTLA‐4 [504]. Furthermore, the presence of Treg cells exacerbates the immunosuppressive milieu within GBM [505]. Although effector T cells possess the potential to recognize and eliminate tumor cells, their function is often profoundly suppressed within the GBM microenvironment. This suppression stems from factors including the upregulation of immune checkpoint molecules and the accumulation of immunosuppressive cells [506].

Within the immune cell composition of GBM, B cells constitute a relatively low proportion. However, they play a significant and non‐negligible role in tumor development, progression, and the body's response to treatment [507]. B cells in GBM primarily consist of two major functional subsets: regulatory B cells with immunosuppressive functions, and B cells capable of acting as antigen‐presenting cells to promote T cell proliferation and activation [508]. B cells can induce the formation of an immunosuppressive environment and promote angiogenesis by secreting cytokines such as IL‐10 and TGF‐β, while simultaneously suppressing the activity of T cells and NK cells, thereby driving tumor invasion within brain tissue [509]. Additionally, B cells secrete various proangiogenic factors, including VEGF, stromal cell‐derived factor‐1 (CXCL12), and B‐lymphocyte chemoattractant (CXCL13), stimulating the formation of new blood vessels to provide essential nutrients and oxygen support for tumor growth [510]. Recent studies have shown that anti‐αVβ8 blockers targeting the TGF‐β signaling pathway can affect antitumor immunity through multiple mechanisms [511]. The research found that combining this therapy with PD‐1 inhibitors can restore B‐cell function, and B‐cell immunity in the TME is crucial for enhancing the efficacy of immunotherapy against brain tumors, providing a new basis for combined immunotherapy strategies for CNS tumors.

MDSCs comprise a diverse group of immature myeloid elements that inhibit immune effector functionality via multiple pathways [512]. Research indicates MDSCs exhibit notable expansion in GBM patient peripheral blood and tumor sites [513]. These cells propel malignancy progression through cytokine/growth factor secretion, immune effector suppression, and angiogenesis stimulation [514]. Notably, STAT3 pathway activation enables MDSC‐mediated IL‐6 release, accelerating neoplastic cell multiplication and infiltration [515]. By elevating PD‐L1 expression, MDSCs impair NK and T lymphocyte function, intensifying the TME's immunosuppressive condition [512]. Furthermore, MDSCs mediate vasculogenesis through VEGF secretion to nourish tumors, while MMP release breaks down extracellular matrices to enable tumor dissemination [516].

As crucial innate immune effector cells, NK cells extensively infiltrate the GBM microenvironment, yet their function is often severely suppressed [517]. Malignant cells compromise NK cells functionality via diverse pathways. These encompass diminished expression of activating ligands and elevated levels of inhibitory ligands, ultimately hindering NK cells’ capacity to efficiently detect and eradicate tumor cells [518]. Furthermore, immunosuppressive factors in the TME, such as TGF‐β and IL‐10, further restrict NK cell activity and promote tumor immune escape [519]. Despite these challenges, NK cells retain significant antitumor potential: they can identify and eliminate therapy‐resistant GBM stem‐like cells, and potentiate antitumor immune responses through antibody‐dependent cell‐mediated cytotoxicity [520].

As essential antigen‐presenting elements, DCs launch antitumor immunity through tumor antigen uptake, processing, and presentation. They establish two‐way signaling with malignant cells via cytokines/chemokines, regulating neoplastic expansion and viability. For example, glioma cells elevate thrombospondin‐1 (TSP‐1) expression in DCs, hindering their maturation and polarizing cytokine output toward a Th2 pattern, consequently promoting immunosuppression. Capitalizing on DCs’ immune‐activating potential, DC vaccines (such as DCVax‐L) have been engineered to amplify antitumor immune reactions against brain malignancies [521].

In conclusion, within the GBM TME, tumor cells suppress T cell activation and function by overexpressing PD‐L1, which binds to PD‐1 on effector T cells. This interaction not only induces T cell exhaustion but also significantly impairs the immune system's surveillance capacity against tumor cells. Furthermore, immunosuppressive factors (e.g., TGF‐β, IL‐10) and immunosuppressive cells (e.g., TAMs, Treg cells) within the TME amplify the PD‐1/PD‐L1 axis, collectively establishing a complex and efficient immune escape network.

The core controversy currently faced in the research on glioma–glia cell crosstalk lies in the contradiction of the role of the immune microenvironment: The dynamic balance mechanism between microglia and macrophages in tumor‐promoting polarization (such as TGF‐β‐mediated angiogenesis) and potential antitumor functions (such as antigen presentation) has not been clarified. At the same time, there are differences in the causes of spatial heterogeneity of T cells’ exhaustion in the tumor core and activation in the periphery. The main difficulties in the research stem from technical bottlenecks and system complexity—the dynamic transformation pathways of immune cells revealed by single‐cell technology are difficult to reconstruct in vitro, the BBB's phased regulation of immune infiltration increases the difficulty of mechanism interpretation, and the interaction between tumor genetic background (such as IDH mutation status) and individual immune status further raises the threshold for research reproducibility. The key deficiencies lie in the clinical translation dimension: The efficiency and predictability of immune therapy modification in “cold tumors” microenvironment are low, and there are no predictive biomarkers. Animal models cannot simulate the entire cycle of human immune editing, and the scarcity of cross‐scale integrated research leads to insufficient analysis of the three‐way network of neurons, tumors, and immune cells. The future breakthrough directions should focus on spatial multiomics technology to analyze the spatiotemporal dynamics of the microenvironment, develop biomimetic organoid models to simulate immune metabolic reprogramming, and establish a treatment response prediction framework through AI integrating multiomics data; at the same time, explore combined intervention strategies targeting immune suppression metabolism and synapses between neurons and glioma, promoting individualized immune reprogramming therapy into clinical practice.

##### 4.2.1.2 Neuron‐Immune Cell–Glioma Cell Crosstalk

Studies in Nf1‐mutant optic pathway glioma mouse models demonstrate that visual circuit activity modulates tumor growth by integrating immune responses [522]. Complementing optogenetically regulated tumor control, neuronal *Nf1* mutations heighten spontaneous action potential discharge rates. This pathological hyperactivity prompts midkine (MDK) [523]—a heparin‐binding growth factor—to stimulate T lymphocyte CCL4 secretion, triggering subsequent microglial CCL5 release; a pivotal mitogenic stimulus for glioma proliferation [524]. This research establishes a “neuron‐immune crosstalk promotes tumor progression” paradigm; however, this field remains underexplored and warrants further investigation.

##### 4.2.1.3 Crosstalk between Glioma and Neurons

Recent studies have highlighted interactions between GBM and neurons [525]. Neuron activity‐driven GBM growth is modulated by specific factors, including the synaptic adhesion molecule neuroligin‐3 [526], BDNF [527], glutamate receptor‐mediated neuron‐astrocyte interplay [528], dopaminergic receptors (D2/D4 subtypes) [529], and GABA receptors [530]. While this study did not examine immune cells' direct role in disease mechanisms, their indirect influence remains plausible. Neuron‐tumor interactions encompass multiple mechanistic pathways, which this review will not delve into due to its focus on the neuro‐immune axis.

#### 4.2.2 Metastatic Brain Tumors: BBB Breach and Immune Infiltration

BrM occur in approximately 25% of adult cancer patients, representing the most common intracranial tumors in adults with the highest incidence in non‐small cell lung cancer (NSCLC), breast cancer, and melanoma [531]. Despite comprehensive management including surgical resection, radiotherapy, and pharmacotherapy, median patient survival rarely exceeds 2 years [532]. Beyond cancer cell‐intrinsic mechanisms, TME composed of stromal and immune cells has become a research focal point for both systemic and CNS malignancies. Multiple studies confirm that the brain metastasis microenvironment (brain–MME) is essential for disseminated cancer cell colonization, whose successful establishment depends on tumor cell–immune–stromal tripartite interactions [533]. The heterogeneity and immunosuppressive nature of this microenvironment correlate with therapeutic failure and serve as key prognostic indicators for BrM patients [534].

##### 4.2.2.1 Mechanisms of BBB Disruption

The occurrence of BrM in cancer is a process involving multiple complex steps. First, tumor cells that have undergone EMT detach from the primary cancer, enter the bloodstream through intravasation to become circulating tumor cells (CTCs) [535, 536]. After hematogenous dissemination to the cerebral microvasculature, they penetrate the BBB composed of brain microvascular endothelial cells, astrocytes, pericytes, and the basement membrane, and finally adapt to the BME and colonize in the brain niche [537]. The BBB, traditionally regarded as a protective barrier of the brain, has brain capillary endothelial cells that block the invasion of bacteria, viruses, cancer cells, and so on, through TJs [538]. However, the disruption of the BBB by tumor cells is a necessary step in the formation of BrM [539]. Tumors disrupt the integrity of the BBB and induce the formation of a highly heterogeneous tumor vasculature known as the blood–tumor barrier (BTB) [540]. Composed of capillaries within brain tumor tissues, the BTB differs significantly from the normal BBB, characterized by spatial heterogeneity in vascular permeability accompanied by abnormal activation of molecular active efflux mechanisms.

Multiple studies have shown that specific CTCs can express specific cell surface marker molecules, achieving penetration into the brain parenchyma by mediating transcellular transport processes. Live real‐time imaging observations using a mouse brain metastasis model have demonstrated that early extravasation of tumor cells and their spatial proximity to brain microvessels are key prerequisites for successful colonization [541]. When metastatic cells breach the BBB, they can damage the TJs structure formed by CLDN5, whereas inhibiting the expression of angiopoietin 2 in vascular endothelial cells can effectively alleviate this barrier damage [542]. Following entrapment within the CNS's capillary network, brain‐metastasizing cells establishing in neural tissue display diverse surface molecules such as ligands and proteases. These agents both modulate BBB permeability dynamics and enable adaptive survival mechanisms for malignant cells in the cerebral tumor niche. [543]. Studies have found that placental growth factor (PLGF), which is highly expressed in small cell lung cancer (SCLC), decomposes TJs by activating the VEGFR1–ROCK–ERK1/2 axis to promote tumor cell transendothelial migration [544]. Experiments have confirmed that inhibiting PLGF can block brain metastasis in SCLC. Breast cancer cells disrupt the BBB by destroying the dynamics of brain endothelial cells through exosomal miRNA‐181c, while the analogous mechanism in lung cancer remains to be verified [545]. Additionally, research has revealed that the mechanism of BBB disruption during BM progression lies in the downregulation of endothelial cell transporter Mfsd2a (major facilitator superfamily domain 2a), a protein specialized in mediating transmembrane transport of docosahexaenoic acid (DHA) [546]. Pathological attenuation of the TGFβ/bFGF signaling pathway in endothelial cells triggers Mfsd2a dysfunction, leading to DHA transport disorders and lipid metabolism dysregulation, suggesting that restoring DHA metabolic homeostasis may emerge as a novel therapeutic strategy to block BrM. Extravasation of CTCs exhibits a dual‐mode: dynamic crossing through microperforations in the cerebral vascular wall or direct invasion by damaging endothelial cells. Notably, the pericyte mimicry mechanism is crucial—cancer cells activate the β1 integrin–ILK–YAP/MRTF axis through L1CAM‐mediated capillary pericyte‐like diffusion to promote colonization [547]; pericyte‐like cells differentiated from CD44⁺ lung cancer stem cells enhance transendothelial migration capacity via the GPR124–Wnt7b–β‐catenin signaling [548]. Furthermore, mesothelin, highly expressed in NSCLC brain metastasis (NSCLC‐BM), activates MET through the JNK pathway to disrupt BBB integrity [549]. Cerebral metastatic cells further release protein‐cleaving molecules such as cathepsin S, compromising barrier integrity through degradation of junctional adhesion molecule JAM2 [550].

Throughout brain malignancy progression—including metastatic dissemination and intraparenchymal expansion—the BBB experiences modifications in integrity, permeability, and molecular composition [551]. While the BBB presents the principal challenge for effective neuro‐oncological treatment, microenvironmental contributions remain critical for patient outcomes. Preclinical and clinical evidence demonstrates that brain‐occupying malignant cells amplify tumor‐driving signals and survival pathways, potentially diminishing targeted therapeutic and chemotherapeutic effectiveness.

##### 4.2.2.2 Modulation by Immune Microenvironment

In BrM, TAMs primarily originate from circulating monocytes, rather than brain parenchyma‐resident microglia. Both cell types can be polarized toward a protumor phenotype under the influence of the TME. As pivotal players in the BrM pathological process, TAMs exert regulatory functions across multiple stages of the metastatic cascade. Upon initiation of metastasis, exosomes released by lung cancer cells stimulate endothelial cells to secrete DKK1, inducing microglial transformation into a prometastatic phenotype and facilitating the establishment of a metastasis‐permissive niche [552]. Similarly, breast cancer cell‐derived ANXA1 activates microglia via the STAT3 signaling pathway and guides their accumulation toward metastatic sites [553]. Following tumor cell extravasation across the BBB, microglia are recruited to metastatic foci, engage in direct contact with cancer cells, and promote tumor invasion into brain tissue in a WNT signaling‐dependent manner [554]. This process is inhibited by WNT pathway inhibitors but enhanced by LPS [555]. Brain tissue damage caused by tumor invasion activates neighboring microglia through the CXCR4 signaling axis, further augmenting tumor invasive potential. CD74‐positive microglia promote metastatic outgrowth by secreting MDK; targeted inhibition of the core regulator PI3K effectively halts TAM‐mediated tumor colonization [556].During the colonization phase, cathepsin S released by BM‐derived macrophages (MDMs) and tumor cell‐derived IL‐6 (which suppresses the anti‐inflammatory function of microglia via the JAK2/STAT3 pathway) play crucial roles [557, 558]. Elevated serum IL‐6 levels significantly correlate with increased BrM risk and poor prognosis [558]. Clinical evidence indicates that high intratumoral CD163⁺ TAM density predicts shorter patient survival [559]; in vitro experiments further confirm that microglial conditioned medium possesses tumor cell growth‐promoting capabilities [560]. TAMs exhibit a paradoxical dual role in BrM immune regulation. On one hand, microglial depletion weakens NK and T cell antitumor responses, accelerating disease progression [561]. TAM‐secreted chemokines (e.g., CCL3/4) facilitate lymphocyte infiltration into tumor sites [562]. In melanoma BrM, higher abundance of microglia and monocytes associates with reduced local recurrence and leptomeningeal involvement [477]. On the other hand, TAMs suppress T cell function, upregulate immune checkpoint molecules (e.g., PD‐L1), and recruit Treg cells by secreting galectin‐1 and cystatin C or upregulating IDO [563]. Loss of microglial CX3CR1 function promotes recruitment of immunosuppressive myeloid cells highly expressing PD‐L1 and VISTA, hindering T cell‐mediated antitumor immunity [564]. Furthermore, tumor‐derived factors (e.g., neurotrophin‐3 [565], Aβ [566]) impair microglial phagocytic activity and induce a local immune‐tolerant state. In summary, current research fully reveals the complex and multifaceted functions of TAMs in BrM, involving both promotion of tumor progression and regulation (including suppression) of antitumor immunity [533]. Although sporadic evidence suggests that resident microglia might exert some antitumor immune surveillance during BrM initiation, tumor‐intrinsic properties and the polarizing influence of the microenvironment typically distort microglia‐mediated immune signaling, redirecting it to support metastatic growth. Notably, while existing studies lean toward attributing stronger direct tumor‐promoting capabilities to infiltrating MDMs, sufficient data are currently lacking to precisely define the specific roles and relative contributions of MDMs versus microglia to BrM immunology.

T cells are the main lymphocyte population in the BrM microenvironment, composed of CD8^+^ and CD4^+^ subsets, with CD4^+^ T cells being dominant and accompanied by a high proportion of Treg cell [567]. CD8^+^ T cells exhibit a typical functional exhaustion phenotype [568]. In addition to high expression of PD‐1, immune checkpoint molecules such as TIM3, CTLA‐4, TIGIT, and LAG3 are also significantly upregulated [569], and their phenotype has an extremely low overlap rate with peripheral blood CD8^+^ T cells. In terms of spatial distribution, exhausted CD8^+^ T cells mainly accumulate in the tumor core, while memory CD8^+^ T cells are distributed in the stroma‐rich area at the tumor edge; some CD8^+^ T cell subsets simultaneously express stimulatory receptors and proliferation markers [567, 570]. BrM from different primary tumor origins show differences in T cell infiltration patterns. For example, BrM derived from SCLC have a higher occurrence rate of tumor‐infiltrating lymphocytes (TIL), and the density of CD45RO^+^ TIL is associated with improved OS in patients [571]; a population of CD8^+^ T cells expressing TOX was found in melanoma BrMs, with lower expression levels of immune checkpoint markers than those in primary tumor samples [572]. CD4^+^ T cells in BrM exhibit hyporeactive and dysfunctional phenotypes, and their subsets include Th1, Th17, cytotoxic cells, and Treg cells, among which Treg cells can promote tumor progression by inducing an immunosuppressive microenvironment [573, 574]. γδ T cells can be divided into IL‐17‐producing cell subsets and cytotoxic subsets, and their high expression is negatively correlated with patient survival [575]. Contact between T cells and microglia can induce their antigen‐presenting ability (limited to the IFN response pathway), while inflammatory TAMs can highly express chemokines that promote T cell recruitment [561]. Furthermore, the immunosuppressive phenotype of T cells can undergo adaptive changes during interaction with microglia, forming a dynamic regulatory network between the two [576]. In summary, T cells in the BrM microenvironment exhibit high heterogeneity, and their phenotype, function, and spatial distribution are regulated by both the TME and the primary tumor type. Although CD8^+^ T cells have potential antitumor activity, their exhausted state and low reactivity to nontumor antigens limit immune surveillance function; subsets such as Treg cells and γδ T cells accelerate tumor progression by promoting an immunosuppressive microenvironment.

Current evidence regarding the role of B cells in BrM remains limited. Overall, compared with T cells, B cell infiltration in the originating tumors generally exhibits lower levels. Preclinical models demonstrate that PD‐1 inhibitors promote B cell proliferation within BrM [577]; this finding is corroborated by the observed increase in B cell and plasma cell abundance in patients with melanoma undergoing immunotherapy [578]. Furthermore, autoantibody levels have been confirmed to have predictive associations with BrM risk [579]. Collectively, these observations suggest that B cell activity may contribute to BrM progression and therapeutic response; however, its specific functional significance and underlying mechanisms within BrM still require elucidation.

The core controversy in the research on neuro‐immune crosstalk of BrM lies in the spatiotemporal heterogeneity of the immune microenvironment: microglia and macrophages exhibit antitumor phagocytic activity in the early stage of metastasis, but transform into prometastatic phenotypes (such as promoting angiogenesis) in the later stage, and the mechanism regulating this phenotypic transformation remains unclear; meanwhile, T cell infiltration presents contradictory specificity among primary cancer types—BrM from melanoma often show high immune infiltration, while those from breast cancer tend to form immune deserts, and the pattern has not been clarified. The research difficulty stems from the complexity across scales: the dual‐edged sword effect of the BBB at different stages of metastasis (limiting immune infiltration in the early stage and triggering nonspecific inflammation in the late stage) is difficult to simulate dynamically; the coupling mechanism of neuronal‐tumor synapses and local immune metabolic reprogramming has not been systematically analyzed. Key deficiencies include: immune checkpoint inhibitors have weak efficacy during the intact period of the BBB and lack predictive biomarkers; animal models cannot replicate the entire cycle of human immune editing; spatial multiomics technology has not clarified the three‐way network topology of neurons, metastases, and immune cells. Future breakthroughs should first focus on developing biomimetic organ‐on‐chip chips integrating the primary lesion, BBB, and brain parenchyma units to dynamically simulate the migration process of immune cells across the barrier; second, it is necessary to use single‐cell spatial metabolomics to deeply analyze the neural–immune metabolic dialogue at the metastasis front; finally, a personalized immune reprogramming strategy based on the molecular typing of the primary cancer type should be constructed, by jointly targeting neural synapses and immune metabolic checkpoints, ultimately promoting the transformation of the treatment paradigm from passive response to active immune prevention.

## 5 Therapeutic Strategies Targeting the Neuro‐Immune Axis

**FIGURE 7:**
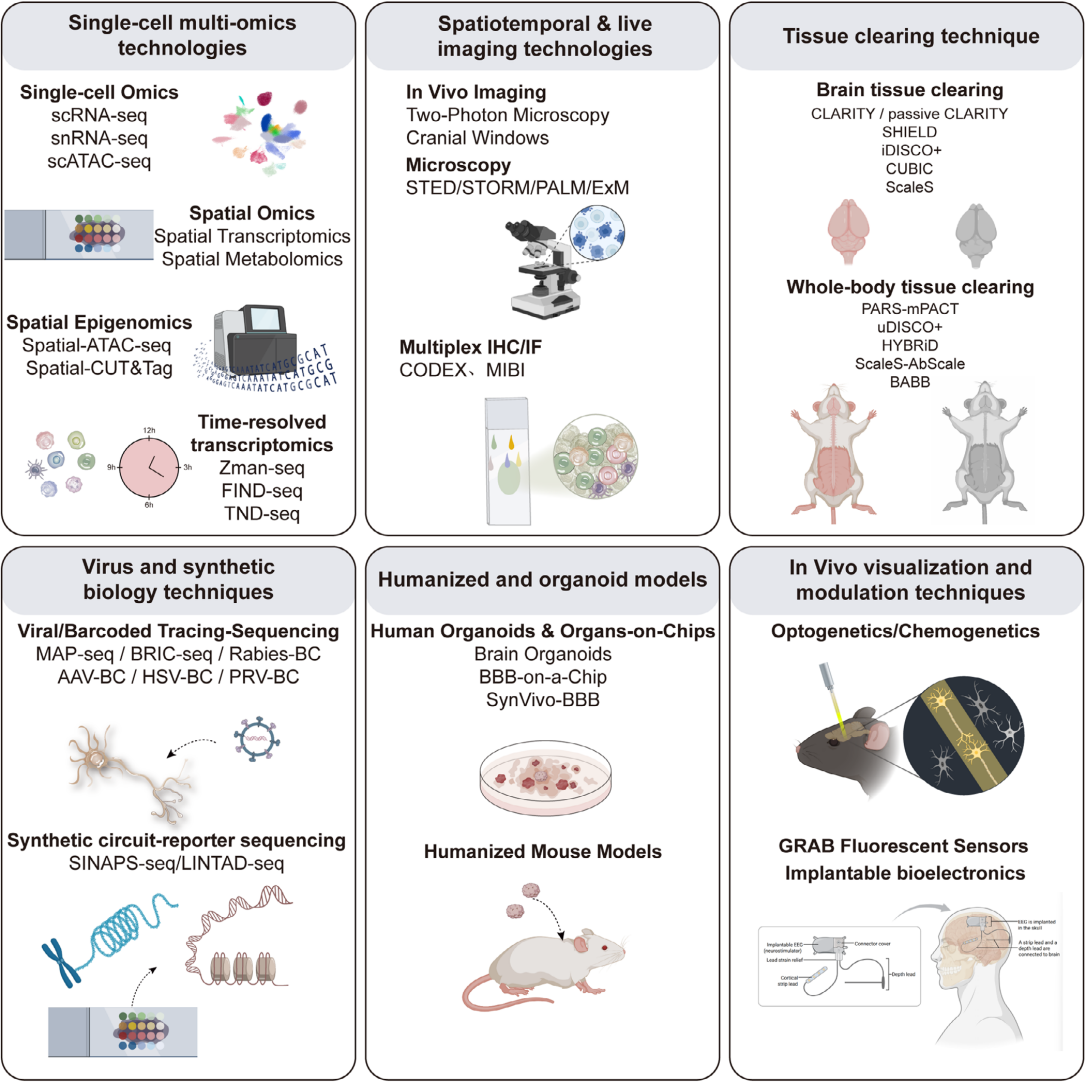
Key technologies in neuroimmunology research. Advanced technologies hold great potential for deciphering neuro‐immune interactions, yet each category possesses distinct advantages and limitations. Single‐cell multiomics enables high‐resolution dissection of cellular heterogeneity but is costly and involves complex data analysis. Spatiotemporal and intravital imaging allows real‐time dynamic monitoring of cellular activities but is constrained by limited imaging depth and phototoxicity. Tissue clearing techniques facilitate 3D visualization of thick specimens but may compromise fluorescence signals and antigen integrity. Viral and synthetic biology tools provide precise genetic manipulation capabilities yet carry risks of immunogenicity and off‐target effects. Humanized and organoid models better recapitulate human physiology but often lack authentic microenvironmental contexts and involve complex culture systems. In vivo visualization and modulation technologies offer high physiological relevance, though invasive procedures and technical challenges remain substantial obstacles.

**FIGURE 8:**
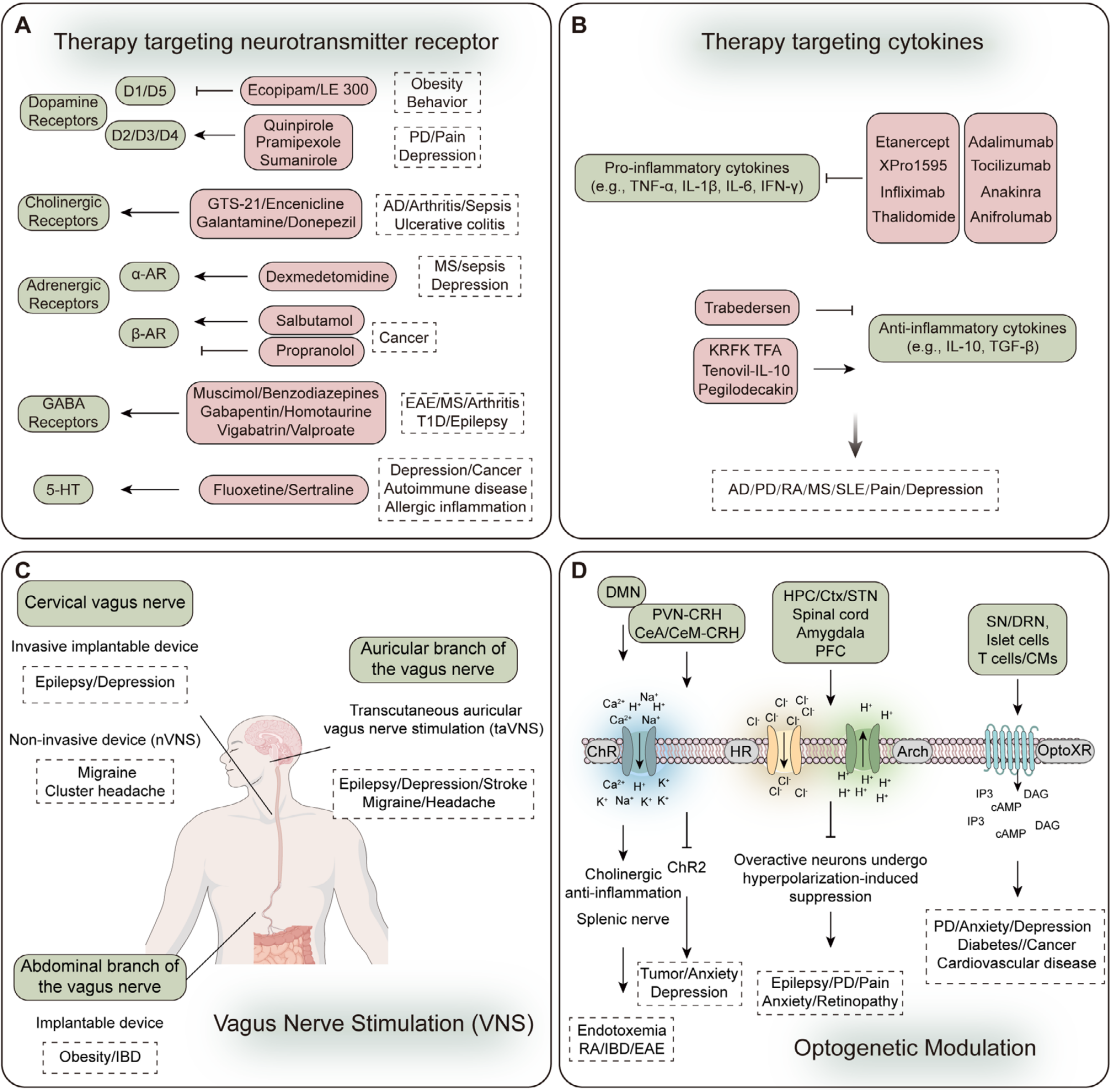
Therapeutic modulation of the neuro‐immune axis. (A and B) Major pharmacological agents targeting classical neurotransmitter receptors—including dopamine (D1/D5, D2/D3/D4), cholinergic, adrenergic (α/β‐AR), GABAergic, and serotonergic (5‐HT) receptors—as well as proinflammatory (TNF‐α, IL‐1β, IL‐6, IFN‐γ) and anti‐inflammatory (IL‐10, TGF‐β) cytokines, along with their respective therapeutic indications. (C) Two modalities of VNS: invasive implantable devices (targeting cervical/abdominal vagus nerve) and noninvasive taVNS. (D) Optogenetic regulation of neuronal activity: blue light activates ChR2, inducing sodium influx and membrane depolarization to excite neurons; yellow‐green light activates HR or Arch, leading to chloride influx or proton efflux that hyperpolarizes the membrane and inhibits neuronal firing; OptoXRs initiate downstream intracellular signaling cascades upon light activation, demonstrating therapeutic potential across various disease contexts. *Abbreviations*: VNS: vagus nerve stimulation; taVNS: transcutaneous auricular VNS; ChR2: channelrhodopsin‐2; HR: halorhodopsin; Arch: archaerhodopsin; OptoXRs: optogenetic G protein‐coupled receptors.

The diagnosis and treatment of diseases represent central components of the neuroimmunology field. Advanced technologies have not only significantly improved the diagnosis and management of neuro‐immune‐related disorders but have also demonstrated great potential in elucidating the mechanisms underlying interactions between the nervous and immune systems (Figure 7). However, each technology possesses distinct advantages and limitations, necessitating careful selection and application that is tailored to specific research questions. Single‐cell multiomics technologies (e.g., scRNA‐seq, scATAC‐seq) enable high‐resolution dissection of cellular heterogeneity and state transitions, proving particularly powerful in identifying novel cell subpopulations and rare neuro‐immune interfaces. Nevertheless, they face challenges such as high sample preparation costs, complex processing of large‐scale datasets, and the need for batch‐effect correction. Spatiotemporal omics and live imaging techniques (e.g., light‐sheet microscopy, two‐photon imaging) allow real‐time monitoring and spatial mapping of cellular dynamics, making them especially suitable for tracking immune cell migration and contact events within the CNS. However, they remain constrained by limitations in imaging depth, spatial resolution, phototoxicity, and motion artifacts. Tissue clearing methods (e.g., CLARITY, IDISCO) facilitate high‐resolution three‐dimensional imaging at the whole‐organ level, greatly advancing our understanding of neuro‐immune structural connectivity. Yet, these techniques often compromise fluorescent protein signals and native epitopes, affecting subsequent multiplex labeling and quantitative analysis. Viral vectors (e.g., adeno‐associated virus [AAV], lentiviral vectors [LVs]) and synthetic biology tools (e.g., optogenetics, chemogenetics) provide powerful means for precise genetic and circuit manipulation, enabling cell‐type‐specific functional interventions. However, issues such as immunogenicity, transfection efficiency, potential off‐target effects, and inconsistent transgene expression stability remain significant practical constraints. Humanized models and organoids excel at mimicking human physiological and pathological features, offering unique insights into human‐specific disease mechanisms and drug responses. Nonetheless, they often fail to fully recapitulate the complexity of in vivo microenvironments—including vascularization, innervation, and systemic immune circulation—and are hampered by technically demanding and variable culture systems. Although in vivo imaging and neuromodulation technologies (e.g., fluorescent sensors, implantable bioelectronics) exhibit high physiological relevance and can reflect system‐level integrated responses, their invasive nature and high technical requirements pose substantial challenges in experimental design, ethical approval, and implementation. In summary, these technological approaches collectively form a multilevel, complementary research framework. Future integration and cross‐validation of multiple technologies are expected to provide a more comprehensive and precise understanding of the molecular and cellular mechanisms underlying neuro‐immune interactions. Owing to the focus and space limitations of this article, we do not discuss the methodological details and application examples of these technologies in depth. The following section will focus on current therapeutic strategies and advances in neuro‐immune diseases (Figure 8).

### 5.1 Pharmacological Interventions

Targeting the neuro‐immune interface through pharmacological means has emerged as a promising avenue for modulating disease‐relevant immune responses. Unlike classical immunosuppressants or anti‐inflammatory drugs, these interventions leverage neurobiological pathways—particularly those involving neurotransmitter receptors and cytokine networks—to reshape immune activity in a more targeted and context‐specific manner. Two principal categories dominate current research and translational exploration: (i) agents that modulate neurotransmitter receptors expressed on immune cells, and (ii) cytokine or anticytokine therapies that indirectly influence neuro‐immune signaling by altering the inflammatory milieu. In the following sections, we examine representative therapeutic strategies from both classes, focusing on their molecular mechanisms, disease applications, and translational relevance.

#### 5.1.1 Neurotransmitter Receptor Modulators

Classical neurotransmitters in the nervous system exert profound regulatory effects on immune cell behavior. Various immune cell types—including macrophages, T cells, and DCs—express a broad range of neurotransmitter receptors, such as those for DA, ACh, NE, GABA, and 5‐HT. This expression enables neural signals or pharmacological agents to modulate key immune processes, including cytokine secretion, leukocyte proliferation, and migration [580].Targeting neurotransmitter receptors with pharmacological modulators has emerged as an important strategy to influence neuro‐immune interactions. Such approaches have found application in a wide spectrum of disorders, including neurodegenerative diseases, autoimmune conditions, psychiatric disorders, chronic pain syndromes, and even cancer. Clinical trials of drugs targeting neurotransmitter are being extensively conducted (Tables 1, 2, 3). In the following sections, we will discuss the immunomodulatory roles of major classes of neurotransmitter receptors, explore their relevance in different disease contexts, and highlight representative agonists and antagonists currently under investigation.

**TABLE 1:** Clinical trials on neurotransmitter‐targeting drugs for neurodegenerative and autoimmune diseases.

Diseases	Target	Drug/compound	Mechanism	Status	ClinicalTrials.gov identifier
Alzheimer's disease	Acetylcholinesterase inhibitors	Galantamine	Inhibit the degradation of acetylcholine and increase the level of acetylcholine in the synaptic cleft	Completed	NCT00309725
				Completed	NCT00000172
				Completed	NCT00338117
		Idalopirdine		Completed	NCT02006654
		CPC‐201		Completed	NCT02860065
		Donepezil		Completed	NCT00096473
				Completed	NCT02097056
				Terminated	NCT00843115
				Completed	NCT00566501
		GB‐5001		Completed	NCT05525780
		INM‐176		Completed	NCT01245530
		MK‐7622		Terminated	NCT01852110
		ZT‐1		Completed	NCT00423228
		Octohydroaminoacridine succinate	Simultaneously inhibiting AChE and BuChE, significantly prolonging the action time of ACh in the synaptic cleft	Unknown status	NCT03283059
		Rivastigmine		Unknown status	NCT02063269
				Completed	NCT02444637
	NMDA receptor antagonist	Memantine	Regulate glutamatergic neural transmission, reduce excitotoxicity, and protect neurons	Completed	NCT00322153
				Completed	NCT00551161
		AXS‐05		Completed	NCT04947553
	α7‐nAChR agonist	MK‐1167	Selective activation of α7‐nAChR to restore cholinergic neurotransmission	Recruiting	NCT06721156
		ABT‐126		Terminated	NCT01690195
				Completed	NCT01482845
				Completed	NCT01527916
				Completed	NCT01549834
		MT‐4666		Terminated	NCT02327182
				Completed	NCT01764243
		EVP‐6124		Completed	NCT01073228
				Terminated	NCT01969136
				Completed	NCT00766363
	5‐HT6 receptor antagonist	Intepirdine (SB‐742457)	Enhance the release of neurotransmitters such as acetylcholine and norepinephrine, and improve cognition	Completed	NCT00348192
		Idalopirdine (Lu AE58054)		Completed	NCT02006641
		Idalopirdine		Completed	NCT01955161
		Dimebon		Terminated	NCT00912288
		SUVN‐502		Completed	NCT02580305
	Dopamine receptor agonist	Rotigotine	Directly stimulate dopamine receptors and simulate the function of dopamine	Completed	NCT03250741
		R‐pramipexole		Completed	NCT01388478
		Bromocriptine		Completed	NCT04413344
	AMPA receptors agonist	CX516 (Ampalex)	Repair synaptic function and enhance neural plasticity	Completed	NCT00001662
		Tulrampator (S47445)		Completed	NCT02626572
Parkinson's disease	Dopamine receptor agonist	Rotigotine	Directly stimulate dopamine receptors and simulate the function of dopamine	Completed	NCT00296192
				Completed	NCT01723904
				Completed	NCT01646255
				Completed	NCT01523301
				Completed	NCT01646268
				Completed	NCT00505687
				Completed	NCT01782222
				Completed	NCT00243945
		LY03003		Completed	NCT02055274
				Completed	NCT04455555
		APL‐130277		Completed	NCT02228590
		Pramipexole		Completed	NCT00666653
				Completed	NCT01673724
		Bromocriptine		Completed	NCT01673724
		Piribedil		Completed	NCT01519856
				Completed	NCT01007864
				Completed	NCT00727727
		PF‐06669571		Completed	NCT02565628
		Ropinirole hydrochloride		Completed	NCT00485069
		Lisuride		Completed	NCT00408915
	Dopamine replacement therapy	Stalevo	Directly supplement the deficiency of dopamine in the brain and improve the core symptoms such as motor retardation and muscle rigidity	Completed	NCT00125567
		ND0612		Completed	NCT02577523
		Levodopa–carbidopa		Completed	NCT00141518
				Completed	NCT00558337
		HRG2010		Completed	NCT06614153
		AP09004		Completed	NCT00918177
		IPX203		Completed	NCT02271503
		RYTARY(IPX066)		Completed	NCT01096186
				Completed	NCT00880620
				Completed	NCT00974974
		Carbidopa		Completed	NCT01399905
		Crexont (IPX203)		Completed	NCT03670953
		CVT‐301		Completed	NCT02242487
				Completed	NCT01617135
				Completed	NCT02240030
				Completed	NCT02352363
				Completed	NCT01777555
				Completed	NCT03887884
		XP21279		Completed	NCT01171313
				Completed	NCT00914602
	Adenosine receptor antagonist	Istradefylline (KW‐6002)	Block the adenosine A2A receptors in the striatum and globus pallidus, and regulate the dopaminergic neurotransmission	Completed	NCT00199407
				Completed	NCT00199394
				Completed	NCT02610231
				Completed	NCT01968031
				Completed	NCT00250393
				Completed	NCT00955526
				Completed	NCT00199355
				Completed	NCT00203957
				Completed	NCT00455507
				Completed	NCT00957203
				Completed	NCT00006337
		BIIB014		Completed	NCT00438607
				Completed	NCT00442780
		SYN115		Completed	NCT01283594
	MAO‐B inhibitors	Safinamide	Inhibit the dopamine degrading enzyme (MAO‐B) to prolong the duration of dopamine action	Completed	NCT01026428
				Completed	NCT00605683
		Azilect		Completed	NCT00399477
	COMT inhibitors	BIA 9–1067	Inhibit the metabolism of levodopa in the periphery, and increase its entry into the brain and stability.	Completed	NCT01227655
				Completed	NCT01568073
		Nebicapone		Completed	NCT02763839
		Entacapone		Completed	NCT00134966
				Recruiting	NCT06236230
				Completed	NCT00373087
	Acetylcholinesterase inhibitors	Rivastigmine	Block acetylcholine receptors, correct the dopamine–acetylcholine balance, and improve tremors	Completed	NCT01519271
				Completed	NCT01856738
		Galantamine		Unknown status	NCT00211588
		Donepezil		Unknown status	NCT02450786
	NMDA receptor antagonist	Amantadine	Regulate the glutamic acid energy system to improve bradykinesia and dyskinesia	Completed	NCT01313845
				Terminated	NCT00794313
		Topiramate		Terminated	NCT00794313
		Eliprodil		Completed	NCT00001929
		Dextromethorphan		Completed	NCT00001365
Huntington's disease	VMAT2 inhibitors	Tetrabenazine	Inhibit the VMAT2, reduce the release of dopamine in the synaptic cleft, and lower the excitability of motor neurons	Completed	NCT01451463
				Completed	NCT00219804
		Valbenazine		Enrolling by invitation	NCT06312189
				Active, not recruiting	NCT04400331
				Completed	NCT04102579
		Deutetrabenazine (SD‐809)		Terminated	NCT04301726
				Completed	NCT01795859
				Recruiting	NCT04713982
	Dopamine receptor antagonists	Haloperidol	Blocking the D2 receptors in the striatum can alleviate chorea and mental behavioral symptoms.	Unknown status	NCT04071639
		Olanzapine		Completed	NCT00632645
		Risperidone		Completed	NCT04201834
	Selective serotonin reuptake inhibitors (SSRIs)	Sertraline	Increase the concentration of 5‐HT in the synaptic cleft to regulate mood and impulse control	Unknown status	NCT04071639
		Paroxetine		Completed	NCT01897896
	NMDA receptor antagonist	Amantadine	Block the excessive activation of NMDA receptors by glutamic acid and reduce neuronal death caused by calcium ion influx	Completed	NCT00001930
		Riluzole		Completed	NCT00277602
		Memantine		Completed	NCT00652457
				Completed	NCT01458470
	AMPA receptor modulator	Pridopidine	Activating the σ‐1 receptor enhances synaptic plasticity and improves the efficiency of glutamate energy transmission	Terminated	NCT02494778
				Completed	NCT02006472
				Completed	NCT00665223
				Completed	NCT01306929
				Completed	NCT04556656
				Completed	NCT00724048
Amyotrophic lateral sclerosis	Dopamine receptor agonist	R (+) Pramipexole (KNS‐760704)	Directly stimulate dopamine receptors and simulate the function of dopamine	Completed	NCT00140218
				Completed	NCT00931944
				Completed	NCT00647296
				Completed	NCT00600873
		Dexpramipexole		Completed	NCT01281189
				Terminated	NCT01622088
		Pimozide		Not yet recruiting	NCT07093268
				Unknown status	NCT03272503
	NMDA receptor antagonist	Memantine	Inhibit presynaptic voltage‐gated sodium channels to reduce glutamate release	Completed	NCT00353665
				Completed	NCT01020331
				Completed	NCT00409721
		Masitinib		Unknown status	NCT03127267
		Nuedexta		Completed	NCT03883581
				Completed	NCT01806857
		Riluzole		Terminated	NCT03679975
	Glutamic acid system modulator	Edaravone (MCI‐186)	Eliminate free radicals, block glutamate‐induced oxidative stress, and protect motor neurons	Completed	NCT00424463
				Completed	NCT00330681
				Completed	NCT00415519
				Recruiting	NCT04097158
				Completed	NCT04577404
				Completed	NCT01492686
				Unknown status	NCT03272802
				Completed	NCT04254913
				Terminated	NCT04569084
				Terminated	NCT05151471
Multiple sclerosis	Serotonin and norepinephrine reuptake inhibitors (SNRI)	Duloxetine	Regulate the serotonin and norepinephrine systems to relieve MS‐related neuralgia and anxiety	Completed	NCT00755807
		Venlafaxine		Terminated	NCT01436643
		Pregabalin		Completed	NCT00291148
	α2‐adrenergic receptor agonists	Tizanidine	Inhibit the release of hyperactive norepinephrine neurotransmitters	Completed	NCT00358293
	NMDA receptor antagonist	Ifenprodil	Inhibit glutamate release and block NMDA receptors	Not yet recruiting	NCT06330077
		Memantine		Terminated	NCT00638833
		Isoxsuprine hydrochloride		Completed	NCT03752307
		Ketamine		Completed	NCT06064162
	GABAB receptor agonist	Arbaclofen	Relieve muscle spasms and stiffness by enhancing the inhibitory effect of GABA	Completed	NCT01359566
				Completed	NCT03319732
				Completed	NCT01359566
Baclofen		Completed		NCT01743651
	Glutamic acid system modulator	Fingolimod (FTY72)	Upregulation of glutamate transporter promotes glutamate clearance and reduces excitotoxicity	Recruiting	NCT04480853
				Completed	NCT01497262
				Completed	NCT00670449
				Completed	NCT00662649
				Completed	NCT00333138

*Data source*: ClinicalTrials.gov, data as of August 16, 2025.

**TABLE 2:** Clinical trials targeting the neuro‐immune axis for chronic pain.

Diseases	Target	Drug/compound	Mechanism	Status	ClinicalTrials.gov identifier
Fibromyalgia	Serotonin and norepinephrine reuptake inhibitors (SNRI)	Duloxetine	By increasing the levels of serotonin and norepinephrine to suppress pain	Recruiting	NCT06866444
				Completed	NCT01552057
				Completed	NCT00489073
				Completed	NCT03487211
				Completed	NCT00125892
				Completed	NCT00190866
		Milnacipran		Completed	NCT01014585
				Terminated	NCT01418651
				Completed	NCT00436033
				Completed	NCT01125423
				Completed	NCT01288807
				Completed	NCT00098124
				Completed	NCT01108731
				Completed	NCT00314249
	γ‐Aminobutyric acid (GABA) analogues	Pregabalin	Inhibit the release of excitatory neurotransmitters and reduce central sensitization	Completed	NCT00645398
				Completed	NCT01226667
				Completed	NCT00830128
				Completed	NCT00282997
				Completed	NCT00230776
				Completed	NCT00830167
				Completed	NCT01271933
				Completed	NCT01387607
				Completed	NCT00151489
		Gabapentin		Completed	NCT00057278
				Unknown status	NCT05384210
	Dopamine receptor agonist	Pramipexole	It affects pain perception and mood by regulating the dopamine pathway	Terminated	NCT00689052
	Dopamine transporter antagonists	Modafinil		Completed	NCT00678691
	NMDA receptor modulator	NYX‐2925	Target NMDA receptors and correct glutamate‐mediated central sensitization	Completed	NCT03249103
				Completed	NCT04147858
	α1‐Adrenergic receptor antagonist	TNX‐102 SL	Inhibit the activation of microglia and reduce the release of IL‐6 and TNF‐α	Completed	NCT02589275
				Completed	NCT02015234
				Completed	NCT04508621
				Completed	NCT01903265
				Completed	NCT04172831
				Completed	NCT05273749
	CGRP	Galcanezumab	Neutralize CGRP ligands and inhibit pain signal transmission	Completed	NCT04158752
	TLR4	Low‐dose naltrexone	Regulate TLR4, inhibit microglial activation, and reduce IL‐6 and TNF‐α	Completed	NCT04502251
				Completed	NCT02107014
				Completed	NCT00568555
				Completed	NCT04270877
				Active, not recruiting	NCT04739995
				Withdrawn	NCT00855972
				Completed	NCT02806440
Migraine	5‐HT receptor agonists	Zolmitriptan	It inhibits neurogenic inflammation by stimulating the 5‐HT receptor to constrict the dilated cerebral blood vessels.	Completed	NCT01211145
				Completed	NCT00617695
				Completed	NCT03145467
				Completed	NCT03275922
				Completed	NCT05854992
		Sumatriptan		Not yet recruiting	NCT06868953
				Completed	NCT00488514
				Completed	NCT03156920
				Completed	NCT03542357
				Completed	NCT00963937
				Completed	NCT02856802
				Completed	NCT00240630
		Dihydroergotamine (STS101)		Completed	NCT03901482
				Completed	NCT04940390
		INP104		Completed	NCT03557333
	CGRP receptor antagonist	Rimegepant	Binding to CGRP receptors, it blocks neurogenic inflammation and vasodilation.	Completed	NCT05207865
				Completed	NCT03732638
				Completed	NCT06412965
				Completed	NCT04574362
	CGRP	Fremanezumab	Neutralize CGRP ligands and inhibit pain signal transmission	Active, not recruiting	NCT04530110
				Completed	NCT05458011
				Completed	NCT02021773
				Completed	NCT04464707
				Completed	NCT02638103
				Completed	NCT03308968
				Completed	NCT02638103
		Galcanezumab		Completed	NCT04417361
				Completed	NCT03559257
				Completed	NCT02163993
				Completed	NCT02614261
		Erenumab		Completed	NCT04465357
				Completed	NCT03867201
				Completed	NCT04252742
				Completed	NCT04152434

*Data source*: ClinicalTrials.gov, data as of August 16, 2025.

**TABLE 3:** Clinical trials targeting the neuro‐immune axis in cancer.

Diseases	Target	Drug/compound	Mechanism	Status	ClinicalTrials.gov identifier
Ovarian, fallopian tube, or primary peritoneal cancer	Dopamine receptor antagonists	ONC201	Blocking the DRD2 pathway inhibits the self‐renewal and survival signals of tumor stem cells.	Ongoing	NCT04055649
Neuroendocrine tumors				Completed	NCT03034200
Glioma				Ongoing	NCT03295396
Colorectal cancer	β‐Adrenergic antagonist	Propranolol	Block β receptors and inhibit catecholamine‐driven tumor progression	Ongoing	NCT03919461
Breast cancer				Completed	NCT02596867
Prostate cancer				Completed	NCT05679193
Non‐small cell lung cance				Ongoing	NCT05979818
Advanced solid tumors				Completed	NCT02013492
Ovarian, primary peritoneal, or fallopian tube cancer				Completed	NCT01504126
Ovarian cancer				Completed	NCT01308944
Colorectal cancer		Propranolol, COX2 inhibitor		Completed	NCT00888797
Breast cancer				Completed	NCT00502684
		Propranolol, pembrolizumab		Ongoing	NCT05741164
		Propranolol, chemotherapy		Ongoing	NCT01847001
		Propranolol		Completed	ACTRN12615000889550
Pancreatic cancer		Propranolol etodolac		Ongoing	NCT03838029
	Muscarinic agonist	Bethanechol	Activating M1/M3 receptors	Ongoing	NCT03572283
Hepatopancreabiliary tumors	β‐Adrenergic antagonist	Durvalumab, tremelimumab, propranolol	Block β receptors and inhibit catecholamine‐driven tumor progression	Ongoing	NCT05451043
Esophageal or gastroesophageal junction adenocarcinoma		Propranolol, pembrolizumab		Ongoing	NCT05651594
Cancer pain	Antibody against NGF	Tanezumab	Targets and binds to NGF to exert analgesic effects	Completed	NCT00545129
Melanoma	β‐Adrenergic antagonist	Propranolol, pembrolizumab	Block β receptors and inhibit catecholamine‐driven tumor progression	Completed	NCT03384836
		Propranolol, naltrexone		Ongoing	NCT05968690
Bladder cancer		Propranolol, pembrolizumab		Ongoing	NCT04848519
Soft tissue sarcoma				Ongoing	NCT05961761

*Data source*: ClinicalTrials.gov, data as of August 16, 2025.

##### 5.1.1.1 Targeting DRs

DA exerts broad immunoregulatory functions through five G protein‐coupled receptors (D1–D5) expressed on various immune cells [360]. Its immunomodulatory effects are highly context dependent. D1‐like receptors (D1 and D5) typically increase intracellular cAMP levels and are often associated with proinflammatory effects—for example, promoting Th17 and Th2 polarization [581, 582]. In contrast, D2‐like receptors (D2, D3, and D4) decrease cAMP levels and generally exhibit immunosuppressive functions, such as D2 receptor activation inducing IL‐10 production [344]. Interestingly, activated T cells and DCs are capable of synthesizing and storing DA themselves, suggesting a potential paracrine regulatory loop within the immune system [360]. Through these mechanisms, DA signaling can either enhance immune responses—for instance, D3 receptor activation promotes TNF‐α and IFN‐γ production by T cells—or suppress them, as D4 receptor signaling pushes T cells toward a quiescent state [345, 583]. Dysregulation of DA signaling has been increasingly implicated in immune‐mediated diseases. In MS, T cells show altered DR expression, such as D5 receptor downregulation impairing the suppressive capacity of Treg cells [584]. Experimental models have shown that D1 receptor blockade significantly inhibits pathogenic Th17 responses [581], suggesting that selective DR antagonists may help modulate T cell‐driven autoimmune inflammation [585]. In PD—a neurodegenerative condition—DA–immune crosstalk also plays a crucial role [586]. Degeneration of midbrain dopaminergic neurons is accompanied by neuroinflammation and peripheral immune infiltration. Notably, CD4⁺ T cell infiltration in the substantia nigra contributes directly to dopaminergic neuron loss [587]. Interestingly, peripheral lymphocytes from PD patients exhibit upregulated DR expression, which can be normalized through treatment with dopaminergic agonists such as L‐DOPA or the D2 agonist bromocriptine—concurrently reducing proinflammatory T cell activity [588]. These observations suggest that restoring DA balance may have immunomodulatory benefits in PD. In the context of cancer, DA has recently emerged as a potential regulator of antitumor immunity. D2 receptor signaling within the TME has been shown to inhibit pathological angiogenesis and promote immune‐mediated tumor suppression [589]. In animal models, exogenous DA or D2 agonists have been reported to normalize tumor vasculature and enhance T cell‐mediated antitumor responses [361]. Epidemiological studies support a protective role: individuals with high dopaminergic activity—such as patients with schizophrenia—exhibit a lower incidence of certain cancers, whereas long‐term use of D2 receptor‐blocking antipsychotics has been associated with increased cancer risk in some populations [590, 591].

These findings have prompted exploration into repurposing clinically approved D2 agonists as novel agents in cancer immunotherapy. In summary, DA signaling possesses dual regulatory roles within the immune system. In diseases such as PD and MS, DA agonists may suppress neuroinflammation and autoimmunity, while in conditions where dopaminergic signaling is pathologically overactive, selective antagonists or inverse modulators may be more effective. Thus, the DA pathway represents a functionally complex neuro‐immune interface—and a therapeutically promising but precision‐demanding target for disease intervention.

##### 5.1.1.2 Targeting Cholinergic Receptors

The CAP represents a well‐established neuro‐immune regulatory mechanism. In this system, ACh released from vagus nerve fibers suppresses inflammation by binding to nAChRs expressed on immune cells [592]. Among these, the α7nAChR is prominently expressed on macrophages, DCs, and other immune populations, where it transduces inhibitory signals that block NF‐κB activation and suppress proinflammatory cytokine production [411, 593, 594]. Activation of α7nAChRs in macrophages significantly inhibits the production of proinflammatory cytokines such as TNF‐α, IL‐1β, and IL‐6—an essential mechanism underlying the anti‐inflammatory effects of VNS [594]. This “cholinergic brake” plays a role in various disease contexts. For instance, in RA, a prototypical autoimmune disorder, α7nAChR expression is markedly upregulated in inflamed synovial tissue [595]. Cholinergic stimulation has been shown to alleviate arthritic symptoms: α7nAChR agonists suppress the production of TNF‐α and other inflammatory mediators in RA patient‐derived synoviocytes and macrophages [596, 597]. Consistently, mice lacking α7nAChR exhibit more severe arthritis and tissue damage in inflammatory joint models, underscoring the receptor's protective role [598]. These findings have prompted the development of therapeutic strategies targeting the cholinergic pathway, including bioelectronic medicine approaches such as VNS and pharmacological agents like α7nAChR agonists (e.g., GTS‐21, encenicline). These interventions are currently under investigation for systemic inflammatory conditions, including RA [599, 600, 601]. Beyond peripheral inflammation, cholinergic–immune crosstalk also plays a critical role in the CNS. Both microglia and astrocytes express α7nAChR and adopt anti‐inflammatory phenotypes upon ACh stimulation [602]. In AD—a condition characterized by chronic neuroinflammation and cholinergic neuron loss—activation of glial nAChRs has shown beneficial effects in preclinical models. Specifically, stimulation of α7nAChR on microglia enhances Aβ phagocytosis and reduces inflammatory signaling, potentially slowing neurodegeneration [603]. This mechanism supports the rationale for using acetylcholinesterase inhibitors (e.g., galantamine, donepezil) and nicotinic receptor agonists in AD clinical studies—not only to improve cognition, but also to mitigate central inflammation by increasing ACh availability [604]. In inflammatory conditions such as sepsis and ulcerative colitis, experimental activation of the CAP—either via VNS or α7 agonists—has been shown to provide protection by limiting cytokine storms and tissue injury [605].

Overall, enhancing cholinergic signaling represents a promising immunomodulatory strategy. By targeting nicotinic receptors on immune cells, ACh agonists can broadly suppress pathological inflammation without inducing systemic immunosuppression—one of the major drawbacks of conventional anti‐inflammatory therapies. Ongoing research is focused on optimizing these approaches, including the development of highly selective α7nAChR agonists and positive allosteric modulators, with potential applications in arthritis, IBD, and neurodegenerative diseases involving central neuroinflammation.

##### 5.1.1.3 Targeting ARs

Sympathetic nerve fibers release NE and Epi, which modulate immune function by binding to ARs on immune cells—namely α₁/α_2_ and β₁/β_2_ subtypes [606]. In general, β_2_‐AR signaling exerts broad anti‐inflammatory effects. For example, activation of β_2_‐ARs on neutrophils inhibits chemotaxis and oxidative burst responses [607], while in monocytes and macrophages, it suppresses the production of proinflammatory cytokines [608]. In DCs, NE binding via β_2_‐ARs alters their ability to activate T cells by promoting a Th1‐to‐Th2 immune shift [609]. Sympathetic signals can also suppress NK cell cytotoxicity [607]. These effects are dose and context dependent. Under acute stress or specific tissue conditions, adrenergic signaling can paradoxically become proinflammatory [610]. A well‐recognized phenomenon is the time‐dependent shift in sympathetic regulation of inflammation during chronic disease. In RA, for example, sympathetic activation during the early disease stage may promote inflammation—possibly through transient α‐AR signaling and NE‐mediated activation of naïve immune cells [611]. As inflammation becomes established, local NE levels deplete, and residual β_2_‐AR signaling begins to exert immunosuppressive effects on innate immune cells, resulting in a net anti‐inflammatory role in later disease stages [611, 612]. This biphasic behavior has been demonstrated in collagen‐induced arthritis models: during early inflammation, chemical sympathectomy or β‐AR blockade worsens disease severity; in contrast, in later stages, removal of NE‐mediated immune suppression exacerbates inflammation [613, 614]. Clinically, joint tissues of patients with chronic RA often show a loss of sympathetic nerve fibers, which is thought to create a localized “inflammatory permissive zone” by removing NE‐mediated regulation [615]. Therefore, the timing of intervention is critical when considering adrenergic modulators for therapy. In the chronic phase of autoimmune diseases, β_2_‐AR agonists may help drive immune resolution by increasing IL‐10 levels and suppressing inflammation [607, 616]. Conversely, during early inflammation, short‐term α‐AR blockade may alleviate symptoms by reducing vasoconstriction and leukocyte infiltration [617, 618]. Several adrenergic drugs are now being repurposed for immune modulation [619]. Small clinical studies have tested β_2_‐agonists (e.g., salbutamol) to promote anti‐inflammatory Th2 responses in MS, or α_2_‐agonists (e.g., dexmedetomidine) to inhibit innate cytokine release in sepsis—though results have been mixed, reflecting the complexity of catecholaminergic regulation [620, 621]. In psychiatric conditions such as posttraumatic stress disorder and depression, heightened sympathetic activity and elevated catecholamine levels are closely linked to inflammation and immune dysregulation [622]. In such settings, β‐blockers (e.g., propranolol) are sometimes used off‐label to blunt stress‐associated immune activation, although central side effects often limit their broader utility [623]. Adrenergic–immune interactions are also increasingly recognized as a targetable pathway in cancer immunology. Chronic psychological stress elevates plasma NE/Epi levels, which act on β‐ARs expressed by tumor‐infiltrating immune and stromal cells, shifting the TME toward immune suppression—via mechanisms such as upregulation of PD‐L1 and inhibition of cytotoxic T cell function [624, 625]. NE also promotes tumor angiogenesis and the release of protumorigenic cytokines [626]. Animal studies in breast, ovarian, and prostate cancer models demonstrate that blocking sympathetic input—through chemical or surgical sympathectomy—or using nonselective β‐blockers can suppress tumor growth and metastasis by enhancing T and NK cell function and inhibiting angiogenesis [627, 628]. For example, in murine prostate cancer models, both sympathectomy and β‐blockade inhibit tumor progression, while VNS surprisingly promotes metastasis [629]. Epidemiological data are consistent with these findings: hypertensive patients treated with β‐blockers have shown improved cancer‐specific survival in some studies, particularly in melanoma and triple‐negative breast cancer [630, 631]. Ongoing clinical trials are now evaluating whether combining β‐blockers (e.g., propranolol or atenolol) with immunotherapy can mitigate adrenergic stress and enhance antitumor immune responses [632].

AR modulators exhibit dual immunoregulatory effects: in autoimmune diseases, β_2_‐agonists may promote anti‐inflammatory responses, while in oncology, β‐blockers offer a means to reverse sympathetic‐driven immune suppression and enhance host antitumor immunity. This bidirectional capacity highlights the therapeutic promise—and the timing‐dependent complexity—of targeting the adrenergic system in immune‐related diseases.

##### 5.1.1.4 Targeting GABAergic Receptors

GABA, the primary inhibitory signaling molecule in the adult CNS, is increasingly recognized for its strong immunosuppressive and anti‐inflammatory effects [633]. Multiple immune populations—such as T lymphocytes, macrophages, and DCs—feature GABA receptor expression alongside endogenous GABA production and transport mechanisms [368]. Within immune cells, GABA generally exerts anti‐inflammatory effects. It inhibits calcium‐dependent signaling pathways and blocks NF‐κB activation, leading to reduced production of proinflammatory cytokines such as IL‐6 and TNF‐α by monocytes and macrophages [368]. GABA receptor activation also suppresses T cell activation and proliferation [634]. This extensive immunoregulatory function has been involved in a range of immune‐related diseases. Impaired GABAergic signaling or receptor dysfunction is consistently associated with worse outcomes in several preclinical models of autoimmunity [635]. Notably, in EAE—an animal model of MS—GABA‐producing cells increase during disease remission. Administration of GABA or receptor‐specific agonists significantly ameliorates disease severity [376, 636]. A 2023 review of in vivo studies showed that both GABA‐A receptor agonists (such as muscimol) and GABA‐B receptor agonists (such as baclofen) markedly suppressed neuroinflammation in EAE models [635]. Treated animals exhibited reduced CNS infiltration by macrophages and T cells, lowered expression of IFN‐γ and IL‐17, and significant clinical improvement. GABAergic stimulation also helped preserve myelin and axonal integrity, indicating secondary neuroprotective benefits [637]. Conversely, genetic deletion or pharmacological blockade of GABA receptors exacerbated inflammation—for example, mice lacking GABA‐B receptors developed more severe arthritis and type 1 diabetes [368, 638]. These findings have encouraged new efforts to repurpose GABAergic drugs for immunotherapy. For instance, baclofen (a GABA‐B receptor agonist) is already used clinically in MS patients with spasticity and has been reported to reduce inflammatory relapses [639]. Other antiepileptic drugs that increase GABA availability, such as vigabatrin and valproic acid, have shown the ability to suppress Th1 and Th17 responses in vitro, suggesting potential utility in autoimmune disease management [376, 640]. Homotaurine, a GABA‐A receptor agonist capable of crossing the blood–brain barrier, has demonstrated significant therapeutic effects in EAE models—delaying onset of paralysis and reducing neuroinflammation—paving the way for clinical testing in MS [636]. Beyond autoimmunity, GABAergic modulation is closely associated with chronic pain and neuroinflammatory disorders. In neuropathic pain, loss of inhibitory GABA signaling in the spinal dorsal horn contributes to neuronal hyperexcitability and microglial activation [641]. Restoring GABA activity has been shown to disrupt this pathological neuro‐immune feedback loop [642]. Benzodiazepines—positive allosteric modulators of GABA‐A receptors—exhibit antinociceptive and anti‐inflammatory effects in pain models, partly by suppressing microglial release of IL‐1β and TNF‐α [643]. Gabapentin, although acting primarily through calcium channels, also enhances GABA availability and has been shown in early clinical studies to reduce inflammatory chemokine levels in patients with neuropathic pain [644].

In summary, GABAergic signaling represents a promising immunomodulatory strategy capable of establishing an anti‐inflammatory and neuroprotective environment in diseases such as MS, RA, type 1 diabetes, and chronic pain. Current research is actively exploring GABA analogs and GABAergic precursor cell transplantation as novel approaches to modulate neuro‐immune interactions.

##### 5.1.1.5 Targeting 5‐HTRs

5‐HT is a multifunctional neurotransmitter with well‐documented immunomodulatory properties [645]. A broad spectrum of immune cells—such as monocytes, DCs, T and B lymphocytes, NK cells, and mast cells—express one or more subtypes of 5‐HTRs [646]. In inflammatory circumstances, specific activated immune cells (for instance, mast cells and T cells) can synthesize and release 5‐HT, which may exert either proinflammatory or anti‐inflammatory effects based on the engaged receptor subtype [647]. In general, increasing extracellular 5‐HT levels dampens excessive immune activation, linking serotonergic signaling to disorders such as depression, autoimmunity, and allergic inflammation. One of the most compelling clinical demonstrations of this link comes from the use of SSRIs, such as fluoxetine and sertraline, which elevate synaptic 5‐HT by blocking reuptake. These agents not only improve depressive symptoms but also induce sustained immunosuppressive effects on peripheral immune cells [648]. Patients with MDD often exhibit low‐grade systemic inflammation, marked by elevated IL‐6 and TNF‐α. Meta‐analyses indicate that SSRI therapy significantly reduces these cytokines while modestly increasing levels of the anti‐inflammatory cytokine IL‐10 [649]. These immune shifts are positively correlated with symptom improvement, suggesting that SSRIs exert part of their efficacy through modulation of inflammatory pathways [649]. At the cellular level, 5‐HT directly suppresses monocyte and macrophage activation. For example, fluoxetine reduces LPS‐induced TNF‐α production and attenuates macrophage and eosinophil infiltration in asthma models [650, 651]. Among the receptor subtypes, 5‐HT2A receptors expressed on immune cells have been shown to potently suppress inflammation by inhibiting NF‐κB activation and downstream expression of adhesion molecules and cytokines [646]. A representative example is (R)‐DOI, a psychedelic 5‐HT2A receptor agonist, which sustainably inhibits TNF‐α‐driven inflammation and protects mice from endotoxin‐induced systemic inflammation and lung injury. This has prompted exploration of sub‐psychedelic doses of 5‐HT2A agonists or related analogs as novel anti‐TNF agents in RA and IBD [652]. Indeed, dysregulation of the serotonergic system has been observed in several TNF‐driven diseases. For instance, RA patients show altered platelet 5‐HT metabolism and elevated Th17 levels; in animal models, 5‐HT deficiency promotes T cell polarization toward a Th17 phenotype [653]. In RA mouse models, peripheral 5‐HT depletion facilitates the conversion of Treg to Th17 cells, aggravating joint inflammation, whereas intact 5‐HT signaling helps preserve Th17/Treg cell balance [653]. This suggests that enhancing serotonergic signaling—via SSRIs or receptor agonists—may help restore immune homeostasis in RA and other autoimmune diseases. Supporting this notion, studies in cultured T cells from MS patients show that exogenous 5‐HT significantly reduces IFN‐γ and IL‐17 production while promoting Treg cell expansion [580]. Clinically, depression is common in MS and is associated with elevated proinflammatory cytokines and reduced central 5‐HT levels [654]. SSRI treatment not only improves mood but is also linked to reduced relapse rates and decreased inflammatory burden. Beyond autoimmunity, 5‐HT also modulates chronic pain and intestinal inflammation. In neuropathic pain, serotonergic and noradrenergic antidepressants—such as the SNRI duloxetine—exert significant analgesic effects, partly through suppression of spinal neuroinflammation [655]. In cancer, 5‐HT released from platelets can influence immune responses and angiogenesis. Certain tumors manipulate local 5‐HT availability to induce immunosuppressive macrophage phenotypes [656]. While this remains an emerging area, interest is growing in whether modulating 5‐HT signaling can reprogram the TME [657]. A notable mechanism involves increased activity of IDO, which depletes tryptophan, thereby reducing 5‐HT synthesis. This has been proposed to contribute to both cancer‐associated depression and T cell dysfunction [658]. As such, IDO inhibition or targeted activation of specific 5‐HTRs on immune cells may hold promise for enhancing antitumor immunity, although these strategies remain under investigation [659]. In sum, 5‐HTR modulators—from broad‐acting SSRIs to subtype‐specific agonists—exhibit strong potential as immunomodulatory agents. By fostering an anti‐inflammatory milieu (e.g., inhibiting Th1/Th17 polarization, mast cell activation, and monocyte cytokine production), they may benefit patients with depression, autoimmune neuroinflammation, asthma, and arthritis. The key to precision modulation lies in receptor specificity. For example, 5‐HT1A receptor agonists can suppress macrophage activity in CNS infections and reduce HIV replication in macrophages [660], while 5‐HT3 antagonists are used to treat gastrointestinal inflammation such as IBS, partly by reducing neuronal excitability and immune activation [661]. As our understanding of the serotonergic neuro‐immune axis expands, future therapies—including anti‐inflammatory molecules derived from psychedelics—may become powerful tools in neuro‐immune modulation.

Neurotransmitter receptor modulators offer a unique pharmacological toolbox for regulating immune responses along the neuro‐immune axis. Unlike traditional broad‐spectrum immunosuppressants, these agents leverage the body's endogenous reflex circuits to achieve targeted immunomodulation. Dopaminergic agonists/antagonists, cholinergic stimulants, adrenergic blockers, GABAergic agonists, and serotonergic compounds each demonstrate disease‐context‐specific utility. As reviewed above, vagal activation enhances ACh release and suppresses joint inflammation in RA; inhibition of sympathetic stress signaling improves antitumor immunity; and upregulating inhibitory neurotransmitters such as GABA and 5‐HT often biases the immune system toward resolution and repair. These interventions influence not just immune cells, but also neurons and glial networks, reshaping intercellular communication across systems. Future progress in this field will rely on mechanistic dissection of disease‐specific neuro‐immune receptor pathways, enabling rational selection of receptor targets and their corresponding ligands to recalibrate immune function. Encouragingly, multiple neurotransmitter‐based immunotherapies—such as vagus nerve stimulators, β‐blockers combined with immune checkpoint inhibitors, and novel anti‐inflammatory psychedelic derivatives—are already advancing into clinical trials. Neuro‐immune crosstalk is not merely a theoretical concept; it is becoming a translational frontier across neurology, immunology, psychiatry, pain medicine, and oncology. By harnessing endogenous neuromodulatory signals, we may unlock new therapeutic paradigms that restore immune homeostasis in complex disease states.

#### 5.1.2 Cytokine/Anticytokine Therapies

Cytokines are key messengers in neuro‐immune communication, regulating the interactions between immune cells and neurons in health and disease [327]. Proinflammatory cytokines (e.g., TNF‐α, IL‐1β, IL‐6, IFN‐γ) are typically elevated during neuroinflammation, driving aberrant immune activation and neurological dysfunction [662]; while anti‐inflammatory cytokines (e.g., IL‐10, TGF‐β) balance inflammation and prevent excessive tissue damage [662]. The balance between proinflammatory and anti‐inflammatory signaling is vital for preserving homeostasis [663]. Proinflammatory cytokines not only recruit and activate immune cells but also profoundly influence neural signaling [664]. For instance, elevated TNF‐α and IL‐1β in the CNS enhance glutamatergic transmission and oxidative stress, leading to synaptic dysfunction and neuronal death [665, 666]; IL‐6 can cross the BBB, altering neurotransmitter metabolism and neuronal plasticity, thereby linking peripheral inflammation to central depression [667]; high concentrations of IFN‐γ activate microglia and astrocytes, suppressing neurogenesis and inducing a neurotoxic environment [662, 664]. Conversely, anti‐inflammatory factors like IL‐10 and TGF‐β typically suppress microglial activation and promote tissue repair, with IL‐10 release in the CNS protecting neurons by inhibiting proinflammatory cytokine production from microglia [668]. Notably, some anti‐inflammatory factors (e.g., TGF‐β) exhibit duality: protective in autoimmunity but exploited by tumors to suppress immunity [669]. Overall, cytokine signaling regulates both immune cell behavior (e.g., T cell polarization, microglial phagocytosis) and neural cell function (e.g., synaptic transmission, survival pathways), highlighting its central role in neuro‐immune disorders and its importance as a target for pharmacological intervention.

##### 5.1.2.1 Targeting Proinflammatory Cytokines

In neurodegenerative diseases such as AD and PD, excessive proinflammatory cytokine activity is a key driver of disease progression [670, 671]. Persistently elevated levels of TNF‐α, IL‐1β, and IL‐6 in the brain and peripheral circulation of AD patients are believed to exacerbate amyloid plaque deposition and tau pathology through chronic inflammation [672]. Consequently, cytokine‐targeted therapies are considered a strategy to delay neurodegeneration (Table 4). Some small studies suggest that paraspinal administration of the TNF blocker etanercept may improve cognitive and behavioral performance in AD patients [673]. However, a placebo‐controlled trial found that systemic administration of etanercept provided no significant cognitive improvement in patients with mild‐to‐moderate AD [674]. This indicates that blocking peripheral TNF alone may be insufficient, potentially requiring combination with BBB‐penetrating agents or direct targeting of specific TNF forms (e.g., soluble TNF) [675]; a Phase II clinical trial (NCT05318976) indicates that XPro1595, a selective soluble TNF inhibitor, demonstrates potential in early AD trials. In PD, microglia‐derived TNF‐α and IL‐1β contribute to the loss of dopaminergic neurons [676]. Preclinical studies confirm that inhibiting TNF‐α mitigates neurodegeneration in PD animal models—for example, thalidomide (which inhibits TNF synthesis) protects mice from neurotoxin‐induced neuronal damage [677]. Although anticytokine therapies for human PD also show promise in slowing disease progression, clinical evidence remains limited.

**TABLE 4:** Clinical trials of immunotherapeutic drugs for neurodegenerative and autoimmune diseases.

Diseases	Target	Drug/compound	Mechanism	Status	ClinicalTrials.gov identifier
Alzheimer's disease	Amyloid β	Solanezumab	Directly bind to eliminate Aβ	Terminated	NCT01127633
				Terminated	NCT01900665
				Completed	NCT01148498
				Terminated	NCT02760602
		Donanemab		Active, not recruiting	NCT04437511
		Crenezumab		Terminated	NCT02670083
				Terminated	NCT03114657
		Sabirnetug		Active, not recruiting	NCT06335173
		Lecanemab		Recruiting	NCT06810960
				Active, not recruiting	NCT04468659
				Completed	NCT01767311
				Recruiting	NCT06322667
				Recruiting	NCT06871839
				Recruiting	NCT06741553
				Active, not recruiting	NCT07034222
		Bapineuzumab		Completed	NCT00112073
				Completed	NCT00575055
		Gantenerumab		Terminated	NCT05256134
		Aducanumab (BIIB037)		Terminated	NCT01677572
		Amilomotide (CAD106)	Activate the body's own immune system to produce specific antibodies against Aβ	Completed	NCT00411580
				Completed	NCT00733863
				Completed	NCT00795418
				Completed	NCT01023685
				Completed	NCT00956410
		ACI‐24.060		Recruiting	NCT05462106
		AV‐1959R		Active, not recruiting	NCT06831812
		AV‐1959D		Active, not recruiting	NCT05642429
		AFFITOPE AD01		Completed	NCT00495417
		AFFITOPE AD02		Completed	NCT01093664
		V950		Completed	NCT00464334
		AAB‐003 (PF‐05236812)		Completed	NCT01369225
		ACC‐001		Completed	NCT01227564
				Completed	NCT00752232
				Completed	NCT00498602
				Completed	NCT00955409
				Completed	NCT00479557
		ABvac40		Completed	NCT03461276
		SHR‐1707		Recruiting	NCT06199037
				Completed	NCT06114745
				Active, not recruiting	NCT05681819
		ALZ‐101		Completed	NCT05328115
		UB‐311		Completed	NCT02551809
		Lu AF20513		Terminated	NCT03668405
	Microglia	Foralumab	Block CD3	Not yet recruiting	NCT06489548
		ALZT‐OP1a	Promote the elimination of Aβ by microglia	Completed	NCT04570644
		ALZT‐OP1		Completed	NCT02547818
		Sargramostim	Activate CSF2R	Withdrawn	NCT02667496
		JNJ‐40346527	Inhibit CSF1R	Terminated	NCT04121208
		AL002	Activate the TREM2 pathway	Completed	NCT03635047
		AL002		Terminated	NCT05744401
		AL002		Completed	NCT04592874
		AL003	Block CD33	Completed	NCT03822208
	Tau protein	TRx0237	Reduce the number of neurofibrillary tangles and remove overphosphorylated tau protein	Terminated	NCT01626391
				Completed	NCT01689246
		JNJ‐64042056 (ACI‐35)		Recruiting	NCT06544616
				Completed	NCT04445831
		PTI‐125 (sumifilam)		Completed	NCT04079803
		Tilavonemab (ABBV‐8E12)		Terminated	NCT03712787
				Completed	NCT02880956
		AADvac1		Completed	NCT02031198
		JNJ‐54861911 (Atabecestat)		Completed	NCT03587376
	Brain–gut axis	GV‐971	Regulate the intestinal flora and reduce the production of harmful metabolites	Recruiting	NCT05908695
		Probiotics		Completed	NCT05145881
	TNF	Etanercept	Inhibit TNF	Completed	NCT01716637
				Completed	NCT00203359
				Completed	NCT00203320
				Completed	NCT01068353
		Infliximab		Completed	NCT04571697
		Golimumab			
		Certolizumab			
		Adalimumab			
		XPro1595		Completed	NCT03943264
				Active, not recruiting	NCT05522387
				Withdrawn	NCT05321498
				Active, not recruiting	NCT05318976
	IL‐1β	Canakinumab	Inhibit IL‐1β	Terminated	NCT04795466
	IL‐2R	IL‐2	Restore functional Treg cells	Recruiting	NCT06096090
				Completed	NCT05821153
				Recruiting	NCT05468073
	INF‐α	IFN‐α2A	Inhibit INF‐α	Unknown status	NCT00031018
	Anti‐inflammatory	Ibuprofen	Inhibit COX	Completed	NCT04570645
		Naproxen		Completed	NCT00004845
		Celecoxib		Completed	NCT00065169
		Rofecoxib		Completed	NCT00004845
		Indomethacin		Completed	NCT00432081
		Doxycycline	Resist Aβ deposition	Completed	NCT00439166
		Rifampicin		Completed	NCT00439166
				Completed	NCT01002079
		Minocycline	Inhibit inflammasome	Completed	NCT01463384
		Curcumin	Inhibit the NF‐κB and MAPK pathways	Completed	NCT00099710
				Completed	NCT00164749
		NE3107	Inhibit the NF‐κB and ERK pathways	Completed	NCT04669028
		VX‐745	Inhibit the p38 MAPK pathways	Completed	NCT03435861
		MW150		Unknown status	NCT05194163
		Pepinemab	Block SEMA4D	Completed	NCT04381468
		Prednisone	Glucocorticoid	Completed	NCT00000178
	Immunomodulators	Thalidomide	Immunosuppression	Unknown status	NCT01094340
		Cyclophosphamate		Completed	NCT00013650
		Rapamycin		Recruiting	NCT04629495
		Tacrolimus		Withdrawn	NCT04263519
	α‐Synuclein	UB‐312	Stimulate an immune response against α‐synuclein	Completed	NCT05634876
		ACI‐7104.056		Recruiting	NCT06015841
	Microglia	Sargramostim	Activate CSF2R	Completed	NCT01882010
				Completed	NCT05677633
		Semagludtide	Activate GLP1R	Unknown status	NCT03659682
		Verdiperstat	Inhibit MPO	Completed	NCT04616456
		BHV‐8000	Inhibit TYK2/JAK1	Recruiting	NCT06976268
	Brain–gut axis	Probiotics	Regulate the intestinal flora and reduce the production of harmful metabolites	Unknown status	NCT05173701
		Rifaximin		Unknown status	NCT03958708
	NLRP3	VTX3232	Inhibit NLRP3	Completed	NCT06556173
		IZD174		Withdrawn	NCT04338997
		VENT‐02		Recruiting	NCT06822517
		HL‐400		Recruiting	NCT06997484
	CDNF	HER‐096	Regulate neurotrophic–immune imbalance	Active, not recruiting	NCT06659562
				Completed	NCT05915247
	Anti‐inflammatory	Pentoxifylline (PTX)	Inhibit the TLR4/NF‐κB pathway	Recruiting	NCT05962957
		Celecoxib	Inhibit COX		
		Cilostazol	Inhibit the NF‐κB pathways	Not yet recruiting	NCT06612593
		Montelukast		Recruiting	NCT06113640
		Niacin	Activate GPR109A	Completed	NCT03462680
					
	S1R	Pridopidine	Strengthen neuroprotective pathways and inhibit neuroinflammation and oxidative stress	Terminated	NCT02494778
				Completed	NCT02006472
				Completed	NCT00665223
				Completed	NCT01306929
				Completed	NCT04556656
				Completed	NCT00724048
	C1q	ANX005	Inhibit the excessive activation of the complement system and alleviate neuroinflammation	Completed	NCT04514367
	BDNF	NestaCell	Regulate neurotrophic–immune imbalance	Not yet recruiting	NCT06097780
	Anti‐inflammatory	Minocycline	Inhibit HIF‐1α	Completed	NCT00029874
				Completed	NCT00277355
		Resveratrol	Inhibit the NF‐κB pathways	Completed	NCT02336633
		Pepinemab (VX15/2503)	Block SEMA4D	Completed	NCT02481674
Amyotrophic lateral sclerosis	IL‐2R	IL‐2	Restore functional Treg cells	Completed	NCT03039673
				Unknown status	NCT04055623
				Unknown status	NCT04952155
		Abatacept and IL‐2		Active, not recruiting	NCT06307301
		Basiliximab			
	IL‐6R	Tocilizumab	Inhibit IL‐6	Completed	NCT02469896
	IL‐10R	XT‐150	Inhibit IL‐10	Not yet recruiting	NCT06704347
	IL‐1R	Anakinra	Inhibit IL‐1	Completed	NCT01277315
	TNF	Thalidomide	Inhibit TNF	Terminated	NCT00231140
	C1q	ANX005	Inhibit the excessive activation of the complement system and alleviate neuroinflammation	Completed	NCT04569435
	Anti‐inflammatory	Tofacitinib	Inhibit JAK	Not yet recruiting	NCT06689982
		ALZT‐OP1a (cromolyn)	Mast cell stabilizer	Terminated	NCT04428775
		Withania somnifera	Inhibit the NF‐κB pathways	Unknown status	NCT05031351
		MN‐166 (ibudilast)	Inhibit MIF	Completed	NCT02714036
		Rapamycin	Inhibit mTOR	Completed	NCT03359538
		Methylprednisolone	Glucocorticoid	Completed	NCT01884571
		Prednisone		Completed	NCT01884571
	Immunomodulators	Tacrolimus	Immunosuppression	Completed	NCT01884571
		Mycophenolate mofetil		Completed	NCT01884571
Multiple sclerosis	T cell/B cell	Alemtuzumab	Target CD52	Unknown status	NCT01624714
				Terminated	NCT03647722
		Ocrelizumab	Target CD20	Recruiting	NCT05999604
				Completed	NCT03138525
		Rituximab		Completed	NCT03193866
		Natalizumab	Target CD49d	Completed	NCT01077466
				Completed	NCT00559702
		Daclizumab	Target CD25	Completed	NCT00001934
				Completed	NCT00071838
	INF	IFN‐β‐b	Regulate cytokines	Completed	NCT00963833
		IFN‐β‐a		Completed	NCT01939002
				Recruiting	NCT00210301
	IL‐2Ra	Zenapax	Restore functional Treg cells	Completed	NCT00001934
	/	Glatiramer acetate	Regulate the immune system	Completed	NCT00819195
				Completed	NCT01874145
		Laquinimod		Completed	NCT01707992
				Completed	NCT00349193
		Cladribine tablet		Completed	NCT04997148
				Completed	NCT03961204
		Mitoxantrone hydrochloride		Terminated	NCT00146159
				Completed	NCT01214317
		SAR442168		Completed	NCT03996291
Rheumatoid arthritis	TNF	Adalimumab	Inhibit TNF	Recruiting	NCT05626348
		Adalimumab		Approved for marketing	NCT00650026
		Cimzia		Completed	NCT01147341
		Etanercept		Completed	NCT03915964
		Infliximab		Completed	NCT00269867
		Infliximab		Completed	NCT02222493
		PF‐06438179		Completed	NCT02222493
		TNF‐kinoid		Completed	NCT01911234
				Completed	NCT01040715
		ISIS 104838		Completed	NCT00048321
	IL‐6	ALD518	Inhibit IL‐6	Completed	NCT00867516
		Vobarilizumab (ALX‐0061)		Completed	NCT02518620
		Sarilumab		Completed	NCT02404558
				Completed	NCT01606761
		Tocilizumab		Unknown status	NCT01835613
		Gerilimzumab		Unknown status	NCT04179513
		Levilimab (BCD‐089)		Completed	NCT04227366
	IFN	Anifrolumab	Inhibit IFN	Unknown status	NCT03435601
	B cell	KYV101	Target CD19, induce B‐cell exhaustion	Not yet recruiting	NCT06475495
		Rituximab	Target CD20, induce B‐cell exhaustion	Not yet recruiting	NCT06475495
				Terminated	NCT03161457
		MabThera (Rituximab)		Completed	NCT02468791
		MabionCD20		Completed	NCT02468791
		JHL1101		Completed	
	LTβR	Baminercept (BG 9924)	Block the LTβR pathway, inhibit CXCL13	Terminated	NCT00458861
	TLRs	Hydroxychloroquine	Inhibit TLR7/9	Recruiting	NCT05626348
		Rob 803	Down‐regulate TLR2 and TLR4	Completed	NCT00525213
	Other anti‐inflammatory drugs	LY3337641	Inhibit BTK	Terminated	NCT02628028
		ABBV‐105		Completed	NCT03682705
		Upadacitinib	Inhibit JAK	Completed	NCT02049138
		Upadacitinib		Active, not recruiting	NCT05814627
		Upadacitinib		Completed	NCT02706951
		Upadacitinib		Completed	NCT02706847
		Baricitinib		Recruiting	NCT04870203
		Tofacitinib (CP‐690550)		Completed	NCT00976599
		Iguratimod	Inhibit COX‐2	Recruiting	NCT05626348
	Immunomodulators	Methotrexate	Immunosuppression	Recruiting	NCT05626348
		Leflunomide		Recruiting	NCT05626348
Systemic lupus erythematosus	IL‐6	MRA 003	Inhibit IL‐6	Completed	NCT00046774
		Sirukumab (CNTO‐136)		Completed	NCT01702740
		Vobarilizumab (ALX‐0061)		Completed	NCT02437890
	IL‐2R	IL‐2	Restore functional Treg cells	Completed	NCT04077684
				Recruiting	NCT05339217
				Completed	NCT02084238
				Completed	NCT04397107
				Completed	NCT03312335
	IFN	Anifrolumab	Inhibit IFN	Completed	NCT05001698
				Not yet recruiting	NCT06795893
				Recruiting	NCT05835310
				Completed	NCT01753193
				Completed	NCT02962960
				Completed	NCT01438489
		Sifalimumab		Completed	NCT00979654
				Completed	NCT01283139
				Completed	NCT00657189
				Completed	NCT00299819
	B cell	Rituximab	Target CD20, induce B‐cell exhaustion	Completed	NCT00137969
				Completed	NCT00036491
		Telitacicept	Inhibit BLyS and APRIL	Recruiting	NCT06137053
		Belimumab	Inhibit B cell stimulating factors	Completed	NCT05624437
		Eque‐cel	Target BCMA	Recruiting	NCT06902844
		Epratuzumab	Target CD22, induce B‐cell exhaustion	Completed	NCT00011908
				Completed	NCT01449071
				Completed	NCT01262365
				Completed	NCT01261793
		CD19 CAR‐T cells	Target CD19, induce B‐cell exhaustion	Recruiting	NCT06585514
	B cell and T cell	Rozibafusp alfa (AMG‐570)	Target ICOSL and BAFF	Completed	NCT04058028
		A‐319	Target CD19 and CD3	Recruiting	NCT06400537
	TLR	DS‐7011a	Target TLR7	Completed	NCT05203692
	FcRn	Nipocalimab	Block FcRn	Completed	NCT04882878
	Other anti‐inflammatory drugs	Tofacitinib	Inhibit JAK/STAT pathway	Completed	NCT05048238
				Completed	NCT03288324
				Terminated	NCT03159936
		Upadacitinib		Completed	NCT04451772
				Completed	NCT03978520
Psoriasis	IL‐23	Guselkumab	Inhibit IL‐23	Completed	NCT02325219
				Completed	NCT04914429
				Completed	NCT04439526
				Completed	NCT02343744
				Active, not recruiting	NCT04882098
		Icotrokinra		Recruiting	NCT06807424
				Recruiting	NCT06878404
		Tildrakizumab		Active, not recruiting	NCT04340076
				Recruiting	NCT05390515
				Terminated	NCT04339595
				Completed	NCT04203693
		Risankizumab		Active, not recruiting	NCT04340076
				Completed	NCT05283135
				Completed	NCT03875508
	IL‐17	Bimekizumab	Inhibit IL‐17	Recruiting	NCT06336343
				Completed	NCT03896581
				Completed	NCT03347110
				Completed	NCT03230292
		Secukinumab		Active, not recruiting	NCT04340076
				Completed	NCT05320159
				Completed	NCT02592018
		Ixekizumab		Active, not recruiting	NCT04340076
				Completed	NCT03073213
				Not yet recruiting	NCT06374979
		Brodalumab		Active, not recruiting	NCT04340076
				Completed	NCT03403036
				Completed	NCT04149587
	Other anti‐inflammatory drugs	Apremilast	Inhibit PDE4	Completed	NCT00606450
				Active, not recruiting	NCT04175613
				Completed	NCT03123471
		Tofacitinib	Inhibit JAK	Completed	NCT01831466
				Completed	NCT03486457

*Data source*: ClinicalTrials.gov, data as of August 16, 2025.

Systemic autoimmune diseases provide the clearest examples of successful cytokine‐targeted therapies, with some also demonstrating significant neuro‐immune modulatory effects. In RA, its pathogenesis is driven by proinflammatory cytokines, which not only destroy joints but also induce pain sensitization [678]. Anti‐TNF biologics (e.g., infliximab, etanercept, adalimumab) have revolutionized RA treatment, significantly suppressing inflammation and preventing disability [679]. By neutralizing TNF‐α, they not only inhibit intra‐articular immune responses but also alleviate RA‐associated neuropathic pain and fatigue, illustrating the neurological benefits of immune modulation [679]. Similarly, blocking the IL‐6 pathway (e.g., with tocilizumab) is highly effective in RA, helping many patients achieve clinical remission [680]. Although IL‐1 also plays a role in RA, the IL‐1 receptor antagonist anakinra is generally less effective than TNF or IL‐6 inhibitors [681]. Notably, RA patients frequently report improved cognition and stabilized mood following cytokine‐inhibiting therapy, likely because suppressing elevated systemic inflammatory factors like IL‐6 and TNF blocks their induction of depression and fatigue via neural pathways, thereby conferring additional benefits to the CNS [681]. However, cytokine functions are complex, necessitating therapeutic caution: despite their anti‐inflammatory effects, TNF‐α inhibitors worsened disease in MS trials by interfering with myelin repair [682]. In SLE, a classic neuro‐immune disease, type I IFNs drive immune dysregulation and neuropsychiatric symptoms [683]. SLE patients exhibit a characteristic “IFN signature”; excess IFN‐α can disrupt the BBB, alter neurotransmitters, and cause cognitive impairment [683]. The new drug anifrolumab inhibits this pathway by targeting the type I IFN receptor (IFNAR1), with studies showing it significantly improves moderate‐to‐severe SLE symptoms, reduces flares, and may protect organs—including the CNS—from IFN‐mediated damage [684]. By neutralizing IFN, anifrolumab effectively suppresses downstream proinflammatory factors and autoantibody production, demonstrating that anticytokine strategies can rebalance immune responses in autoimmune diseases [685].

Chronic pain syndromes, including FM and neuropathic pain, have been shown to be closely linked to neuro‐immune dysregulation. For instance, FM patients frequently exhibit elevated serum levels of TNF‐α, IL‐6, and IL‐8, indicating the presence of mild chronic inflammation [258]. These cytokines activate peripheral nerves and spinal microglia, lowering the pain threshold and inducing central sensitization (i.e., amplification of pain signals by the CNS) [686]. Indeed, excessive cytokine release can “overactivate” both PNS and CNS, leading to persistent pain even in the absence of obvious tissue damage [687]. Consequently, targeting cytokines has emerged as a novel strategy for treating such primary pain conditions. Animal studies demonstrate that blocking IL‐1β or TNF‐α alleviates neuropathic pain, primarily by disrupting the neuro‐glial inflammatory reflex circuit that promotes hyperalgesia [688]. However, a small pilot study on chronic fatigue syndrome (which overlaps with FM) showed that the IL‐1 receptor antagonist anakinra, while safe, failed to significantly improve symptoms, highlighting the complexity of the disease mechanisms [689]. Nevertheless, modulating immune mediators to alleviate pain is still considered to have potential.

Growing evidence links some neuropsychiatric disorders to underlying inflammation. For instance, MDD patients often show elevated peripheral IL‐6, TNF‐α, and C‐reactive protein (CRP) levels [690, 691]. These cytokines can enter the CNS, disrupting neurotransmitter metabolism (e.g., 5‐HT and DA pathways) and triggering depressive symptoms [692]. Consequently, researchers are exploring cytokine‐targeted therapies as adjunct antidepressant treatments. Notably, TNF‐α antagonists like infliximab have been tested in depression: a randomized controlled trial found that while infliximab overall did not outperform placebo, it significantly improved mood symptoms in the high baseline inflammation subgroup (CRP > 5 mg/L) [693]. This suggests cytokine blockers may help inflammation‐driven depression, potentially by correcting monoamine neurotransmitter imbalances and downregulating the overactivated HPA axis. Additionally, autoimmune disease patients often report reduced depression as an “incidental benefit” after TNF inhibitor use [694]. Although IL‐6 also plays a key role in depression pathology, IL‐6‐targeted approaches (e.g., tocilizumab for residual depression) show inconsistent results and may even worsen mood in some patients, underscoring the need for precision therapy based on immune profiles [695]. Schizophrenia—traditionally viewed as a neurological disorder—also involves immune dysfunction, with patients frequently exhibiting elevated IL‐6, IL‐1β, and TNF‐α levels that correlate with disease severity. Neuroinflammation is considered a key mechanism underlying cognitive deficits and negative symptoms [696]. Initial trials (e.g., tocilizumab targeting IL‐6) successfully lowered peripheral inflammation markers but failed to improve psychotic symptoms or cognition [697]. This “pathway activation without clinical efficacy” may stem from poor central drug penetration or inflammation being secondary in chronic schizophrenia. However, open‐label studies using adjunctive anti‐inflammatories (e.g., NSAIDs, minocycline, aspirin) to indirectly suppress cytokines report mild symptom improvements, supporting the role of neuro‐immune mechanisms [698]. Importantly, inflammation itself can directly drive psychiatric disorders—as seen when IFN‐α therapy for hepatitis C frequently induces major depression, a classic example of cytokine‐induced neurotoxicity. This further confirms cytokines can bidirectionally modulate mood and behavior [699]. Current research aims to stratify patients using inflammatory biomarkers to identify subgroups most likely to benefit from anticytokine therapy [699]. In summary, while not yet standard care, cytokine/anticytokine therapies show promise as precision adjuncts in psychiatry—suppressing pathological immune signals and restoring neurotransmitter function to improve mental health in immune‐hyperactivated patients.

Cancer provides a unique context for cytokine‐targeted therapies, wherein interventions can simultaneously enhance antitumor immune responses and alleviate tumor‐associated neurological dysfunction [700]. Within the TME, tumor cells, immune infiltrates, and components of the nervous system engage in complex cytokine‐mediated communication that shapes both tumor progression and neuro‐related symptoms. Research shows IL‐6 is crucial in neuro‐immune crosstalk in pancreatic cancer—tumor and surrounding cells secrete IL‐6, which promotes tumor survival, drug resistance, and systemic symptoms like muscle wasting (cachexia), depression, and fatigue. Therefore, IL‐6 is a potential target for pancreatic cancer. Preclinical studies demonstrate that blocking IL‐6 combined with immune checkpoint inhibitors significantly suppresses pancreatic tumor growth [701]. Currently, a Phase II clinical trial is evaluating the IL‐6 antibody (tocilizumab) combined with chemotherapy for pancreatic cancer, aiming to improve treatment response and alleviate cachexia [702]. Preliminary data suggest anti‐IL‐6 therapy reduces weight loss and inflammatory symptoms, indicating benefits for tumor‐related neurological/metabolic complications. Additionally, inflammatory factors like TNF‐α and IL‐1β contribute to cancer pain by activating peripheral nerves. This is particularly evident in pancreatic cancer, which often involves severe neuropathic pain (tumor‐infiltrating immune cells release factors that stimulate nerve fibers) [703]. Consequently, TNF‐α blockers targeting cancer cachexia and pain are being tested to improve patient quality of life [704]. Importantly, some immune factors (like IFNs) are themselves used in cancer treatment (e.g., IFN‐α for melanoma) but can cause neurotoxicities such as depression and cognitive impairment. This highlights the critical need to balance efficacy with neuro‐immune tolerance in anticancer therapy [705].

##### 5.1.2.2 Targeting Antiinflammatory Cytokines

Therapeutic inhibition of anti‐inflammatory cytokines represents a critical strategy for managing neuro‐immune disorders. In SLE, IL‐10 and TGF‐β are generally protective, enhancing Treg cell activity. However, their endogenous levels are often insufficient in severe cases, leading to ineffective inflammation control [706]. For chronic pain syndromes, boosting anti‐inflammatory cytokines is a promising approach. Animal studies show that increasing IL‐10 expression in the CNS alleviates neuropathic pain by suppressing microglial proinflammatory activity [707]. Research is exploring gene delivery to introduce IL‐10 into spinal tissue for refractory pain [708]. Another strategy uses drugs that inhibit glial activation, like minocycline or JAK–STAT pathway inhibitors, to indirectly reduce cytokine release in the CNS. These show potential in early trials for alleviating pain and fatigue [709]. Although no cytokine‐targeting drugs are widely used for FM, the consistent link between elevated cytokine levels and symptom severity highlights the neuro‐immune axis as an attractive therapeutic target. Future treatments may rebalance cytokines, such as neutralizing IL‐6 (strongly linked to muscle pain and fatigue) or enhancing neuroprotective cytokines. This could suppress excessive immune cell activation while repairing abnormal nerve signaling in chronic pain. Additionally, gliomas often secrete high levels of TGF‐β and IL‐10, creating a potent immunosuppressive microenvironment. This not only aids tumor immune evasion and impairs neural function, but TGF‐β also directly promotes tumor invasion and peritumoral edema [710]. Consequently, targeting these cytokines is a research focus. TGF‐β inhibitors show promise in GBM: blocking its signaling restores antitumor immune surveillance and delays progression [711]. Animal models confirm TGF‐β antagonists, including TGF‐β2 antisense oligonucleotides and small‐molecule receptor kinase inhibitors, inhibit tumor growth and extend survival [712]. In early clinical trials, the TGF‐β2 antisense agent trabedersen showed benefit in some patients with recurrent glioma [713]. By alleviating TGF‐β‐mediated immunosuppression, these therapies may activate cytotoxic T cells and microglia to recognize and clear tumor cells. They might also reduce tumor‐induced seizures and intracranial pressure, as TGF‐β promotes peritumoral inflammation and fluid accumulation [714]. Another innovative strategy designs TGF‐β‐resistant CAR‐T cells or oncolytic viruses to circumvent its suppression of immune clearance [715].

Cytokine and anticytokine therapies have emerged as a central strategy for targeting the neuro‐immune axis, with broad applicability across diverse disease contexts. By suppressing key proinflammatory cytokines or enhancing anti‐inflammatory signaling, these interventions can modulate both immune activity and nervous system function. Whether in AD (via TNF‐α blockade reducing synaptotoxicity), autoimmune diseases (via IL‐6 or IFN‐α antagonism mitigating neuroinflammation), or cancer‐associated syndromes (via IL‐1/IL‐6 neutralization alleviating pain and cachexia), cytokine modulation demonstrates a dual therapeutic mechanism: restoring immune balance and alleviating associated neurological complications.

Future advances in our understanding of neuro‐immune cytokine networks will enable more precise, targeted interventions—such as selectively blocking the neurotoxic pathways of TNF‐α while preserving its physiological immune functions. Although cytokine therapy is not yet a universal solution, it has already opened multiple new therapeutic avenues. When integrated with conventional neuroprotective or immunomodulatory strategies, personalized cytokine‐targeted interventions may offer improved outcomes in neurodegenerative diseases, autoimmune conditions, psychiatric disorders, chronic pain syndromes, and malignancies. Every new discovery reinforces the inseparability of the immune and nervous systems—and highlights cytokines as modifiable nodal points with the potential to unravel and reverse complex disease processes.

### 5.2 Bioelectronic Medicine

Bioelectronic medicine represents a cutting‐edge therapeutic paradigm that leverages electrical or optical stimulation to modulate neural circuits and, by extension, immune responses. Unlike traditional pharmacological interventions, which rely on systemic drug delivery, bioelectronic approaches offer precise, real‐time control over neural activity, making them particularly attractive for conditions involving dysregulated neuro‐immune communication. Notably, techniques such as VNS and optogenetic neuromodulation have shown promising results in both preclinical models and early‐phase clinical trials across a range of inflammatory, autoimmune, neurodegenerative, and neoplastic diseases. In the following sections, we discuss these modalities in detail, with emphasis on their mechanisms of action, disease‐specific applications, and emerging translational opportunities.

#### 5.2.1 Vagus Nerve Stimulation

VNS is a neuromodulatory strategy that leverages the vagus nerve–immune circuit to regulate inflammation. Applying electrical impulses to the vagus nerve triggers the inflammatory reflex—a descending neural circuit mediated by cholinergic signaling—that curbs immune system activity [716]. In this neural circuit, outgoing vagus nerve signals initiate ACh secretion. This neurotransmitter subsequently binds α7nAChRs on immune cells (e.g., macrophages), resulting in blockade of NF‐κB nuclear transport and inflammasome assembly. This ultimately restrains production of proinflammatory mediators like TNF, IL‐1β, and IL‐6 [717].

At the same time, afferent vagal fibers convey peripheral immune signals to the NTS in the brainstem, where central autonomic circuits are activated to further modulate immune responses via descending signals [718]. For example, activation of afferent vagal input can stimulate the HPA axis to release cortisol, exerting systemic anti‐inflammatory effects. Meanwhile, efferent vagal signaling—via the vagovagal reflex involving the DMN of the vagus and sympathetic innervation of the spleen—can directly inhibit inflammation in the spleen and other peripheral organs [719].

VNS can also activate the brainstem's locus coeruleus (LC), leading to widespread release of NE in the brain, which enhances synaptic plasticity and dampens neuroinflammatory signaling [720]. Through these multiple mechanisms, VNS enables rapid suppression of inflammation. Indeed, animal studies have shown that electrical stimulation of the vagus nerve significantly reduces cytokine release and disease severity in models of endotoxemia, sepsis, arthritis, and colitis [721]. This neuromodulatory approach demonstrates the therapeutic potential of VNS across a range of inflammatory diseases.

RA is a landmark case in the clinical application of VNS for inflammatory diseases. The first human trial conducted in 2016 demonstrated that implantable VNS devices significantly improved disease activity in RA [61]. In this open‐label study, 17 patients with refractory RA who had failed multiple biologics underwent cervical VNS. The treatment led to significant clinical improvements, including marked reductions in key inflammatory cytokines. VNS effectively suppressed peripheral TNF production, reduced disease severity, and improved scores on the Disease Activity Score‐28, without any severe adverse events reported. Subsequent studies confirmed these findings. A 2020 pilot trial using a miniaturized VNS device demonstrated that daily stimulation was safe and well tolerated and significantly improved RA signs and symptoms compared with sham stimulation [722]. These studies collectively confirm that activation of the inflammatory reflex via VNS can modulate autoimmune arthritis in humans by reducing cytokine levels and joint inflammation. This highlights VNS as a precision “bioelectronic medicine” alternative to conventional systemic immunosuppressive therapies in RA.

Given the extensive innervation of the gut by the vagus nerve and its immunoregulatory properties, VNS has also been explored in the treatment of IBD, including Crohn's disease and ulcerative colitis. In experimental colitis models, VNS has been shown to alleviate intestinal inflammation. For example, chronic VNS significantly reduced colitis severity, lowered tissue levels of TNF‐α and IL‐6, and improved autonomic balance [723]. Encouraged by these preclinical results, clinical investigations have begun. In a preliminary study of nine patients with moderate Crohn's disease, 1 year of cervical VNS led to clinical remission in 55% of patients and endoscopic remission in two‐thirds. These improvements were accompanied by significant reductions in inflammatory biomarkers, including CRP and fecal calprotectin [62]. Patients also showed restored vagal tone and a cytokine profile shift toward anti‐inflammatory states, with notable declines in IL‐6, IL‐12/23, and TNF‐α. Importantly, the treatment was well tolerated, with many patients reporting reduced abdominal pain and improved overall well‐being.

Supporting evidence also comes from case series and ongoing trials using noninvasive VNS approaches. A recent study in pediatric ulcerative colitis patients found that twice‐daily transcutaneous auricular VNS significantly improved bowel habits and abdominal pain. This form of neuromodulation is believed to “calm” proinflammatory cytokine activity within the gut [724]. Although these findings remain preliminary, a 2025 review on neuromodulation for IBD concluded that VNS holds substantial promise—especially for patients unresponsive to conventional anti‐inflammatory therapies [725]. Taken together, these studies position VNS as a promising treatment strategy for IBD, offering a novel means to recalibrate intestinal immunity via the GBA.

VNS has also been explored in the context of neurodegenerative diseases, based on its neuromodulatory effects on both the brain and the immune system. Mechanistically, VNS activates brainstem centers and promotes the release of key neurotransmitters—for example, vagal stimulation enhances LC activity and triggers the release of cortical NE. This, in turn, strengthens synaptic plasticity in memory‐related circuits while simultaneously dampening neuroinflammatory responses [726]. These dual effects provide a therapeutic rationale for the use of VNS in AD, in which neuroinflammation and synaptic dysfunction are believed to be major contributors to cognitive decline. Several small‐scale clinical studies have yielded encouraging preliminary signals. In an open‐label trial involving patients with mild to moderate AD, continuous implantable VNS therapy over 6–12 months was associated with stable or modestly improved cognitive function in a subset of participants [727]. Notably, after 1 year of VNS treatment, approximately 41% of patients demonstrated improvement or no decline on the Alzheimer's Disease Assessment Scale‐Cognitive Subscale, while around 71% showed stability or improvement on the Mini‐Mental State Examination—despite the typically progressive nature of AD. Importantly, these patients exhibited no significant deterioration in mood or daily functioning, and VNS was generally well tolerated. Although these early studies lacked control groups, they suggest that VNS may contribute to cognitive stabilization or deceleration of disease progression.

Ongoing research is investigating whether noninvasive VNS can enhance central noradrenergic tone and anti‐inflammatory signaling in a manner that meaningfully alters the trajectory of neurodegeneration [719]. If successful, VNS could serve as an adjunct or alternative therapy that engages neuro‐immune mechanisms to enhance the efficacy of standard treatments, reduce neuroinflammatory damage, and promote neuronal resilience.

VNS has been established as a therapeutic modality for certain neuropsychiatric disorders, most notably treatment‐resistant depression (TRD). In fact, the U.S. Food and Drug Administration (FDA) has approved the use of implanted cervical VNS devices as adjunctive therapy for chronic depression in patients who have failed to respond to conventional antidepressant medications. The neuromodulatory and immunoregulatory effects of VNS are believed to contribute to its antidepressant efficacy. Afferent vagal signals project to the NTS in the brainstem, which in turn communicates with limbic and cortical regions involved in emotion regulation. This pathway influences monoaminergic nuclei such as the raphe nuclei and LC, as well as other neural circuits implicated in the pathophysiology of depression [728]. Increasing evidence indicates that excessive inflammation is closely linked to MDD, with many patients exhibiting elevated levels of proinflammatory cytokines such as IL‐6 and TNF—molecules known to drive “sickness behavior” and depressive symptoms [691, 729]. VNS may counteract this through activation of the inflammatory reflex, thereby suppressing the production of these cytokines [719]. Animal studies have demonstrated that vagus nerve activation inhibits cytokines including TNF, IL‐1β, HMGB1, and IL‐6 via α7nAChR‐mediated pathways [593]. Accordingly, it has been proposed that “the antidepressant benefit of VNS may be attributable, in part, to its ability to suppress proinflammatory cytokines” [730]. Some clinical observations support this hypothesis: in patients who respond well to chronic VNS therapy, levels of peripheral inflammatory biomarkers tend to decline over time [731]. However, the primary mechanism of VNS in depression is still thought to involve modulation of central neural circuits—including improvements in neuroplasticity, neurotransmission, and HPA axis function—while immunomodulation may serve as a synergistic mechanism [717]. In clinical practice, VNS is typically used as an adjunct to standard antidepressant treatment, primarily for individuals with treatment‐resistant depression [732]. Long‐term studies have shown that approximately 30–40% of these patients experience significant and sustained improvements in mood with VNS therapy [733]. This success story in psychiatry highlights the intimate connection between neural circuitry and immune status, and demonstrates that VNS can simultaneously target both brain function and peripheral inflammation—offering a dual‐action approach in disorders such as depression.

A rapidly advancing frontier in neuro‐immune interactions is the application of VNS in oncology. Growing evidence suggests that vagal activity can influence tumor progression and immune surveillance [734]. Several epidemiological studies have reported that cancer patients with higher baseline vagal tone—often measured via heart rate variability (HRV)—tend to have better prognoses and longer OS. Remarkably, this association appears to be independent of tumor stage or treatment modality in certain cases. Conversely, animal studies have shown that disrupting vagal signaling through vagotomy accelerates tumor growth and metastasis, whereas animals with intact or naturally heightened vagal tone exhibit slower tumor progression and reduced metastatic spread [735]. These findings support a protective role of the vagus nerve in cancer, potentially mediated by suppression of protumor inflammation and sympathetic stress responses. Building on this concept, researchers have proposed harnessing VNS to modulate the TME and boost antitumor immunity. One hypothesis is that VNS, through activation of the inflammatory reflex, may help convert immunologically “cold” tumors (characterized by immune suppression) into “hot” tumors (characterized by immune activation). For instance, VNS can suppress the production of key protumorigenic cytokines such as IL‐6, IL‐1β, and TNF‐α—critical drivers of tumor‐associated inflammation—thereby enhancing T cell‐mediated immune responses [736]. More recently, it has been proposed that VNS could be combined with immune checkpoint inhibitors as a novel therapeutic strategy. By reducing immunosuppressive cytokine levels while enhancing T cell activity, VNS may improve the response to checkpoint blockade therapies [736]. While still in exploratory stages, there are already proposals for launching Phase II clinical trials to evaluate whether VNS in combination with standard therapies could improve outcomes in refractory cancers such as GBM. Fundamentally, cancer has been reframed as a state of “immune dysautonomia,” wherein impaired vagal function contributes to immune evasion by tumors. VNS may help correct this imbalance by restoring autonomic–immune homeostasis [41]. Although systematic clinical data remain limited, VNS is gaining attention as a low‐toxicity, adjunctive strategy to existing cancer therapies—aimed at suppressing tumor‐promoting inflammation and reactivating antitumor immune surveillance systems.

From a translational medicine standpoint, VNS has matured considerably with advancements in device engineering and clinical standardization. The traditional approach involves an implantable cervical VNS device, in which electrodes are wrapped around the left cervical vagus nerve and connected to a pulse generator, similar to a cardiac pacemaker, implanted in the chest [737]. This invasive modality has been used for decades in epilepsy and depression, with over 100,000 implants performed globally. Its long‐term safety has been well established, with generally good tolerability and no consistent evidence of chronic immunosuppression or organ damage [738]. This same device format has been repurposed in clinical research for neuroinflammatory conditions such as RA and Crohn's disease [739]. For example, in the first clinical trial of VNS in RA conducted by Koopman et al. in 2016, patients received intermittent cervical VNS using the Cyberonics device, with one to four daily stimulations [61]. Meanwhile, advances in biomedical engineering have led to the development of miniaturized implantable VNS systems tailored for inflammatory diseases. One such device, developed by SetPoint Medical, has shown promising feasibility and safety in RA pilot studies [599]. Perhaps more transformative is the emergence of noninvasive VNS (nVNS) technologies, which stimulate vagal afferents through the skin without the need for surgery. The conventional method employs an implantable cervical vagus nerve stimulator, where electrodes are coiled around the left cervical vagus nerve and attached to a pulse‐generating unit. This generator, resembling a heart pacemaker, is surgically placed in the chest [737]. In trials for IBD and other disorders, patients using handheld, external VNS devices—such as those that stimulate the tragus—have shown favorable treatment responses, significantly improving the accessibility of VNS therapy [724]. In most clinical trials, VNS is administered as an adjunct to standard care rather than as a replacement. For instance, in RA studies, patients typically continue their background therapies, including conventional disease‐modifying antirheumatic drugs. Many participants who remained refractory despite multiple biologics achieved notable improvement after initiating VNS [599]. Similarly, in pilot studies of Crohn's disease, VNS has provided additive therapeutic benefit when used alongside standard treatments [62]. In psychiatry, VNS is already approved as an adjunctive therapy for treatment‐resistant depression, where patients continue antidepressants while receiving chronic neuromodulation via implanted VNS devices to improve mood regulation [740]. This supplemental application model highlights the potential for VNS to synergize with pharmacologic and biologic therapies. By dampening inflammatory mediators and modulating autonomic output, VNS may enhance therapeutic efficacy or permit dose reduction. This synergistic potential is especially intriguing in oncology, where animal models are currently exploring the combination of VNS with cancer immunotherapies—including immune checkpoint inhibitors and adoptive T cell therapies—to augment immune cell infiltration and tumor control through concurrent activation of neuro‐immune anti‐inflammatory circuits [736, 741].

Overall, the therapeutic landscape for VNS is rapidly expanding. Ongoing clinical trials across a range of conditions—including autoimmune arthritis, IBD, heart failure, epilepsy, and depression—are actively investigating optimal stimulation parameters and patient selection criteria to maximize clinical benefit. As a form of bioelectronic medicine, VNS occupies a unique position within the neuro‐immune interface. It offers a novel strategy to treat immune‐driven diseases by targeting the so‐called “molecular inflammatory reflex” via neuromodulation. With continued accumulation of clinical evidence and advancements in device technologies, VNS holds strong potential to become a versatile therapeutic platform across diverse disease domains—including RA, AD, and cancer—anchored in the modulation of neuro‐immune pathways.

#### 5.2.2 Optogenetic Modulation of Neural Circuits

Optogenetic neuromodulation employs genetically encoded light‐sensitive ion channels or pumps—such as excitatory channelrhodopsins or inhibitory halorhodopsins/ archaerhodopsins—to selectively control specific neurons with millisecond precision [742, 743]. By targeting defined cell types within specific brain regions or circuits and delivering light via implanted optical fibers or LEDs, neural firing can be activated or silenced, thereby modulating downstream physiological responses [744]. In the context of neuro‐immune interactions, optogenetics enables precise control of the vagus nerve or brainstem circuits to regulate the “inflammatory reflex” or other stress–immune axes [21]. Under blue light stimulation, channelrhodopsins (e.g., ChR2 variants) depolarize neurons, whereas halorhodopsins (e.g., NpHR) or archaerhodopsins hyperpolarize neurons under yellow or green light, thereby inhibiting neuronal activity [745, 746, 747]. In experimental setups, these opsins are typically expressed using Cre‐dependent viral vectors in transgenic mice, allowing for long‐term neural control in disease models [748, 749]. Activation or inhibition of specific neural circuits alters the release of neurotransmitters such as ACh, NE, or CRH, thereby influencing immune cell functions [750, 751]. Applications of optogenetics have uncovered key mechanisms linking neural activity to immune outcomes. For example, stimulation of cholinergic neurons in the DMN of the vagus nerve significantly activates the “CAP” [750]. In one study, ChAT–Cre mice expressing ChR2 in DMN neurons received 473 nm blue light stimulation in a model of endotoxemia to activate vagal output. This induced action potentials in the splenic nerve and led to a marked reduction in serum TNF levels [752]. Similarly, in a pancreatitis model, targeted activation of cholinergic neurons in the left DMN significantly alleviated caerulein‐induced acute pancreatitis. Stimulated mice showed reduced serum amylase levels and decreased pancreatic inflammatory cytokines. Notably, this anti‐inflammatory effect was abolished by vagotomy or blockade of the α7nAChR [750]. These findings provide direct evidence that optogenetic activation of vagal output via ChR2 can suppress systemic inflammation through established neuro‐immune regulatory pathways. In the gut, Rahman et al. demonstrated that intestinal cholinergic neurons also exert anti‐inflammatory effects. Using ChAT–Cre; ChR2 mice implanted with LED probes in the colon, they applied daily blue light stimulation in a DSS‐induced colitis model, which significantly ameliorated disease severity and reduced proinflammatory cytokine levels [753]. In contrast, ablation of these neurons exacerbated inflammation. Mechanistically, ACh released by enteric neurons suppressed LPS‐induced expression of inflammatory cytokines in macrophages in vitro, illustrating a classic cholinergic anti‐inflammatory reflex [100]. Collectively, these studies highlight that optogenetic activation of vagal or intestinal cholinergic circuits can effectively alleviate autoimmune and inflammatory diseases by suppressing TNF and other cytokine signaling pathways.

Representative optogenetic targets within neuro‐immune circuits include: (1) Cholinergic output neurons of the vagus nerve (originating from the DMN and its peripheral branches). Activation of vagal fibers originating from the DMN using ChR2 enhances splenic nerve activity, engages the α7nAChR pathway in the spleen, and suppresses TNF release [752, 754]. Conversely, optogenetic inhibition of vagal output can be used to investigate how reduced cholinergic tone may exacerbate inflammation [723, 755]. (2) CRH neurons in the hypothalamus and stress‐related regions. CRH‐expressing neurons are located in the PVN of the hypothalamus and in the extended amygdala (CeA/CeM), where they participate in HPA axis activation and stress responses [756]. Although optogenetic studies specifically linking PVN–CRH neurons to immune regulation remain limited, one study identified a CeM–CRH→LPGi→SNS pathway that connects anxiety to tumor progression [757]. In this model, optogenetic inhibition of CeM–CRH neurons (via ChR2 deactivation) suppressed breast tumor growth and reduced anxiety‐like behaviors, whereas activation of the same neurons promoted tumor progression. Mechanistically, this pathway modulated intratumoral sympathetic innervation and NE levels, ultimately influencing antitumor immunity. These findings suggest that optogenetic manipulation of stress‐responsive neurons (such as CRH neurons) can modulate peripheral immune surveillance. (3) The LC–NE circuit. The LC is a diffuse noradrenergic nucleus that regulates arousal and sets the baseline tone for immune activity [758, 759]. While most current studies employ chemogenetic approaches, analogous optogenetic strategies can be applied to regulate both central and peripheral immune states. For instance, in EAE—a model of CNS autoimmunity—pharmacological activation of LC–NE neurons alleviates neuroinflammation and demyelination [760]. Similarly, optogenetic stimulation of the LC or its projection areas (such as the cortex or hypothalamus) is expected to suppress microglial activation and promote an anti‐inflammatory immune bias, potentially through NE acting on β_2_‐ARs expressed by immune cells [761, 762]. In summary, optogenetic studies in models of autoimmune and neurodegenerative diseases have demonstrated that precise neural control can significantly alter disease trajectories. Beyond colitis and pancreatitis, vagal neuromodulation has also been validated in RA and EAE—both classic autoimmune models—using electrical or chemogenetic methods, with optogenetics offering more refined spatial and temporal resolution [763, 764]. In psychiatric disease models, sustained activation of PVN–CRH neurons has been shown to induce GC release and depressive‐like behaviors [765]. These stress‐induced changes ultimately feedback to regulate immunity, as chronic stress exerts broad immunosuppressive or immune‐skewing effects. These findings underscore the potential of optogenetics as a tool to dissect the mechanisms underlying neuro‐immune interactions in affective disorders.

In malignant diseases, neuromodulation may hold therapeutic potential. As noted above, silencing CRH‐driven anxiety circuits in a mouse model of breast cancer was shown to slow tumor progression [757]. More broadly, optogenetic tools are being developed to enhance cancer immunotherapy. For instance, Tan et al. reviewed strategies of “opto‐immunomodulation,” in which T cells or DCs are engineered to express light‐sensitive channels (e.g., Opto‐CRAC or light‐activated chemokine receptors), enabling the control of calcium signaling, cell migration, or cytokine release through blue or red light pulses [766, 767]. Although these approaches primarily focus on immune cells themselves, they underscore the potential for synergy between neuromodulation and immunotherapy. For example, one can envision combining optogenetic stimulation of the vagus nerve or LC—aimed at reducing systemic immunosuppression by lowering IL‐6 and TNF—with checkpoint inhibitors or tumor vaccines to convert “cold” tumors into “hot” ones. Indeed, it has been proposed that VNS may activate the cholinergic anti‐inflammatory reflex to suppress IL‐6‐driven tumor immune evasion, thereby sensitizing otherwise resistant gliomas to immune checkpoint inhibitors [736]. In a lung cancer model, electrical stimulation of the vagus nerve increased the number of tumor‐infiltrating T cells and counteracted radiotherapy‐induced immunosuppression. Preliminary clinical data in early‐stage lung cancer also suggest that nVNS may reduce neutrophil levels and increase NK cell activity [741]. While most of these studies have employed electrical VNS, optogenetic VNS in animal models offers the advantage of dissecting which specific vagal fiber populations mediate these effects, thus enabling fine‐tuning of stimulation paradigms to optimize synergy with pharmacological therapies [768].

Optogenetic neuromodulation can also be combined with small‐molecule drugs or biologics to enhance therapeutic efficacy. For example, coadministration of α7nAChR agonists, such as GTS‐21, can potentiate the anti‐inflammatory effects elicited by ChR2‐driven vagal nerve pulses. Conversely, blocking GCRs helps to elucidate the role of the HPA axis in optogenetically induced stress models [769, 770, 771]. In contrast, certain compounds that increase neuronal circuit sensitivity—such as AR agonists targeting the LC—can amplify the effects of optogenetic stimulation [772, 773]. In cancer treatment, optogenetically engineered immune cells capable of light‐controlled cytokine release—such as the FLICs hydrogel system—can be combined with PD‐1 antibodies to enhance therapeutic outcomes [774]. Although these strategies remain experimental, the central concept is compelling: by precisely tuning the timing of neural activity, it may be possible to optimize the spatial and temporal delivery of immunotherapies while minimizing off‐target effects. While optogenetic neuromodulation is a powerful research tool, several challenges remain for its clinical translation in humans. Opsin‐based therapies require gene delivery—typically via AAV vectors—to the target neurons, raising concerns regarding safety and immunogenicity [775]. Both viral capsids and microbe‐derived opsins may provoke immune responses: in rodents, antibodies against ChR2 have been detected following AAV‐mediated delivery, leading to eventual loss of expression [776]. To achieve clinically feasible human applications, opsin delivery systems must be further developed to ensure high efficiency, cell‐type specificity, and minimal immunogenicity. This includes engineering red‐shifted opsins to allow deeper tissue penetration using near‐infrared light. Light delivery poses another major hurdle: in the human brain or deep internal organs, conventional optical fibers may fail to adequately illuminate all target cells. Current solutions under development include implantable micro‐LED devices, wireless optoelectronic probes, and upconversion nanoparticles that convert near‐infrared light into opsin‐activating wavelengths [777]. However, nonhuman primate studies indicate that issues such as tissue transduction efficiency and long‐term stability remain limiting factors, suggesting that strategies will need to be reengineered for successful human application [778].

In summary, optogenetic modulation of neural circuits offers an exceptionally precise tool for investigating and potentially treating neuro‐immune disorders. This technology has already elucidated several well‐defined mechanistic pathways—such as the DMN cholinergic–splenic nerve–anti‐inflammatory axis, and the CeM–CRH–sympathetic nerve–tumor progression circuit. Future research may focus on integrating optogenetic neuromodulation with targeted immunotherapies, including drugs, vaccines, and immune checkpoint inhibitors, to achieve synergistic control of inflammation or tumor immunity. However, successful clinical translation will require overcoming key obstacles: the limitations of gene therapy, the development of safe and effective light delivery systems, and the need to ensure that neuronal activation or inhibition does not produce unintended perturbations within broader neural networks. Despite these challenges, optogenetics continues to provide a highly promising blueprint for next‐generation neuromodulatory strategies. With advances in photopharmacology and neural interface technologies, optogenetic approaches may ultimately become applicable to human neuro‐immune disease therapy.

### 5.3 Clinical Translation Challenges

Despite the conceptual and experimental advances in targeting the neuro‐immune axis, translating these strategies into effective clinical therapies remains challenging. Neuro‐immune interactions are highly context dependent, influenced by disease type, neural circuitry, immune cell phenotype, and individual patient variability. Consequently, two major barriers must be addressed for successful clinical implementation: (i) the identification of robust biomarkers that enable patient stratification and predict responsiveness to neuro‐immune interventions, and (ii) the rational design of combinatorial therapies that integrate neuromodulation with existing immunotherapeutic or anti‐inflammatory regimens. This section explores both aspects, highlighting key insights from preclinical studies and ongoing clinical trials to guide future translation efforts.

#### 5.3.1 Biomarker Development for Patient Stratification

Neuro‐immune disorders are increasingly recognized as diseases characterized by specific molecular and cellular biomarkers, which can reflect immune activation, neural injury, or the state of autonomic nervous system tone [779]. For example, in MS, CSF and blood levels of neurodegeneration‐associated biomarkers—such as neurofilament light chain (NFL) and heavy chain (NFH—have been validated as strong predictors of disease severity [780, 781]. Tolentino et al. reported that elevated levels of NFH, NFL, and soluble TREM2 (a marker of microglial activation) in the CSF at the time of diagnosis were all associated with worse MS severity scores [781]. Thus, high levels of neurofilaments or sTREM2 can help identify patients at risk of rapid disease progression and may guide early initiation of high‐efficacy immunotherapies. Simultaneously, TSPO–PET imaging of microglial activation is being explored as a potential biomarker in MS. As reviewed by Airas et al., PET radioligands targeting the 18 kDa translocator protein (TSPO) can detect diffuse microglial inflammation beyond focal lesions; notably, TSPO signal intensifies during progressive phases of MS and may serve to monitor treatment efficacy in clinical trials [782, 783]. These molecular imaging biomarkers complement traditional MRI‐based assessments of neuroinflammation and can facilitate patient stratification—for example, distinguishing patients with chronic inflammatory activity—to inform neuroprotective or anti‐inflammatory therapeutic strategies.

In systemic autoimmune diseases, classical inflammatory biomarkers also help define patient subgroups. For example, in RA and SLE, serum levels of CRP and cytokines such as IL‐6 and TNF‐α are commonly used as indicators of disease activity [784]. In RA, interventions targeting the neuro‐immune axis have begun to emerge. In a clinical trial, Koopman et al. demonstrated that implanted VNS significantly suppressed the production of TNF, IL‐1β, and IL‐6, leading to clinical improvement in RA symptoms [61]. Although specific predictors of VNS responsiveness have yet to be established, patients with treatment‐refractory RA and high inflammatory burden may be selected for neuromodulatory therapy based on their cytokine profiles [600]. In central autoimmune syndromes, pathogenic autoantibodies serve not only as diagnostic tools but also as markers for patient stratification. For instance, antibodies against aquaporin‐4 are diagnostic for NMO and can predict responsiveness to B cell‐targeted therapies. Similarly, in limbic encephalitis, anti‐NMDAR antibodies inform immunotherapy selection and prognosis [785, 786]. Collectively, current molecular biomarkers—including neurofilaments, cytokines, and autoantibodies—are increasingly used to classify subtypes of neuro‐immune diseases and to guide the selection and intensity of therapeutic interventions. An increasing body of evidence suggests that neuropsychiatric disorders also exhibit immune‐related endophenotypes [787]. In MDD, dozens of studies have linked peripheral inflammatory biomarkers—such as CRP, IL‐6, IL‐1β, IL‐8, and TNF‐α—with symptom severity and treatment response [788]. For example, a review by Orsolini et al. noted that elevated CRP levels are typically associated with more severe and treatment‐resistant depressive symptoms. Approximately one‐third of patients with depression exhibit a low‐grade inflammatory state, constituting the so‐called “inflammatory depression” subtype [789]. Similarly, a meta‐analysis found that patients with elevated baseline levels of CRP and IL‐8 had significantly poorer responses to standard antidepressant medications [790]. These findings suggest that inflammatory biomarkers may facilitate stratification of patients with depression. In individuals with high CRP or IL‐6 levels, anti‐inflammatory treatments or adjunctive therapies targeting the immune system—such as cytokine antagonists, statins, or specific antidepressants with anti‐inflammatory properties—may be prioritized.

Neuroimaging can also serve as a stratification tool in neuropsychiatric disorders. Functional MRI and PET studies have identified distinct alterations in brain activity patterns among patients with inflammatory depression—for example, disrupted connectivity within emotion‐regulation circuits—which correlate with cytokine levels [791, 792, 793]. Although not yet routinely applied in clinical settings, such imaging‐based endophenotypes—including TSPO–PET for activated microglia and PET tracers for reactive astrocytes—show promise in identifying “neuroinflammatory” states within psychiatric populations [794]. Taken together, integrating molecular biomarkers (e.g., CRP, IL‐6, IL‐8) with imaging or electrophysiological parameters may enable the definition of specific neuropsychiatric disease subtypes and facilitate individualized neuro‐immune therapies. Autonomic nervous system function is also emerging as a functional biomarker of neuro‐immune status. HRV, a noninvasive index of vagal tone, has been associated with disease progression in a variety of inflammation‐related conditions [795]. Notably, higher resting‐state HRV has been linked to improved cancer outcomes: studies show that cancer patients with elevated HRV have significantly longer survival rates [796], suggesting that a well‐preserved vagal anti‐inflammatory reflex may confer increased resilience. Furthermore, the concept of a neuroimmunomodulation index (NIM)—defined as the ratio of vagally mediated HRV to CRP (or IL‐6)—has been proposed [797]. In two large prospective cancer cohorts, a higher NIM (i.e., greater vagal activity relative to systemic inflammation) was significantly associated with slower tumor progression and longer survival. These composite biomarkers hold strong translational potential: patients with low NIM or HRV may particularly benefit from interventions such as VNS or cholinergic agonists to enhance their anti‐inflammatory reflex.

Similarly, physiological indicators such as altered baroreflex sensitivity or skin conductance have been explored in MS and epilepsy as surrogate markers of neuro‐immune interaction [798, 799]. With continued advancement in bioelectronic medicine, these physiological biomarkers of neuro‐immune balance may in the future inform the optimal timing and targeting of neuromodulatory interventions.

Biomarkers are increasingly being utilized to personalize therapeutic strategies targeting the neuro‐immune axis. For instance, VNS has been approved by the FDA for conditions such as epilepsy and depression, and ongoing studies are now focused on identifying predictors of treatment response [737]. Preclinical evidence suggests that baseline inflammatory status may influence the efficacy of neuromodulation. In RA, for example, patients with high disease activity and elevated cytokine levels exhibit significant reductions in TNF levels and clinical symptom improvement following VNS [800, 801]. Similarly, patients with MDD and elevated CRP may be more likely to benefit from adjunctive anti‐inflammatory treatments or VNS [731, 802]. In MS, quantifying certain molecules such as IL‐17 or BDNF may aid in stratifying patients for neuro‐targeted interventions, although this remains hypothetical at present [803, 804]. While optogenetic neuromodulation remains limited to animal models, its underlying rationale also emphasizes the importance of biomarkers: it enables precise control of specific neural circuits within immune organs but depends on the expression of certain receptors or molecular markers to identify patients who may benefit from cell‐specific, light‐controlled therapies [805]. Pharmacological neuro‐immune interventions likewise rely on biomarker‐guided stratification. For example, patients with type 2 (Th2‐driven) asthma and elevated eosinophil counts are candidates for IL‐5‐targeting biologics [806]. Although this principle originates outside the CNS, it is equally applicable to CNS disorders: in depression, elevated inflammatory markers may indicate a better response to cytokine inhibitors or glial‐targeting agents such as minocycline or anti‐TNF therapies [693, 807]. In MS, emerging biomarkers related to the neuro‐immune axis—such as S100B or metabolites of the kynurenine pathway—are under investigation as potential predictors of responsiveness to immunomodulatory or regenerative therapies [808, 809]. Overall, biomarker‐based approaches are increasingly becoming essential for determining which patients are most likely to benefit from neuro‐immune interventions—ranging from device‐based neuromodulation to targeted pharmacological therapies.

In oncology, the search for biomarkers that can guide neuro‐immune interventions is emerging as a promising area of research. Inflammation‐related markers—such as CRP, IL‐6, and the neutrophil‐to‐lymphocyte ratio—are already widely recognized as prognostic indicators and may be linked to neural circuit activity [149, 810, 811]. For example, chronic stress and activation of the HPA axis—detectable via cortisol levels or gene expression profiles such as TSC22D3—have been associated with poor responses to immunotherapy [812]. Parasympathetic tone also holds prognostic value: as discussed earlier, HRV and the NIM have been shown to correlate with survival outcomes in cancer patients [734, 813]. Expression of neurotransmitter receptors within tumors may further serve as a stratification strategy. In estrogen receptor‐negative breast cancer, for instance, high expression of the β_2_‐AR is associated with reduced tumor‐infiltrating lymphocytes and represents an independent negative prognostic factor [814]. These findings suggest that elevated β_2_‐AR expression may serve as a biomarker to identify patients likely to benefit from β‐blockers or other sympathetic‐suppressing interventions. Similarly, other neural‐related markers—such as α7nAChRs, DR subtypes, or uptake of vagal nerve tracers—are under investigation as potential predictors of response to cancer immunotherapy or neuromodulatory approaches [361, 815, 816]. Although the integration of neuro‐immune biomarkers into oncology remains in its early stages, initial studies are encouraging. For example, a composite “vagal neuro‐immune index” combining HRV and CRP has been associated not only with mortality risk but also with tumor growth kinetics. Such indices could be used in the future to stratify patients and guide interventions like tVNS to activate CAPs and enhance immunotherapeutic efficacy [817]. Likewise, emerging experimental measures—such as intratumoral nerve density or neural crest‐related markers—may help predict which tumors are more likely to respond to denervation‐based strategies [818, 819].

In summary, a wide range of biomarkers—spanning systemic inflammatory mediators, autonomic function indicators, and tumor‐specific neural receptor expression—are being explored to identify cancer patients most likely to benefit from therapies targeting the neuro‐immune interface.

#### 5.3.2 Combinatorial Approaches

Emerging evidence suggests that targeted neuromodulation can synergize with cancer immunotherapies and immunoregulatory treatments for inflammatory diseases [41, 152]. The scientific rationale stems from the profound influence of the nervous system on immune function—commonly referred to as the “neuro‐immune reflex” [820]. For instance, excessive sympathetic (adrenergic) signaling under stress conditions can promote the development of an immunosuppressive TME, thereby weakening antitumor immunity [149]. In contrast, activation of parasympathetic pathways—such as the vagus nerve—can trigger the “cholinergic anti‐inflammatory reflex,” suppressing cytokine release from macrophages and other immune cells [591]. Multiple neuromodulation strategies are currently under active investigation, including VNS, optogenetic activation of specific neural circuits, β‐AR antagonists, and direct electrical stimulation, with the goal of leveraging neuro‐immune interactions for therapeutic benefit [41, 716, 821]. VNS, in particular, can induce ACh release from T cells, which then binds to α7nAChRs on macrophages to inhibit proinflammatory cytokine production [822]. This CAP represents a central mechanism by which neuromodulation can restrain excessive inflammation and restore immune homeostasis.

Preclinical studies in tumor models have demonstrated that combining neuromodulation with immunotherapy can effectively reshape the tumor immune microenvironment [741]. Research indicates that cancer‐induced nerve injury (CINI) remodels the TME through neuro‐immune mechanisms and serves as a key factor in resistance to anti‐PD‐1 therapy. Targeting CINI can effectively enhance treatment response: in immunocompetent mice, surgical denervation combined with anti‐PD‐1 significantly improved tumor control (*p* = 0.0027); conditional knockout of ATF3 in nociceptive neurons reduced melanoma volume by 40% and increased intratumoral IFNγ⁺CD8⁺ T cells threefold; knockout of IFNα receptor (Ifnar1^−^/^−^) abolished the detrimental effects of ethidium bromide‐induced demyelination; while combination therapy with anti‐PD‐1 and anti‐IL‐6R reduced viable tumor cells by 65% [435].These findings establish CINI as a potentially targetable pathway in immunotherapy‐resistant cancers. Inhibition of β‐adrenergic stress signaling—via pharmacological β‐blockers or genetic ablation—can convert tumors into more immunologically active states by reducing immunosuppressive myeloid cells such as MDSCs and TAMs, while enhancing infiltration of effector T cells [397, 823]. Notably, suppression of β2‐adrenergic signaling, either by reducing cold‐induced stress or using propranolol (a nonselective β‐blocker), significantly increased intratumoral CD8⁺ T cell numbers and effector function, decreased PD‐1 expression, and improved response to anti‐PD‐1 therapy in mice [404]. Similarly, propranolol not only inhibited tumor growth but also eliminated MDSCs and repolarized macrophages, thereby enhancing the efficacy of CTLA‐4 blockade [823]. Other neuromodulation strategies have also demonstrated immune‐enhancing potential. For example, applying electric fields can induce immunogenic cell death and cytokine release in tumors. In murine models, low‐intensity currents or nanosecond pulsed stimulation significantly reduced tumor volume only in immunocompetent mice—ineffective in T cell‐deficient models—indicating a mechanism dependent on intact immunity [824, 825]. Moreover, electroporation combined with cytokine gene therapy has shown potent antitumor effects in animals by enhancing local transfection of plasmids encoding cytokines (e.g., IL‐12), thereby promoting NK and T cell infiltration into the tumor [826, 827]. Even more sophisticated technologies such as optogenetics have been applied in preclinical models to precisely stimulate neural pathways that regulate immune activity (e.g., vagal efferent fibers), allowing mechanistic dissection and fine‐tuned control of optimal neuro‐immune circuits with minimal off‐target effects [750, 802, 828]. Support for combined neuromodulation–immunotherapy strategies also come from nononcologic disease models. In rodents, VNS‐via implanted electrodes or transcutaneous methods‐effectively suppresses proinflammatory cytokine release during endotoxemia and tissue injury, thereby preventing fatal cytokine storms [829, 830]. This neurogenic anticytokine effect has also been validated in early clinical studies. In patients with RA, implanted VNS devices activated the inflammatory reflex, leading to marked reductions in TNF, IL‐1β, and IL‐6 in response to endotoxin challenge, which correlated with clinical improvement [61, 599]. Similarly, a clinical trial in sepsis showed that 5 days of transcutaneous auricular VNS was well tolerated, significantly reduced TNF‐α and IL‐1β, and increased the anti‐inflammatory cytokine IL‐10‐outperforming the sham stimulation group [721]. Collectively, these findings indicate that neuromodulation broadly regulates immune responses‐from dampening cytokine storms to enhancing host defenses‐providing a strong mechanistic rationale for combining it with immunotherapy. Early‐phase clinical trials are already testing such combinations. A notable example is a phase I trial combining propranolol (a nonselective β‐blocker) with pembrolizumab (anti‐PD‐1 antibody) in patients with metastatic melanoma [632]. Based on the stress‐immunity hypothesis, this trial aimed to relieve adrenergic immunosuppression and improve checkpoint inhibitor efficacy. The combination was well tolerated, with no dose‐limiting toxicity and an objective response rate of 78% (seven out of nine patients achieving partial or complete response). Responders exhibited favorable immunologic biomarker changes (increased IFN‐γ, decreased IL‐6), suggesting that β‐blockade made the TME more permissive to T cell activity. Although limited by small sample size, this high response rate surpassed historical outcomes with PD‐1 monotherapy, prompting further evaluation of propranolol 30 mg twice daily as the recommended phase II dose. Additional clinical studies are underway, such as NCT05741164, which is assessing the combination of β_2_‐blockers (e.g., propranolol) with anti‐PD‐1 therapy in breast cancer. Beyond efficacy, neuromodulation is also being investigated as a strategy to mitigate the adverse effects of immunotherapy [736]. For example, VNS or AR blockade has been proposed to control cytokine storms induced by CAR‐T therapy or immune checkpoint inhibitors, potentially enhancing the safety of these powerful interventions [736, 831]. In inflammatory and autoimmune diseases, neuromodulation is being explored as an adjunct to biologic immunotherapies. A representative case is the use of VNS in IBD and arthritis. In an open‐label study of drug‐refractory Crohn's disease, implanted VNS significantly improved clinical remission rates, suggesting synergy with ongoing immunosuppressive regimens to help restore immune balance [739]. In RA, as previously discussed, VNS has been shown to suppress key cytokines such as TNF—a common therapeutic target of many biologics—indicating that bioelectronic stimulation may complement or even reduce the required dosage of anticytokine antibody therapies [800]. Even noninvasive VNS devices (e.g., transcutaneous auricular electrodes) are under investigation for their potential to enhance conventional therapies in SLE and other autoimmune conditions [832]. Although clinical data remain limited for some of these indications, the consistent anti‐inflammatory effects observed in preclinical models and early trials are promising. By reshaping immune cell trafficking and inflammatory mediator profiles, neuromodulation may help create a more favorable immune microenvironment—whether by facilitating T cell infiltration into tumors, converting “cold” tumors into “hot,” or preventing uncontrolled inflammation during treatment. This paves the way for broader implementation of combined neuromodulatory and immunotherapeutic strategies in both oncology and immune‐mediated diseases.

Despite the promising outlook for combining immunotherapy with neuromodulation, several translational challenges must be overcome before widespread clinical application can be achieved: (1) Optimal timing and scheduling of interventions: Determining the appropriate timing and delivery pattern of neuromodulatory interventions is critical. For instance, should neural stimulation be administered continuously, or as pulsed stimulation before or after immunotherapy? How can desensitization of neural circuits be avoided, and how can stimulation be synchronized precisely with windows of immune activation? These are key questions for clinical trial design [152, 833]. (2) Patient selection criteria: It remains unclear which patients are most likely to benefit from adjunctive neuromodulation. Baseline autonomic tone or stress‐related biomarkers—such as elevated NE or reduced vagal tone—may indicate greater responsiveness to sympathetic inhibition or vagal activation [734]. Conversely, if a patient already maintains a favorable neuro‐immune balance, additional intervention may be unnecessary. Establishing robust criteria for patient stratification will help ensure that combined therapies are applied to those most likely to benefit. (3) Standardization of devices and optimization of delivery: Neuromodulatory technologies vary widely—from implantable electrodes to transcutaneous stimulators to pharmacological neuromodulators—making standardization a major challenge. Most approaches remain at the preclinical or early‐phase clinical stage. Critical parameters such as stimulation intensity, target nerve selection, invasiveness (implantable vs. noninvasive), and patient adherence must be optimized. For example, VNS devices must be finely tuned in terms of pulse frequency and amplitude to exert immunoregulatory effects without inducing off‐target side effects. Emerging technologies such as wireless or biodegradable stimulators also require systematic evaluation. (4) Biomarkers for response monitoring: Unlike pharmacologic therapies, it is often difficult to directly assess whether neuromodulation is eliciting the desired immunological effects in real time. Therefore, there is an urgent need for biomarkers that reflect immune responses—such as changes in cytokine levels, immune cell‐specific transcriptional profiles, or even neurophysiological measures like HRV as a proxy for vagal tone. These biomarkers could guide real‐time dose adjustments (e.g., for β‐blockers or stimulation parameters), verify effective modulation of the immune microenvironment, and aid in early‐stage patient stratification by predicting which individuals are likely to mount an immune response to neuromodulation and which may require alternative strategies.

In summary, the integration of immunotherapy with neuromodulation represents a novel and forward‐looking frontier in the treatment of cancer and immune‐mediated diseases. This strategy is grounded in strong mechanistic foundations—the bidirectional communication between the nervous and immune systems—and is supported by compelling preclinical data from tumor and inflammatory disease models. Early‐phase clinical trials, such as those combining β‐blockers with checkpoint inhibitors in melanoma or applying VNS in autoimmune disorders, have provided initial evidence that neuro‐immune combinatorial approaches can enhance therapeutic efficacy without markedly increasing toxicity. By suppressing immunosuppressive signals or dampening excessive inflammation through neuromodulation, clinicians may help the immune system achieve more effective clearance of tumors or pathological immune activity. However, realizing the full clinical potential of this approach will require addressing several translational hurdles, including the design of well‐structured clinical trials and interdisciplinary collaboration among oncologists, immunologists, and bioengineers. If successful, such combination strategies could usher in a new era of therapeutics—one that integrates the nervous and immune systems not as separate entities, but as synergistic partners in disease intervention.

## 6 Conclusions and Future Perspectives

Neuroimmunology has achieved groundbreaking progress over the past 2 decades, fundamentally reshaping our understanding of the interactions between the nervous and immune systems. This review summarizes the molecular mechanisms and biological functions of neuro‐immune interactions, their roles in diseases, and recent advances in related therapeutic strategies. In recent years, breakthroughs in technologies such as chemogenetics, optogenetics, and modified PRV viral tracing have enabled researchers to map the three‐dimensional connectivity of the brain–peripheral nerve–immune axis with unprecedented spatiotemporal resolution and precisely manipulate the functional activity of specific neuronal subpopulations in vivo. The integration of single‐cell multiomics and spatial transcriptomics has allowed fine‐grained molecular‐level analysis of neuro‐immune synapses, while the combination of CRISPR–Cas9 gene editing and metabolic flux analysis has deepened our understanding of the role of metabolic reprogramming in neuro‐immune regulation. Building on these foundational discoveries, neuroimmunomodulatory therapies such as anti‐CGRP monoclonal antibodies and VNS have been successfully translated into clinical practice. However, the field still faces numerous challenges: current research predominantly focuses on static interactions, whereas the spatiotemporal dynamics of neuro‐immune networks (e.g., circadian rhythms, disease‐stage dependency) remain poorly understood. Real‐time monitoring of neurotransmitter release in deep tissues (e.g., BM) is yet to be developed. Significant gaps persist in the interaction maps between neuronal subtypes and immune cell subsets at the single‐cell level, and the mechanisms underlying phenotypic switching of microglia, T cells, and other immune populations in different disease states remain unclear. These limitations severely hinder a deeper understanding of the spatiotemporal specificity of neuro‐immune crosstalk. Additionally, cell‐type‐specific regulatory mechanisms and individual variations in clinical treatment responses demand breakthroughs in novel research tools and therapeutic strategies.

Looking ahead, the advancement of neuroimmunology will rely on the deep integration of basic research, technological innovation, and clinical translation. At the basic research level, key directions include developing next‐generation neurotransmitter dynamic monitoring technologies, constructing humanized organoid models, and deciphering the ultrastructure of neuro‐immune synapses. Specifically, combining two‐photon microscopy with genetically encoded sensors (e.g., GRAB‐neurotransmitter) could enable real‐time visualization of neuro‐immune synapses in vivo; applying CRISPR‐mediated Perturb‐seq screening could systematically identify novel regulatory targets; and establishing brain organoid‐immune cell coculture systems would help simulate complex human microenvironments. On the technological front, noninvasive neuromodulation, smart responsive drug delivery systems, and bioelectronic medical devices are poised for rapid development. In clinical translation, optimizing microglial reprogramming strategies, developing neuropeptide receptor‐specific modulators, and exploring novel therapies such as regional neuromodulation combined with immunotherapy will become research hotspots. Achieving these advances will require interdisciplinary collaboration across computational neuroscience, synthetic biology, microbiome research, and other fields.

With the evolution of research paradigms and technological advancements, neuroimmunology is entering an era of unprecedented opportunity. By constructing personalized neuro‐immune signatures and developing spatiotemporally precise therapeutic strategies, the field holds promise for delivering breakthrough treatments within the next decade for a wide range of refractory diseases, including neurodegenerative disorders, autoimmune diseases, cancer, and psychiatric conditions.

## Author Contributions

Xin Guo: writing—review and editing, writing—original draft, visualization, and conceptualization. Hui Liu: writing—review and editing, writing—original draft, visualization, and conceptualization. Yu‐Jing Song: writing—review and editing and conceptualization. Jian‐Hua Wang: writing—review and editing. Dangfeng Liu: writing—review and editing. Zhi‐Wei Zheng: writing—review and editing. Jia‐Jun Li: writing—review and editing and funding acquisition. Boya Li: writing—review and editing and funding acquisition. An Song: writing—review and editing and funding acquisition. Wei He: funding acquisition and conceptualization. Lei‐Lei Yang: writing—review and editing, funding acquisition, and conceptualization. Shuo Wang: writing—review and editing, writing—original draft, visualization, and conceptualization. All authors have read and approved the final manuscript.

## Conflicts of Interest

The authors declare no conflicts of interest.

## Ethics Statement

The authors have nothing to report.

## Data Availability

The authors have nothing to report.
